# Supplementary material for: Food insecurity among African Americans in the United States: A scoping review
Source: PLoS One. 2022 Sep 12;17(9):e0274434. doi: 10.1371/journal.pone.0274434 (PMC9467341; doi:10.1371/journal.pone.0274434)
Supplement: S1 Appendix — (DOCX) [file pone.0274434.s001.docx]

**Appendix A: Search Strategies**

Table S1. Search string for PubMed®. Interface: PubMed® Legacy, Database coverage dates: mid-1960s to May 20, 2021

| **Search** | **Query** | **Actions** | **Items found:** | **Items found:** |  |
| --- | --- | --- | --- | --- | --- |
|  |  |  | **11/18/2019** | **5/20/2021** |  |
| #1 | "food supply"[MeSH Terms] OR "access to food"[Title/Abstract] OR "dietary inadequacy"[Title/Abstract] OR "food access"[Title/Abstract] OR "food accessibility"[Title/Abstract] OR "food afford*"[Title/Abstract] OR "food assistance"[Title/Abstract] OR "food availability"[Title/Abstract] OR "food choice"[Title/Abstract] OR "food consumption"[Title/Abstract] OR "food deprivation"[Title/Abstract] OR "food desert"[Title/Abstract] OR "food hardship"[Title/Abstract] OR "food insecurity"[Title/Abstract] OR "food insufficien*"[Title/Abstract] OR "food intake"[Title/Abstract] OR "food poverty"[Title/Abstract] OR "food scarcity"[Title/Abstract] OR "food security"[Title/Abstract] OR "food sufficien*"[Title/Abstract] OR "food supply"[Title/Abstract] OR "food utilization"[Title/Abstract] OR "fruit[Title/Abstract] AND vegetable intake"[Title/Abstract] OR "fruit intake"[Title/Abstract] OR "vegetable intake"[Title/Abstract] OR hunger[Title/Abstract] OR malnutrition[Title/Abstract] OR "nutrition security"[Title/Abstract] OR "nutritional status"[Title/Abstract] OR "supermarket access"[Title/Abstract] OR undernourishment[Title/Abstract] | Concept #1: Food insecurity (this search uses a combination of free-text keywords and controlled vocabulary terms [MeSH]) | 151,265 | 170,109 |  |
| #2 | ("African Americans"[Title/Abstract] OR "African American"[Title/Abstract] OR Black[Title/Abstract]) OR African Americans[MeSH Terms] | Concept #2: Population | 182,988 | 204,820 |  |
|  |  |  |  |  |  |
| #3 | #1 AND #2 | Searching for both concepts to appear in the records of databases | 1,829 | 2,121 |  |
| #4 | "Animals"[Mesh] NOT ("Animals"[Mesh] AND "Humans"[Mesh]) | Setting up a search for excluding animal studies | 4,639,963 | 4,831,043 |  |
| #5 | #3 NOT #4 | Excluding animal studies | 1,600 | 1,868 |  |
| **#6** | **#5 Filters: Publication date from 1995/01/01 to 2019/11/18; English; Adult: 19+ years; Young Adult: 19-24 years; Adult: 19-44 years; Middle Aged + Aged: 45+ years; Middle Aged: 45-64 years** | **Applying filters to limit the records by publication year, language, and age** | **738** | **71** |  |
|  | **#5 Filters Updated search: Publication date from 2019/11/19 to 2021/05/20 English; Adult: 19+ years; Young Adult: 19-24 years; Adult: 19-44 years; Middle Aged + Aged: 45+ years; Middle Aged: 45-64 years** |  |  |  |  |

Table S2. Search string for EBSCO Databases, conducted on May 20, 2021. Interface: EBSCO Databases, Database coverage dates: Various (see table below)

| **Databases** | **Limiters** | **Items found: 11/18/19** | **Items found: 5/20/21** |
| --- | --- | --- | --- |
| CINAHL Plus | 1995-2019, English | 1,091 | 115 |
| MEDLINE | 1995-2019, English, Peer reviewed | 744 | 233 |
| PsycINFO | 1995-2019, English, Academic journals | 498 | 25 |
| Health Source: Nursing/Academic Edition | 1995-2019, English, Peer reviewed | 327 | 36 |
| **TOTAL** |  | **2,660** | **409** |

**EBSCO Search strategy:**

( (dietary N5 inadequacy) OR (food N5 (access OR accessibility OR afford* OR assistance OR availability OR choice OR consumption OR deprivation OR desert OR hardship OR insecurity OR insufficien* OR intake OR poverty OR scarcity OR security OR sufficien* OR supply OR utilization)) OR ((fruit OR vegetable) N5 intake) OR hunger OR malnutrition OR "nutrition security" OR "nutritional status" OR (supermarket N5 access) OR undernourishment )

AND ( "African American*" OR Black* ) AND ( adults OR adult OR aged OR elderly )

Table S3. Search string for Web of Science^TM^ (Clarivate). Interface: Web of Science^TM^ (Core Collection), Database coverage dates: 1900-May 20, 2021

| **Search** | **Query** | **Items found** |
| --- | --- | --- |
| #1 | ((TI=(( "access to food" OR "dietary inadequacy" OR "food access" OR "food accessibility" OR "food afford*" OR "food assistance" OR "food availability" OR "food choice" OR "food consumption" OR "food deprivation" OR "food desert" OR "food hardship" OR "food insecurity" OR "food insufficien*" OR "food intake" OR "food poverty" OR "food scarcity" OR "food security" OR "food sufficien*" OR "food supply" OR "food utilization" OR "fruit and vegetable intake" OR "fruit intake" OR "vegetable intake" OR hunger OR malnutrition OR "nutrition security" OR "nutritional status" OR "supermarket access" OR undernourishment )) AND TS=(( "African American*" OR Black* ))) NOT SU=("Veterinary Sciences" OR Agriculture OR Entomology OR Fisheries OR Forestry OR "Plant Sciences" OR Zoology)) | 470 |

**Limiters:**

LANGUAGE: (English)

DOCUMENT TYPES: (Article)

Indexes=SCI-EXPANDED, SSCI, A&HCI, CPCI-S, CPCI-SSH, BKCI-S, BKCI-SSH, ESCI, CCR-EXPANDED, IC Timespan=1995-2019

**Total results:** 398 (72 from updated search on May 20, 2021)
