# Supplementary material for: Food insecurity among African Americans in the United States: A scoping review
Source: PLoS One. 2022 Sep 12;17(9):e0274434. doi: 10.1371/journal.pone.0274434 (PMC9467341; doi:10.1371/journal.pone.0274434)
Supplement: S2 Appendix — (DOCX) [file pone.0274434.s002.docx]

**Appendix B: Characteristics of Included Studies**

Table S4. Identified metrics and definitions of food security (sorted by metrics)

| **Citation** | **Food Security Metric** | **Authors’ Definition of the Food Security Metric** |
| --- | --- | --- |
| Ahluwalia et al., 2013 | WIC receipt | “We estimated reliability by kappa coefficient and validity by sensitivity and specificity using the birth certificate data as the reference for the following: prenatal participation in the Special Supplemental Nutrition Program for Women, Infants, and Children (WIC); Medicaid payment for delivery; and breastfeeding initiation” |
| Baek, 2016 | Current Population Survey Food Security Supplement (CPS-FSS) | “I use the number of vehicles operated in urbanized areas (UA) from the National Transit Database (NTD) and household food insecurity data from the Current Population Survey Food Security Supplement (CPS-FSS) from 2006 to 2009” |
| Baer et al., 2015 | U.S. Household Food Security Survey Module | “To measure food insecurity, participants were administered the appropriate validated USDA-FSS based on age and parental status. The 18-item US Household Food Security Survey Module was answered by patients aged 18-25 years who self-identified as being a parent. The 10-item Adult Food Security Survey Module was completed by patients aged 18-25 years who did not self-identify as being a parent” |
| Balistreri, 2016 | Eighteen-Item Household Food Security Scale | “The household food security scale was developed by the USDA to measure the severity of food insecurity experienced in the household in the previous 12 months. It is measured with an 18-item scale if the household contains children and ten if it does not” |
| Barnidge et al., 2017 | Eighteen-Item Household Food Security Scale | “A two-item validated screener was used to assess household food security. The first item asked, ‘over the last 12 months did you worry there would not be enough food and there was no money to buy more?’ “Nearly one-fifth (17.5 %) of caregivers reported this was often true while 37.3 % reported this as sometimes true. Household food insecurity was measured using the first two questions from the 18-itemU.S. Household Food Security Survey” |
| Barnidge et al., 2017 | Eighteen-Item Household Food Security Scale | “Participants completed a demographic questionnaire that included age, household income, and participation in food assistance programs. The questionnaire also included the US Department of Agriculture’s 18-item Food Security Module to determine household food security status and the food security status of children in the home” |
| Boone-Heinonen et al., 2015 | Neighborhood Supermarket Density per 10,000 people | “Neighborhood food and physical activity amenities were obtained from Dun and Bradstreet, a commercial dataset of U.S. businesses (Dun & Bradstreet). Fast-food chain restaurants, supermarkets (large grocery stores), commercial physical activity facilities, and public physical activity facilities corresponding to each CARDIA exam period were extracted and classified according to 8-digit Standard Industrial Classification codes (U.S. Department of Labor)” |
| Borders et al., 2015 | U.S. Household Food Security Survey Module | “External stressors were measured using the Home Hardships Scale, the USDA Household Food Security Scale and the Neighborhood Satisfaction Scale” |
| Brewer et al., 2010 | Six-Item Short Form of the Food Security Survey Module | “Food insecurity was assessed using the modified 6-item US Household Food Security Survey Module” |
| Burke et al., 2018 | U.S. Household Food Security Survey Module | “Food security status was our dependent variable and was measured using the US Department of Agriculture (USDA) Household Food Security Survey Module (HFSSM) (Coleman-Jensen et al.2015). The HFSSM uses a 12-month recall period and 18 Likert-type items to assess the frequency in which household members report, because of a lack of resources, disruptions in the quality, quantity and patterns of the household food supply as well as anxiety related to running out of food” |
| Caraballo et al., 2020 | 10-item questionnaire recommended by the US Department of Agriculture Economic Research Service | “Food security in the past 30 days was created based on the 10-item questionnaire as recommended by the US Department of Agriculture Economic Research Service (Table I in the Data Supplement) 13,20 and constructed following the NHIS instructions.21 Answers of ≥3 days were considered affirmative in questions about frequency of occurrence in the past 30 days. A raw score ranging from 0 to 10 was calculated, and participants were categorized as follows: 0 to 2 points: food secure; 3 to 5 points: low food security; and 6 to 10 points: very low food security. We then defined food insecurity as having either low or very low food security, in concordance with previous studies” |
| Chakrabarti et al., 2021 | Food insufficiency | “Pandemic-related food insufficiency, defined as there sometimes or often not being enough food to eat in the last 7 days or food availability becoming worse after March 13, 2020” |
| Chilton and Booth, 2007 | Six-Item Short Form of the Food Security Survey Module | “Each participant, in both the focus groups and the individual interview sessions, also answered a brief questionnaire that included demographic characteristics, food stamp participation, and the US Department of Agriculture Household Food Security Scale—Short Form. Food Insecurity was calculated according to established methods. Terminology of food insecurity categories was recently changed by the US Department of Agriculture” |
| Clay and Ross, 2020 | 2-Item screen to identify families at risk for food insecurity | “The outcome food security was assessed with a validated two-item food security screener (97 percent sensitivity, 83 percent specificity) that was designed to rapidly identify individuals at-risk for food insecurity” |
| Conlon et al., 2015 | Six-Item Short Form of the Food Security Survey Module | “Household food security was assessed by parents’ self-response to six items from the Short Form of the Household Food Security Scale” |
| Cook et al., 2002 | U.S. Household Food Security Survey Module | “We used a somewhat more stringent method of scoring the Food Security Scale for this analysis than the standard US Department of Agriculture method, which led to conservative estimates of the effects of exposure on this outcome” |
| Cox and Wallace, 2016 | 18 food security questions in the CFSM | “There are 18 food security questions in the CFSM. The food security module is designed to allow administrators to implement two common screens (and a third less common screen) when it appears the food security questions may pose an unnecessary burden on the respondents. All three screens were used in the FFCWS survey” |
| Crabtree and Mushi-Brunt, 2013 | National Health Interview Survey on Disability (NHIS-D) with 10 questions about food access | “The NHIS-D includes questions about participation in several activities and functional limitations. We identified 10 questions about food access-related participation and functional limitations that likely affect being able to access food for nutrition” |
| Dean et al., 2011 | Radimer-Cornell hunger and food insecurity instrument | “Food insecurity was measured by the household-hunger dimension of the food-depletion item from the Radimer-Cornell hunger and food insecurity instrument” |
| Denney et al., 2020 | Eighteen-Item Household Food Security Scale | “Household food insecurity, a binary measure created from the USDA’s 18-item food insecurity scale (Bickel et al., 2000), serves as our outcome measure” |
| Duke et al., 2021 | 2-item Hunger Vital Sign Tool | “Food insecurity was assessed using the 2-item Hunger Vital Sign Tool. The baseline survey included the validated, two-item Hunger Vital Sign (HVS), a food insecurity screening tool based on the U.S. Household Food Security Survey Module” |
| Duke, 2021 | Radimer-Cornell hunger and food insecurity instrument | “Food insecurity was measured with the following question: ‘During the past 30 days, have you had to skip meals because your family did not have enough money to buy food?’ Students were able to respond ‘yes’ or ‘no’ to this question. Based on the Radimer/Cornell Hunger Scale,19 this single item focused on the experience of skipping meals represents child level or severe food insecurity, reflecting insufficiency of household food such that youth are affected by reductions in the quantity of food eaten” |
| Echeverria et al., 2004 | Access to healthy foods scale | “Scales were used to assess six neighborhood domains: aesthetic quality, walking/exercise environment, safety from crime, violence, access to healthy foods, and social cohesion” |
| Fleming et al., 2021 | NHANES Food Security Module | “NHANES has been using the Food Security Survey Module, similar to the module included in the Current Population Survey, to assess food security since 1999. This module is included in the family questionnaire portion of the NHANES household interview. An adult family member, typically the head of household, answers the family questionnaire on behalf of the entire family and questions refer to all household members. Households with children younger than 18 years of age receive an additional 8 questions for a total of 18 items, compared to households without children” |
| Garrett-Peters and Mills-Koonce, 2013 | Food insufficiency questions (5 items) | “Mothers completed food insufficiency questions (five items) about whether they or other household members went hungry or had to skip or cut meals because they could not afford to buy food in the last 12 months. Scores could range from 0 to 5” |
| Ghosh and Parish, 2015 | Not reported | “These included a measure of difficulty paying for utilities like electricity and or telephone bills; difficulty paying the full amount for rent or mortgage, difficulty meeting essential expenses and not going to a doctor and or a dentist when needed to any time in the last 12 months, and whether they experienced food insecurity any time in the last four months” |
| Gilbert and Ashley, 2020 | 4-item 7-point Likert scale adapted from food access questionnaire from Hendrickson, Smith, and Eikenberry (2006) | “We adapted food access questionnaire items from Hendrickson, Smith, and Eikenberry (2006), who investigated access to fruits and vegetables by low-income residents in urban and rural areas of Minnesota. We created a four-item, seven-point Likert scale in which respondents indicated the extent to which they agreed or disagreed with statements such as “I have access to a variety of foods” and “the fruits and vegetables I can access are fresh.” Each item is scored from 1 (“strongly disagree”) to 7 (“strongly agree”)” |
| Gilbert et al., 2017 | Participation in MAP + TANF + SNAP | “In addition to household income verification and documentation for WIC program eligibility, adjunctive eligibility measures, such as participation in other federal programs (i.e., medical assistance, food stamp (SNAP), and temporary cash assistance (TCA)) were gathered and electronically verified through linkage with respective agency databases prior to documentation and storage in the WOW database” |
| Hammer et al., 2021 | 3-item adaptation from the USDA Food Insecurity Scale | “Food insecurity was assessed based on responses to three food insecurity items derived from the USDA Household Food Security Survey Module.27 The items used asked how often, in the last 12 months, the respondent or people in the respondent’s household (1) worried whether your food would run out before you had money to buy more; (2) the food that you bought did not last, and you didn’t have enough money to get more; or (3) you couldn’t afford to eat balanced meals” |
| Harrison et al., 2005 | Six-Item Short Form of the Food Security Survey Module | “The food security measure used is an abbreviated six-item scale derived from the 18-item U.S. Household Food Security Instrument employed surveys and administered to CHIS respondents below 200% FPL” |
| Hernandez and Pressler, 2013 | Eighteen-Item Household Food Security Scale | “The measure of food insecurity is based on an 18-item scale developed by the U.S. Department of Agriculture that assesses both the quality and quantity of food over the past 12 months. The scale captures food hardship due to financial constraints such as running out of food, perceptions that food in a household is of inadequate quality or quantity, and reduced food intake by adults or children” |
| Huang et al., 2015 | Survey of Income and Program Participation. The SIPP | “The food insufficiency question in SIPP asked respondents to choose the best of the following statements describing household food experiences in each of 4 reference months:” |
| Kaiser et al., 2007 | Six-Item Short Form of the Food Security Survey Module | “Food insecurity of the women was measured by a 6-item subset of the Food Security Module” |
| Keene et al., 2015 | Survey of Income and Program Participation. The SIPP | “SIPP provides a nationally representative sample of rent-assisted households, to examine the association between housing-assistance type and reports that neighbors count on each other, watch each other’s children, help each other out, can trust each other to intervene in the face of danger or harm, and have access to help from friends to family” |
| Kharmats et al., 2014 | U.S. Adult Food Security Survey Module | “Food security over the past year was measured and scored by using the US Adult Food Security Survey Module (10 items). Participants were asked to tell the interviewer whether statements about food situations presented in the module were “often true, sometimes true, or never true for you/your household in the past 12 months” |
| Kipke et al., 2020 | Not reported | “Food security (last 12 months)” |
| Koh et al., 2020 | Six-Item Short Form of the Food Security Survey Module | “Using the Six-Item Household Food Security Survey Module designed by USDA, the FMTS elicited responses to five questions and statements about respondents’ food procurement experience in the last 12 months” |
| Laraia et al., 2006 | Eighteen-Item Household Food Security Scale | “Food security—main outcome. “The USDA food security modules comprised of 18 questions posed in increasing levels of severity by measuring the dimensions of concern about food quantity and food quality over the last 12 months” |
| Laraia et al., 2009 | Six-Item Short Form of the Food Security Survey Module | “To calculate household food insecurity status, the six-item short form of the USDA Core Food Security Module (CFSM) for families was used (29,30). Questions were asked about the household’s experience over the past 12 months” |
| Lauren et al., 2021 | 2-Item screen to identify families at risk for food insecurity | “We assessed household risk for food insecurity using a validated two-item screen. (16) Households at risk for food insecurity were defined as those with responses of “Sometimes true” or “Often true” for either or both items. Participants reported answers to each of these questions for the periods before and after the COVID-19 outbreak” |
| Lee et al., 2011 | Six-Item Short Form of the Food Security Survey Module | “Scoring for the modified 6-item HFSSM was completed to reflect the validated survey module. For the identical questions from the original HFSSM, responses of ‘‘often’’ or ‘‘sometimes’’ on questions ‘‘food didn’t last’ ’and ‘‘couldn’t afford balanced meals’’ and ‘‘yes’’ on ‘‘ate less’’ and ‘‘hungry’ ’were coded as affirmative” |
| Leigh and Medal-Herrero, 2015 | WIC receipt | “Our dependent variable, was binary and equaled 1 if the subject or anyone in the household received WIC benefits in the preceding 2 years” |
| Lise et al., 2021 | Eighteen-Item Household Food Security Scale | “Household food security was assessed using the 18-item USDA US-Household Food Security Survey Module (HFSSM), which queries the past 12 months” |
| Lin et al., 2021 | Food Insecurity Experience Scale (FIES) | “We also included the Food and Agriculture Organization’s Food Insecurity Experience Scale (FIES), which captures respondent’s reporting of any food deprivation (e.g., constraints on one’s ability to obtain adequate food) both prior to and during the pandemic” |
| Lombe et al., 2009 | Eighteen-Item Household Food Security Scale | “Household Food Security—the dependent variable—is measured using items taken from the USDA 18-item Core Food Security Module (CFSM), a self-report measure of household food security for the past 12 months” |
| Long et al., 2020 | U.S. Household Food Security Survey Module | “Household food security status was assessed in the NHIS and NHPI-NHIS via the 10-item US Adult Food Security Survey Module (Table 1).54 All respondents were asked the 10-item module, as opposed to the full battery of 18 items that are asked of households with children in the CPS” |
| Makelarski et al., 2015 | 2-item screen derived from the 18-item US Household Food Security Screen | “We measured household food insecurity in the past 12 months by using a validated 2-item screen derived from the 18-item US Household Food Security Screen (affirmative responses to either survey item indicated a positive screen for household food insecurity” |
| Martin et al., 2004 | U.S. Household Food Security Survey Module | “Household food security and hunger were measured using the US Household Food Security Module” |
| Martin et al., 2016 | Eighteen-Item Household Food Security Scale | “Food security was measured using the USDA 18-item Food Security Module” |
| McCurdy and Metallinos-Katsaras, 2011 | 4 Items from the 18-Item Food Security Core Module (FSCM) | “Household food security status was assessed with caregiver Reponses to 4 items from the 18-item Food Security Core Module (FSCM), commonly used to measure food security status in the United States.24Caregiverswere asked about the following for the previous 12-month time period: (1) not having enough money to buy food for a balanced meal, (2) adults cutting the size of meals or skipping meals, (3) frequency of cutting or skipping meals, and (4) adults not eating for a whole day” |
| McDonough et al., 2019 | 18-item Food Security Core Module (FSCM) | “Using definitions of food security provided by the USDA, we assign each household to a particular state of food security where the various states of food security are food insecure, marginal food secure, and high food secure (i.e., K = 3). We then track how households move through the distribution of food security from one period to the next. Additionally, the food security questions surveyed come from the USDA’s Core Food Security Module” |
| Miller et al., 1996 | Nutrition Screening Initiative (NSI)& 10-item DETERMINE Checklist | “Nutritional risk was measured using the Nutrition Screening Initiative Checklist. Demographic information, economic status, self-rated health, Geriatric Depression Scale score, and body mass index were assessed using established standardized instruments” |
| Miller et al., 2015 | Number of full-service retail food outlets (RFOs) in the neighborhood | “Food stores within one’s census tract should be fairly accessible with or without automobile access or other transportation arrangements. In this article we simply measure food availability, or the presence of full service RFOs that offer a selection of fresh or frozen fruits and vegetables” |
| Mook et al., 2016 | Six-Item Short Form of the Food Security Survey Module | “Data on food security status were collected by using the US Department of Agriculture’s 6-item short-form food security scale (21). Food security status was categorized as food secure (raw score 0–1) or food insecure (raw score 2–6) and analyzed as a dichotomous variable (21)” |
| Moore et al., 2020 | Six-Item Short Form of the Food Security Survey Module | “The food security survey utilized questions from a validated survey, the US Household Food Security Survey Module: Six-Item Short Form developed by the United States Department of Agriculture (USDA).17 The sum of affirmative responses to six questions was used to calculate a raw score that determined the level of food security as food secure (score 0–1 = high security or marginal insecurity), low food security (score 2–4), or very low food security (score 5–6)” |
| Morales et al., 2020 | Household Pulse Survey (HPS) food insecurity measure | “Instead of including any items from the HFSSM scale, the HPS collected a different measure of food insecurity by asking respondents to choose a statement that best described the food eaten situation in their households over the past 7 days [42]. A recent research report confirmed that the HPS measure is a good indicator of household food insecurity and aligns with the HFSSM measure [43]. We used responses to the HPS question to construct a variable measuring the level of household food insecurity during the COVID-19 pandemic, which ranges from 0 = food secure (“enough of the kinds of food I/ we wanted to eat”) to 3 = severely food insecure (“often not enough to eat”)” |
| Murimi et al., 2016 | 18 food security questions in the CFSM | “The Spanish translation of the US Household Food Security Survey Module by USDA researchers was used. Standard 12 months Core Food Security Module (CFSM) questions were used with the assumption that data collected would capture events of the past 12 months” |
| Myers and Painter, 2017 | NHANES Food Security Module | “The NHANES food security module includes questions on participants’ food situations at home and food assistance benefits (i.e., those associated with SNAP) received within the previous 12 months” |
| Myers et al., 2020 | U.S. Household Food Security Survey Module | “Food security status was measured through the US Household Food Security Survey Module (developed by the US Department of Agriculture), which comprises 18 items that assess the “conditions and behaviors that characterize … difficulty meeting basic food needs” Three or more affirmative responses in this module indicate food insecurity” |
| Nagata et al., 2021 | U.S. Household Food Security Survey Module | “The questions regarding food sufficiency were consistent with the U.S. Household Food Security Survey Module. Food insufficiency generally describes whether households have enough food for their families to eat and is often the most extreme form of food insecurity. In other surveys, such as the Current Population Survey’s Food Security Supplement, 86%‒89% of people reporting food insufficiency were deemed food insecure” |
| Nam et al., 2014 | Food Insufficiency Indicator (from SEED OK survey) | “The dependent variable in this study is a food insufficiency indicator created with a question in SEED OK’s baseline survey: “During the past 12 months, did your family (1) always have enough to eat, (2) sometimes not have enough to eat, or (3) of-ten not have enough to eat? “This item is a slightly modified version of the first screening question in the U.S. Department of Agriculture’s Food Security Core-Module Questionnaire” |
| Okafor et al., 2020 | 2-item Hunger Vital Sign Tool | “From January 2017 through February 2017, we utilized a validated two-item screening tool to assess the prevalence of households at risk for food insecurity and conducted a focus group of pediatricians. The USDA provides several tools (6-, 10-, and 18-item) to screen for food insecurity (USDA ERS: Survey Tools, 2019). In November 2015, the American Academy of Pediatrics (AAP) issued a policy statement (Promoting Food Security for All Children, 2018) that endorses the use of the two-item screening tool, now coined the Hunger Vital Sign” |
| O’Reilly et al., 2020 | 2-Item screen to identify families at risk for food insecurity | “Household food insecurity risk was measured with a 2-item screen [36]: (a) “within the past 12 months, we worried whether our food would run out before we got money to buy more” and (b) “within the past 12 months, the food we bought just didn’t last and we didn’t have money to get more.” Answering affirmatively to either or both questions indicate household food insecurity risk, coded 0 = food secure; 1 = food insecure risk” |
| Orozoco et al., 2020 | U.S. Household Food Security Survey Module | “A validated US Department of Agriculture 18-item Food Security Survey Module was used in the NHANES 2009−2014 surveys to assess household food security status over the prior 12 months. The scale consisted of statements representing coping behaviors or experiences owing to insufficient money to buy food, leading to a score of 0−18” |
| Palmer et al., 2018 | Six-Item Short Form of the Food Security Survey Module | “Food security was measured according to the six-item USDA core food security module” |
| Palmer et al., 2020 | Six-Item Short Form of the Food Security Survey Module | “The 6-item USDA Core Food Security Module (CFSM) estimated food security” |
| Paschal et al., 2020 | U.S. Adult Food Security Survey Module | “The 10-item U.S. Adult Food Security Survey Module40 was used to assess food insecurity. In using the 10-item module, direct inquiry about children’s food insecurity was avoided, which was a sensitive issue and concern for one of the partners. Survey burden was also lowered with this version compared to the 18-item module” |
| Patterson et al., 2020 | U.S. Household Food Security Survey Module | “Both NHIS and NHANES measure food security using the USDA US Food Security Survey Module (α = 0.74–0.93) (Keenan et al., 2001). The survey module asks adults to report their experiences with food security using a scale of 0–10. Levels of food security are designed as “full food security” (0 points), “marginal food security” (1–2 points), “low food security” (3–5 points), and “very low food security” (6–10 points)” |
| Payne-Sturges et al., 2018 | U.S. Household Food Security Survey Module | “Food security status was assessed via the USDA 18-item Household Food Security Survey Module (HFSSM)” |
| Perry et al., 2020 | Not reported | “Key outcome markers include four subjective and objective markers of financial strain. The first three measure respondents’ reported level of housing insecurity, food insecurity, and general financial insecurity attributed to the COVID-19 pandemic. Housing insecurity, food insecurity, and general financial insecurity were measured in wave 2 by asking respondents the extent to which they agreed that COVID-19 has made them worry that they “may not have a place to live,” that they “may not have enough money to buy food,” and “about their finances, in general” (0 = strongly disagree, 1 = disagree, 2 = agree, and 3 = strongly agree)” |
| Pooler and Gleason, 2014 | WIC receipt | “Dependent variables of interest in this study included the full use of WIC benefits overall and for each of the 14 WIC food categories. To determine whether benefits were used fully, the same method was applied to each of the 14 food categories:” |
| Rank and Hirsch, 2009 | Receipt of food stamps in the past 12 months | “Food stamp use was derived from a series of questions asked by the PSID interviewers as to whether the household had received specific cash or in-kind public assistance programs at some point during the prior year. With regard to food stamps, respondents were asked, “Did you (or anyone else in your family) use government food stamps at any time in [prior year]?” |
| Reeder et al., 2020 | Six-Item Short Form of the Food Security Survey Module | Food security status was measured using the United States Department of Agriculture’s U.S. Household Food Security Survey Module: Six-Item Short Form [26]. This survey uses a subset of questions from the standard 18-item Food Security Survey Module. The Six-Item Short Form is a reliable substitute for the 18-item Food Security Survey Module and has a lower respondent burden. |
| Ruprecht et al., 2020 | Not reported | “Food shortage” |
| Harare et al., 2020 | Not reported | “Drawing on the food insecurity literature 12−15 a number of potential community-level sociodemographic variables were reviewed that may differentiate the two-by-two typology” |
| Sharkey and Schoenberg, 2002 | Nutrition Screening Initiative (NSI)& 10-item DETERMINE Checklist | “Nutritional risk was assessed by administering the Nutrition Screening Initiative (NSI)’s 10-item DETERMINE Checklist. The Checklist was developed as a self-report preliminary screen for warning signs of increased risk for poor nutritional health and is used by the ENP in most states for nutrition risk screening” |
| Sharkey and Schoenberg, 2005 | Food Sufficiency Status based on four self-reported risk situations that were related to absence of food and forced scarce-resource decisions | “The level of food sufficiency, during the 6months prior to the in-home assessment, was operationalized from four self-reported risk situations that were related to absence of food and forced scarce-resource decisions and that were previously used in national evaluation of OAANP to characterize the difficulty in meeting basic food needs among homebound meal program participants” |
| Siddiqi et al., 2021 | Six-Item Short Form of the Food Security Survey Module | “We assessed food security in 2018 and 2020 using the validated Adult Food Security Survey Module Six-Item Short Form.21 We created a 3-level food security measure based on the survey module scores: we categorized people with scores ranging from 0 to 1.0 as being food secure, people with scores ranging from 2.0 to 4.0 as having a low level of food security, and people with scores ranging from 5.0 to 6.0 as having a very low level of food security. We based scores on the number of affirmative responses to the 6 questions included in the survey module” |
| Stallings et al., 2016 | Enrollment in Farmer’s Market Nutrition Plan (FMNP) | “In addition to food vouchers and nutrition education, most states’ WIC programs offer the Farmers’ Market Nutrition Program (FMNP), which provides FMNP coupons to be redeemed at a WIC-approved farmers’ market as a means to increase fresh F&V consumption” |
| Stewart et al., 2011 | 2007 AIDS Alabama Needs Assessment Survey | “The interview covered demographics; basic needs such as food, transportation, and housing; and a wide range of other ancillary services. A total of 14 basic and ancillary service needs were included: financial, legal, pharmaceutical, employment, and housing assistance; substance use, mental health, and alcohol treatment; medical services, transportation, dentalcare, case management, food, and childcare” |
| Stockman et al., 2020 | WHO survey tool on COVID stressors | “We assessed nine COVID-19 stressors as outlined by the WHO [20]. Examples include food insecurity, insufficient rent, and caregiver status. We employed the Fear of COVID-19 Scale, a 7- item scale, self-reported measure of an individual’s fear of COVID-19” |
| Stuff et al., 2004 | Eighteen-Item Household Food Security Scale | “This interview included the 18-question US Food Security Survey Module and had questions about participation in nutrition assistance programs and income” |
| Tackett et al., 2018 | Eighteen-Item Household Food Security Scale | “National Health and Nutrition Examination Survey—Household Food Security Questionnaire. This 18-item questionnaire characterizes household food security status. Items assessed food access problems, limitations, food sufficiency, or food shortage” |
| Tamar goes et al., 2021 | U.S. Household Food Security Survey Module | “FI was determined with the Household Food Security Module, which assesses a respondent’s perceived food sufficiency and adequacy during the past 12 months” |
| Tan et al., 2020 | U.S. Household Food Security Survey Module | “Food insecurity, which were assessed using the Household Food Security Survey (HFSS) module, a validated scale considered the reference measure of food security in the United States (22, 23). This 18-item scale captures uncertainty about food supply, sufficiency of food quantity, and diet quality over the previous 12 months” |
| Tong et al., 2019 | Six-Item Short Form of the Food Security Survey Module | “We assessed food security using the Six-Item Short Form of the US Household Food Security Survey Module (FSSM), a validated measure of food security in the general population and among older adults” |
| Trego et al., 2019 | U.S. Adult Food Security Survey Module | “Food insecurity, the dependent variable, was measured by the 10-item adult US Food Security Survey Module (FSSM). The FSSM is designed to assess household financial ability to meet food needs within the past 12 months using Likert scale and yes/no questions” |
| Tucker-Seeley et al., 2016 | Health and Retirement Study (HRS) Food Insecurity question | “The items from the HRS were selected across five domains of hardship based on the poverty and life stress literatures as well as factors revealed in previous investigations of the measurement properties of hardship indicators: financial hardship, food hardship, housing/neighborhood hardship, employment instability, and medical need” |
| Vedovato et al., 2016 | Eighteen-Item Household Food Security Scale | “Food security was assessed using the US Department of Agriculture’s eighteen-item Household Food Security Scale for households with children under 18 years old. Household food security was determined by the number of food-insecure conditions and reported behaviors reported in the past 12 months” |
| Walker et al., 2020 | U.S. Household Food Security Survey Module | “During each year of the survey, the same validated scale for food insecurity was used. Ten questions were asked of each family using questions and response options from the US Department of Agriculture Food Security Survey Module. A 4-level food security status was calculated based on the number of affirmative responses using scoring from Bickel et al” |
| Wang et al., 2015 | First item of the Household Food Insecurity Access Scale | “To capture the broadest domain of food insecurity—uncertainty about food access— VACS incorporated the first question of the 18-item Household Food Insecurity Access Scale (HFIAS) in the baseline patient survey” |
| Wang et al., 2021 | Six-Item Short Form of the Food Security Survey Module | “Our primary outcome was food security. We scored the 6-item version of the US Department of Agriculture’s US Household Food Security Survey Module: Six-Item Short Form as a dichotomous variable: food secure (0 or 1 item answered affirmatively) or food insecure (2–6 items answered affirmatively)” |
| Weaver et al., 2019 | U.S. Household Food Security Survey Module | “The questionnaire used the United States Department of Agriculture (USDA) food security measure to assess food security. Over time, the measure has been modified and expanded and, most recently, includes a 10-item index” |
| Whitbeck et al., 2006 | 3-item adaptation from the USDA Food Insecurity Scale | “We assessed food insecurity with three items adapted from the USDA food insecurity scale that dealt specifically with going without or cutting back food. It should be noted that these are only three items from a 16-item scale, and they do not reflect the nuances of the full USDA measure” |
| Wilson et al., 2006 | U.S. Household Food Security Survey Module | “The 16-item US FSSM was verbally administered to each HD participant. Respondents answered often true, sometimes true, or never true to each item” |
| Wolfe et al., 1996 | Not reported | Not reported |
| Wood and Harris, 2018 | Not reported | “Food insecurity was a dichotomous measure based on students indicating challenges with “hunger,” an acute form of food insecurity” |
| Yu et al., 2010 | Eighteen-Item Household Food Security Scale | “Child Food Security (the dependent variable) and Household Food Security (independent variable) are measured using items taken from the USDA 18-item Core Food Security Module (CFSM). Nine items from the CFSM use household food security. In the past12 months, households were asked whether: 1) they worried their food would run out before they got money to buy food; 2) the food that they bought just didn’t last and they didn’t have money to get more; 3) they couldn’t afford to eat balanced meals; 4) they relied on only on a few kinds of low-cost food to feed their children because they were running out of money to buy food; 5) they ever cut the size of their meals or skip meals because there wasn’t not enough money for food; 6) they ever ate less than they felt they should because there wasn’t enough money to for food; 7) they were ever hungry but didn’t eat because they couldn’t afford enough food; 8) they lost weight because they didn’t have enough money for food; and 9) they ever not eat for a whole day because there wasn’t enough money for food” |

Table S5. Citation characteristics (sorted by state, region, and study design) of studies included in a scoping review of food insecurity in African American adults

| **Citation** | **State(s)** | **Region** | **Study Design** |
| --- | --- | --- | --- |
| Ahluwalia et al., 2013 | Not Reported | Not Reported | Cross-sectional |
| Baek, 2016 | Not Reported | Urban | Cross-sectional |
| Baer et al., 2015 | Massachusetts | Urban | Cross-sectional |
| Balistreri, 2016 | Not Reported | Not Reported | Cohort (longitudinal) |
| Barnidge et al., 2017 | Missouri | Not Reported | Cross-sectional |
| Barnidge et al., 2017 | Missouri | Both (urban and rural) | Concept mapping |
| Boone-Heinonen et al., 2015 | Minnesota, California, Alabama, and Illinois | Not Reported | Cohort (longitudinal) |
| Borders et al., 2015 | Illinois | Urban | Cohort (longitudinal) |
| Brewer et al., 2010 | Georgia | Not Reported | Cross-sectional |
| Burke et al., 2018 | South Carolina | Both (urban and rural) | Cross-sectional |
| Caraballo et al., 2020 | Not Reported | Not Reported | Cross-sectional |
| Chakrabarti et al., 2021 | All 50 US states + DC | Not Reported | Cross-sectional |
| Chilton and Booth, 2007 | Pennsylvania | Not Reported | Qualitative |
| Clay and Ross, 2020 | Texas | Both | Cross-sectional |
| Conlon et al., 2015 | New York | Urban | Randomized controlled trial |
| Cook et al., 2002 | Minnesota, Maryland, California, Massachusetts, Arkansas, and Washington D.C. | Urban | Cohort (longitudinal) |
| Cox and Wallace, 2016 | Not Reported | Not Reported | Cohort (longitudinal) |
| Crabtree and Mushi-Brunt, 2013 | Not Reported | Both (urban and rural) | Cross-sectional |
| Dean et al., 2011 | Texas | Rural | Cross-sectional |
| Denney et al., 2020 | Not Reported | Not Reported | Cross-sectional |
| Duke et al., 2021 | North Carolina | Urban | Cross-sectional |
| Duke, 2021 | Minnesota | Not Reported | Cross-sectional |
| Echeverria et al., 2004 | New York | Urban | Cross-sectional |
| Fleming et al., 2021 | Not Reported | Not Reported | Cross-sectional |
| Garrett-Peters and Mills-Koonce, 2013 | North Carolina and Pennsylvania | Rural | Cross-sectional |
| Ghosh and Parish, 2015 | Not Reported | Not Reported | Cohort (longitudinal) |
| Gilbert and Ashley, 2020 | Not Reported | Urban | Cross-sectional |
| Gilbert et al., 2017 | Maryland | Not Reported | Cross-sectional |
| Hanmer et al., 2021 | Not Reported | Not Reported | Cross-sectional |
| Harrison et al., 2005 | California | Not Reported | Cross-sectional |
| Hernandez and Pressler, 2013 | Not Reported | Not Reported | Cohort (longitudinal) |
| Huang et al., 2015 | Not Reported | Not Reported | Cohort (longitudinal) |
| Kaiser et al., 2007 | California | Not Reported | Cross-sectional |
| Keene et al., 2015 | Not Reported | Urban | Cross-sectional |
| Kharmats et al., 2014 | Maryland | Urban | Cross-sectional |
| Kipke et al., 2020 | California | Urban | Cross-sectional |
| Koh et al., 2020 | Ohio | Urban | Cross-sectional |
| Laraia et al., 2006 | North Carolina | Not Reported | Cross-sectional |
| Laraia et al., 2009 | North Carolina | Not Reported | Cross-sectional |
| Lauren et al., 2021 | Not Reported | Not Reported | Cross-sectional |
| Lee et al., 2011 | Georgia | Both (urban and rural) | Cohort (longitudinal) |
| Leigh and Medel-Herrero, 2015 | California | Not Reported | Cross-sectional |
| Liese et al., 2021 | South Carolina | Urban | Cohort (longitudinal) |
| Lin et al., 2021 | Not Reported | Not Reported | Cross-sectional |
| Lombe et al., 2009 | Not Reported | Not Reported | Cross-sectional |
| Long et al., 2020 | Not Reported | Not Reported | Cross-sectional |
| Makelarski et al., 2015 | Illinois | Urban | Cross-sectional |
| Martin et al., 2004 | Connecticut | Urban | Cross-sectional |
| Martin et al., 2016 | Connecticut | Urban | Randomized controlled trial |
| McCurdy and Metallinos-Katsaras, 2011 | Massachusetts | Not Reported | Cohort (longitudinal) |
| McDonough et al., 2019 | Not Reported | Not Reported | Cohort (longitudinal) |
| Miller et al., 1996 | Missouri and Illinois | Urban | Cross-sectional |
| Miller et al., 2015 | Kansas | Urban | Cross-sectional |
| Mook et al., 2016 | California | Urban | Cross-sectional |
| Moore et al., 2020 | Texas | Urban | Cross-sectional |
| Morales et al., 2020 | All 50 US states + DC | Not Reported | Cross-sectional |
| Murimi et al., 2016 | Texas | Both (urban and rural) | Cross-sectional |
| Myers and Painter, 2017 | Not Reported | Not Reported | Cross-sectional |
| Myers et al., 2020 | Not Reported | Not Reported | Cross-sectional |
| Nagata et al., 2021 | Not Reported | Not Reported | Cross-sectional |
| Nam et al., 2014 | Oklahoma | Not Reported | Cross-sectional |
| Okafor et al., 2020 | Connecticut | Not Reported | Cross-sectional |
| O'Reilly et al., 2020 | Not Reported | Urban | Cross-sectional |
| Orozoco et al., 2020 | Not Reported | Not Reported | Cross-sectional |
| Palmer et al., 2018 | Iowa | Both (urban and rural) | Cross-sectional |
| Palmer et al., 2020 | Iowa | Not Reported | Cross-sectional |
| Paschal et al., 2020 | Alabama | Both (urban and rural) | Cross-sectional |
| Patterson et al., 2020 | Not Reported | Not Reported | Cross-sectional |
| Payne-Sturges et al., 2018 | Not Reported | Not Reported | Cross-sectional |
| Perry et al., 2020 | Indiana | Both (urban and rural) | Cohort (longitudinal) |
| Pooler and Gleason, 2014 | Michigan | Both (urban and rural) | Cross-sectional |
| Rank and Hirschl, 2009 | Not Reported | Not Reported | Cohort (longitudinal) |
| Reeder et al., 2020 | Mississippi | Not Reported | Cross-sectional |
| Ruprecht et al., 2020 | Illinois | Urban | Cross-sectional |
| Sharareh et al., 2020 | Utah | Not Reported | Cross-sectional |
| Sharkey and Schoenberg, 2002 | North Carolina | Not Reported | Cross-sectional |
| Sharkey and Schoenberg, 2005 | North Carolina | Not Reported | Cross-sectional |
| Siddiqi et al., 2021 | Pennsylvania | Urban | Cohort (longitudinal) |
| Stallings et al., 2016 | Georgia | Urban | Randomized controlled trial |
| Stewart et al., 2011 | Alabama | Rural | Cross-sectional |
| Stockman et al., 2020 | Not Reported | Both (urban and rural) | Cross-sectional |
| Stuff et al., 2004 | Louisiana, Mississippi, and Arkansas | Not Reported | Cross-sectional |
| Tackett et al., 2018 | Not Reported | Not Reported | Cross-sectional |
| Tamargo et al., 2021 | Florida | Urban | Cohort (longitudinal) |
| Tan et al., 2020 | Not Reported | Not Reported | Cross-sectional |
| Tong et al., 2019 | California | Not Reported | Cross-sectional |
| Trego et al., 2019 | Not Reported | Not Reported | Cross-sectional |
| Tucker-Seeley et al., 2016 | Not Reported | Not Reported | Cohort (longitudinal) |
| Vedovato et al., 2016 | Maryland | Urban | Cross-sectional |
| Walker et al., 2020 | Not Reported | Not Reported | Cross-sectional |
| Wang et al., 2015 | Not Reported | Not Reported | Cohort (longitudinal) |
| Wang et al., 2021 | California | Urban | Cohort (longitudinal) |
| Weaver et al., 2019 | New Jersey | Not Reported | Cross-sectional |
| Whitbeck et al., 2006 | Kansas, Missouri, Iowa, and Nebraska | Urban | Cross-sectional |
| Wilson et al., 2006 | Louisiana | Not Reported | Cross-sectional |
| Wolfe et al., 1996 | New York | Both (urban and rural) | Qualitative |
| Wood and Harris, 2018 | California | Not Reported | Cross-sectional |
| Yu et al., 2010 | Not Reported | Not Reported | Cross-sectional |

Table S6. Spread of study participants’ ages (sorted by lower and upper age limits, number of study participants, and number of households) in included studies in a scoping review of food insecurity in African American adults

| **Citation** | **Lower age limit** | **Upper age limit** | **Central Tendency** | **Total African Americans** | **Total Participants** | **Total African American households** | **Total households** |
| --- | --- | --- | --- | --- | --- | --- | --- |
| Ahluwalia et al., 2013 | Not reported | Not reported | Not reported | 2384 | 14478 | 2384 | 14478 |
| Baek, 2016 | Not reported | Not reported | Not reported | Not reported | 28304 | Not reported | 28304 |
| Baer et al., 2015 | 15 | 25 | Mean: 18 | 216 | 400 | 216 | 400 |
| Balistreri, 2016 | Not reported | Not reported | Not reported | Not reported | 263,779 | Not reported | 263,779 |
| Barnidge et al., 2017 | Not reported | Not reported | Median: 31 | 160 | 212 | 160 | 212 |
| Barnidge et al., 2017 | Not reported | Not reported | Median: 37 | 38 | 38 | 38 | 38 |
| Boone-Heinonen et al., 2015 | 18 | 30 | Not reported | 2038 | 4174 | 2038 | 4174 |
| Borders et al., 2015 | Not reported | Not reported | Not reported | 55 | 112 | 55 | 112 |
| Brewer et al., 2010 | Not reported | Not reported | Median: 76 | 220 | 621 | 220 | 621 |
| Burke et al., 2018 | Not reported | Not reported | Mean: 40.8 | 154 | 194 | 154 | 194 |
| Caraballo et al., 2020 | 18 | 64 | Mean: 51.6 | 1781 | 8967 | Not-reported | Not-reported |
| Chakrabarti et al., 2021 | Not-reported | Not-reported | Mean: 51.55 | 86062 | 1088314 | Not-reported | Not-reported |
| Chilton and Booth, 2007 | 25 | 60 | Mean: 45 | 34 | 34 | 34 | 34 |
| Clay and Ross, 2020 | 18 | 64 | Not-reported | 101 | 1002 | 101 | 1002 |
| Conlon et al., 2015 | 22 | 67 | Mean: 37.1 | 54 | 301 | 54 | 301 |
| Cook et al., 2002 | Not reported | Not reported | Not reported | 1872 | 2178 | 1872 | 2178 |
| Cox and Wallace, 2016 | Not reported | Not reported | Not reported | Not reported | 4898 | Not reported | Not reported |
| Crabtree and Mushi-Brunt, 2013 | 18 | 99 | Mean: 63.92 | 1140 | 4672 | 1140 | 4672 |
| Dean et al., 2011 | Not reported | Not reported | Mean: 64.1 | Not reported | 1059 | Not reported | 1059 |
| Denney et al., 2020 | Not-reported | Not-reported | Mean: 33.49 | 1290 | 8600 | 1290 | 8600 |
| Duke et al., 2021 | 18 | 48 | mean: 20.1 | 320 | 351 | Not-reported | Not-reported |
| Duke, 2021 | 18 | 19 | Mean: 14.8 | Not-reported | 644 | Not-reported | Not-reported |
| Echeverria et al., 2004 | Not reported | Not reported | Mean: 38.4 | Not reported | 48 | Not reported | 48 |
| Fleming et al., 2021 | 13 | 18 | Median: 15 | 1207 | 4777 | Not-reported | Not-reported |
| Garrett-Peters and Mills-Koonce, 2013 | Not reported | Not reported | Not reported | Not reported | Not reported | Not reported | Not reported |
| Ghosh and Parish, 2015 | Not reported | Not reported | Not reported | 2049 | 9919 | 2049 | 9919 |
| Gilbert and Ashley, 2020 | Not-reported | Not-reported | Mean: 47.6 | 365 | 498 | 365 | 498 |
| Gilbert et al., 2017 | Not reported | Not reported | Mean: 26.8 | 8928 | 23065 | 8928 | 23065 |
| Hanmer et al., 2021 | 18 | 75 | Not-reported | 467 | 4142 | Not-reported | Not-reported |
| Harrison et al., 2005 | 18 | Not reported | Not reported | Not reported | 2926000 | Not reported | Not reported |
| Hernandez and Pressler, 2013 | Not reported | Not reported | Mean: 28.8 | 396 | 1650 | 396 | 1650 |
| Huang et al., 2015 | Not reported | Not reported | Mean: 39.94 | 4120 | 18263 | 4120 | 18263 |
| Kaiser et al., 2007 | 18 | 98 | Not reported | 252 | 4037 | 252 | 4037 |
| Keene et al., 2015 | Not reported | Not reported | Not reported | 905 | 905 | 905 | 905 |
| Kharmats et al., 2014 | 22 | 89 | Mean: 47.6 | 244 | 362 | 244 | 362 |
| Kipke et al., 2020 | 16 | 24 | Mean: 22.3 | 94 | 448 | Not-reported | Not-reported |
| Koh et al., 2020 | 18 | 65 | Not-reported | 148 | 586 | 148 | 586 |
| Laraia et al., 2006 | 16 | 45 | Mean: 27.2 | 201 | 606 | 201 | 606 |
| Laraia et al., 2009 | 18 | 35 | Not reported | 206 | 206 | 206 | 206 |
| Lauren et al., 2021 | 18 | 65 | Not-reported | 73 | 1250 | 73 | 1250 |
| Lee et al., 2011 | Not reported | Not reported | Mean: 74.6 | 238 | 717 | 238 | 717 |
| Leigh and Medel-Herrero, 2015 | Not reported | Not reported | Not reported | Not reported | 40,896 | Not reported | 40,896 |
| Liese et al., 2021 | Not-reported | Not-reported | Mean: 54.1 | 373 | 397 | 373 | 397 |
| Lin et al., 2021 | 18 | 49 | Not-reported | 39 | 554 | 39 | 554 |
| Lombe et al., 2009 | 18 | 80 | Mean: 45.2 | 3104 | 23360 | 3104 | 23360 |
| Long et al., 2020 | 18 | not-reported | Not-reported | 5056 | 38860 | 5056 | 38860 |
| Makelarski et al., 2015 | Not reported | Not reported | Not reported | 87 | 200 | 87 | 200 |
| Martin et al., 2004 | Not reported | Not reported | Not reported | 145 | 330 | 145 | 330 |
| Martin et al., 2016 | Not reported | Not reported | Mean: 51.4 | 164 | 227 | 164 | 227 |
| McCurdy and Metallinos-Katsaras, 2011 | Not reported | Not reported | Not reported | 3049 | 18039 | 3049 | 18039 |
| McDonough et al., 2019 | Not-reported | not-reported | Not-reported | 642 | 6822 | 642 | 6822 |
| Miller et al., 1996 | Not reported | Not reported | Not reported | 416 | 416 | 416 | 416 |
| Miller et al., 2015 | Not reported | Not reported | Not reported | Not reported | 177,688 | Not reported | Not reported |
| Mook et al., 2016 | Not reported | Not reported | Not reported | 272 | 531 | 272 | 531 |
| Moore et al., 2020 | 18 | not-reported | Not-reported | 90 | 602 | 90 | 602 |
| Morales et al., 2020 | Not-reported | Not-reported | Mean: 48.236 | 8546 | 74413 | 8546 | 74413 |
| Murimi et al., 2016 | Not reported | Not reported | Not reported | 62 | 191 | 62 | 191 |
| Myers and Painter, 2017 | Not reported | Not reported | Mean: 46.21 | 5762 | 32464 | 5762 | 32464 |
| Myers et al., 2020 | 20 | Not-reported | Mean: 46.9 | 5389 | 46145 | 5389 | 46145 |
| Nagata et al., 2021 | Not-reported | Not-reported | mean: 48.46 | 6985 | 63674 | 6985 | 63674 |
| Nam et al., 2014 | Not reported | Not reported | Not reported | 465 | 2652 | 465 | 2652 |
| Okafor et al., 2020 | 1.08 | 94 | Not-reported | 388 | 1299 | 388 | 1299 |
| O'Reilly et al., 2020 | Not-reported | Not-reported | Not-reported | 409 | 450 | 409 | 450 |
| Orozoco et al., 2020 | Not-reported | Not-reported | Not-reported | 417 | 2069 | 417 | 2069 |
| Palmer et al., 2018 | Not reported | Not reported | Mean: 34.7 | 14 | 36 | 14 | 36 |
| Palmer et al., 2020 | 19 | 50 | Mean: 34.7 | 14 | 36 | 14 | 36 |
| Paschal et al., 2020 | 45 | 65 | Not-reported | 102 | 102 | 102 | 102 |
| Patterson et al., 2020 | 18 | 59 | Not-reported | 11211 | 52702 | 11211 | 52702 |
| Payne-Sturges et al., 2018 | Not reported | Not reported | Mean: 20.69 | 20 | 237 | 20 | 237 |
| Perry et al., 2020 | Not-reported | Not-reported | Mean: 45.77 | 89 | 994 | 89 | 994 |
| Pooler and Gleason, 2014 | Not reported | Not reported | Not reported | 40230 | 152794 | 40230 | 152794 |
| Rank and Hirschl, 2009 | 1 | 20 | Not reported | Not reported | Not reported | Not reported | Not reported |
| Reeder et al., 2020 | 18 | 24 | Mean: 19.77 | 24 | 131 | Not-reported | Not-reported |
| Ruprecht et al., 2020 | 21 | 70 | Not-reported | 64 | 107 | 64 | 107 |
| Sharareh et al., 2020 | Not-reported | Not-reported | Not-reported | Not-reported | Not-reported | Not-reported | Not-reported |
| Sharkey and Schoenberg, 2002 | 60 | 103 | Mean: 79 | 335 | 729 | 335 | 729 |
| Sharkey and Schoenberg, 2005 | 61 | 98 | Median: 79 | 125 | 268 | 125 | 268 |
| Siddiqi et al., 2021 | Not-reported | Not-reported | Mean: 62 | 537 | 598 | 537 | 598 |
| Stallings et al., 2016 | Not reported | Not reported | Not reported | 148 | 149 | 148 | 149 |
| Stewart et al., 2011 | 18 | 76 | Mean: 42.5 | 364 | 476 | 364 | 476 |
| Stockman et al., 2020 | 18 | Not-reported | Median: 33 | 60 | 473 | 60 | 473 |
| Stuff et al., 2004 | Not reported | Not reported | Not reported | 807 | 1662 | 807 | 1662 |
| Tackett et al., 2018 | Not reported | Not reported | Not reported | 40 | 183 | 40 | 183 |
| Tamargo et al., 2021 | Not-reported | Not-reported | Mean: 53.9 | 283 | 394 | 283 | 394 |
| Tan et al., 2020 | Not-reported | Not-reported | Median: 49.6 | 914 | 1324 | 914 | 1324 |
| Tong et al., 2019 | Not reported | Not reported | Median: 58 | 279 | 350 | Not applicable | Not applicable |
| Trego et al., 2019 | 20 | Not reported | Not reported | 2616 | 11220 | Not reported | Not reported |
| Tucker-Seeley et al., 2016 | Not reported | Not reported | Mean: 66.6 | 415 | 3074 | 415 | 3074 |
| Vedovato et al., 2016 | Not reported | Not reported | Not reported | 298 | 298 | 298 | 298 |
| Walker et al., 2020 | 18 | 65 | Not-reported | 36099 | 287836 | 36099 | 287836 |
| Wang et al., 2015 | Not reported | Not reported | Not reported | 4336 | 6709 | 4336 | 6709 |
| Wang et al., 2021 | Not-reported | 79 | Not-reported | 80 | 213 | 80 | 213 |
| Weaver et al., 2019 | Not reported | Not reported | Mean: 20.9 | 216 | 2055 | 216 | 2055 |
| Whitbeck et al., 2006 | 16 | 19 | Mean: 17.4 | 94 | 428 | Not applicable | Not applicable |
| Wilson et al., 2006 | Not reported | Not reported | Mean: 59.1 | 55 | 98 | 55 | 98 |
| Wolfe et al., 1996 | 60 | 89 | Not reported | 16 | 41 | 16 | 35 |
| Wood and Harris, 2018 | 18 | Not reported | Not reported | 718 | 6103 | 718 | 6103 |
| Yu et al., 2010 | 18 | 79 | Mean: 35.2 | 710 | 3799 | 710 | 3799 |
