# Supplementary material for: Food insecurity among African Americans in the United States: A scoping review
Source: PLoS One. 2022 Sep 12;17(9):e0274434. doi: 10.1371/journal.pone.0274434 (PMC9467341; doi:10.1371/journal.pone.0274434)
Supplement: S3 Appendix — (DOCX) [file pone.0274434.s003.docx]

**Appendix C: Screening Forms**

**Title and abstract screening:** Title/Abstract screening was performed using the following questions, with response options “yes”, “no”, or “unclear”:

1. Is the title/abstract available in English?
   1. Yes, include and proceed to Q2 (If there is no abstract but the title is in English, answer “Yes”)
   2. No, exclude; (specify language) _______________
2. Is the primary research study describing food (in)security metrics among African American adults in the United States?
   1. Yes, include and proceed to full-text screening.
   2. No, exclude.
   3. Unclear, proceed to full-text screening.
3. Comments ___________________

**Full-text screening:** Full-text screening was performed using the following questions:

1. Is the full text available in English?

a. Yes, include and proceed to next question.

b. No, exclude; (specify language) ___________

1. Does the full-text article describe a primary research study?
   1. Yes, include and proceed to next question.
   2. No, exclude.
2. Does the full-text article include the population of interest (African American adults in the United States)?
   1. Yes, include/proceed to next question.
   2. No, exclude.
3. Does the full text evaluate food (in)security or potential metrics of food insecurity such as availability (NOT just availability only in the home but outside the home), supply, intake, deprivation, utilization, or use of Food Assistance Programs?
   1. Yes, include and proceed to next question.
   2. No, exclude.
4. Does the study design have a comparison group?
   1. Yes, include and proceed to next question.
   2. No, exclude.
5. Does the study assess individual- or group-level factors associated with food insecurity?
   1. Yes, proceed to data extraction.
   2. No, exclude.
6. Comments ________________

**Data Extraction Strategy**

**Study Characteristics**

1. Reference ID #
2. What is the Study ID (If the article describes only 1 study, this is the same as the Reference ID#)?
3. Indicate the state in which the population resides
4. Indicate the region(s) (urban or rural) in which the population resides
5. Enter the spread of the age of the participants
   - Range
   - Standard Deviation
   - Standard Error
   - 95% Confidence Interval
   - Standard Deviation/Standard Error not specified
   - Not Reported
   - IQR
6. Enter the central tendency of the age of the participants
   - Mean
   - Median
   - Not Reported
7. What is the number of participants of the population of interest in the study? (Give the number of African American participants)?
8. What is the total number of participants in the study (including the ones who are not African American)?
9. What is the total number of African American households sampled in the study?
10. What is the total number of households in the study (including the ones that are not African American)?
11. What was the study design?
12. Additional Comments

**Food Security Outcomes**

1. Reference ID #
2. What is the Study ID (If the article describes only 1 study, this is the same as the Reference ID#)?
3. What is the metric of food insecurity reported?
4. What was the authors’ exact definition of the food insecurity metrics?
5. What exposures/risk factors were examined?
6. Which dimensions of food security (accessibility, availability, and utilization) are represented by each risk factor identified?
7. Additional Comments
