# Supplementary material for: Food insecurity among African Americans in the United States: A scoping review
Source: PLoS One. 2022 Sep 12;17(9):e0274434. doi: 10.1371/journal.pone.0274434 (PMC9467341; doi:10.1371/journal.pone.0274434)
Supplement: S1 File — (DOCX) [file pone.0274434.s005.docx]

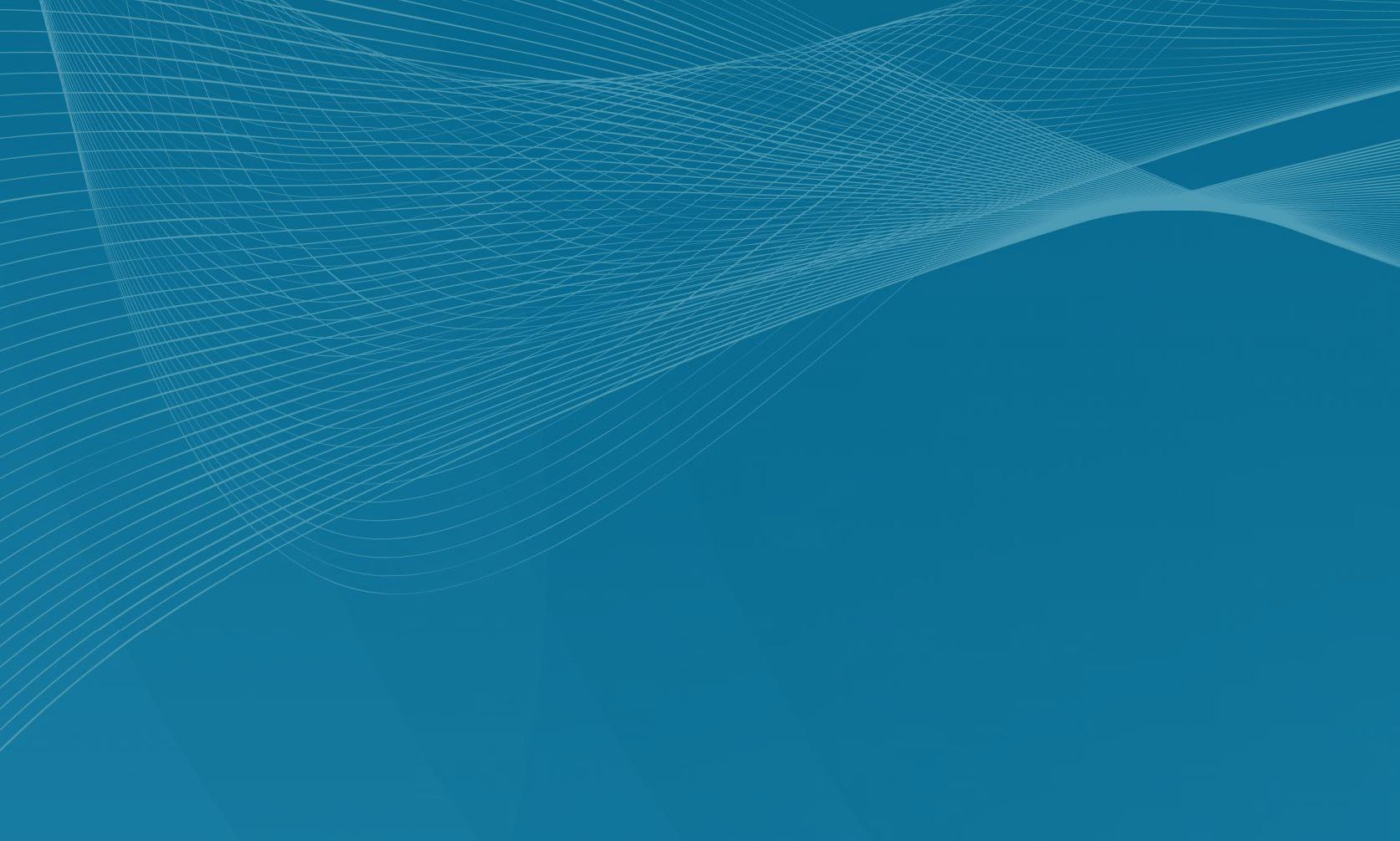


| Campbell Policies and Guidelines Series No. 4 |
| --- |
| October 2019 |
|  |
|  |
|  |
|  |
|  |
|  |
|  |
|  |
|  |
| **Methodological expectations of Campbell Collaboration intervention reviews: Reporting standards** |


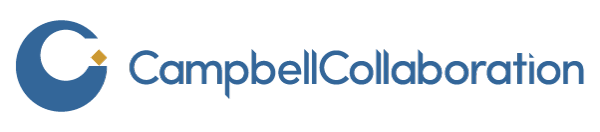


Colophon

| **Title** | |  | | Methodological expectations of Campbell Collaboration intervention reviews: Reporting standards |
| --- | --- | --- | --- | --- |
| **Authors** | |  | | The Methods Coordinating Group of the Campbell Collaboration |
| **DOI** | |  | | 10.4073/cpg.2016.4 |
| **No. of pages** | |  | | 23 |
| **Last updated** | |  | | 29 October, 2019 |
| **Citation** | |  | | The Methods Group of the Campbell Collaboration. Methodological expectations of Campbell Collaboration intervention reviews: Reporting standards  Campbell Policies and Guidelines Series No. 4  DOI: 10.4073/cpg.2016.4 |
| **ISSN** | |  | | 2535-2458 |
| **Copyright** | |  | | © The Campbell Collaboration  This is an open-access article distributed under the terms of the Creative Commons Attribution License, which permits unrestricted use, distribution,  and reproduction in any medium, provided the original author and source are credited. |
| **Acknowledgement** | |  | | Adaptations on MECIR Version 2.2 Reporting Standards (Chandler, Churchill, Higgins, Lasserson, & Tovey, 2012). Updated with new Cochrane Handbook reference in Oct 29, 2019 |
| **Editor in Chief** |  | | Vivian Welch, University of Ottawa, Canada | |
| **Chief Executive Officer** |  | | Howard White, The Campbell Collaboration | |
| **Managing Editor** |  | | Chui Hsia Yong, The Campbell Collaboration | |
|  |  | | The Campbell Collaboration was founded on the principle that systematic reviews on the effects of interventions will inform and help improve policy and services. Campbell offers editorial and methodological support to review authors throughout the process of producing a systematic review. A number of Campbell's editors, librarians, methodologists and external peer-reviewers contribute. | |
|  |  | | The Campbell Collaboration  P.O. Box 7004  St. Olavs plass  0130 Oslo, Norway  [www.campbellcollaboration.org](http://www.campbellcollaboration.org) | |
|  | |  | |  |

Note to authors:

This document provides detailed methodological expectations for the reporting of Campbell Collaboration systematic reviews of ***intervention effects***. It is important to note that some Campbell reviews may not focus on intervention effects, but may synthesize observational research that is policy relevant. For instance, such reviews may examine correlational or descriptive research, diagnostic or test accuracy, or other topics that do not necessarily focus on intervention effects. Although most of the methodological expectations listed below will be appropriate for all review topics (intervention focused or not), some (particularly those related to study design) may not be entirely applicable to non-intervention reviews, and have been noted as such under the ‘rationale and elaboration’ column. Authors of non-intervention reviews should seek guidance from their Campbell Coordinating Group editorial team and/or the Methods Coordinating Group in those situations.

Status: Mandatory means that a new review will not be published if this standard is not met. Highly desirable means that this should generally be done but that there are justifiable exceptions. There may be legitimate variation between or within Campbell Coordinating Groups in the relative emphasis placed on compliance with highly desirable standards. The emphasis placed on compliance with highly desirable standards will remain at the discretion of each Campbell Coordinating Group. Optional means this is done at the authors’ discretion.

Authors may refer to the Campbell Policies and Guidance and the Cochrane Handbook for additional details about items (references below).

| **Item No.** | **Status** | **Item Name** | **Standard** | **Rationale and elaboration** | **Authors note: pages where item is addressed** |
| --- | --- | --- | --- | --- | --- |
| Title and authors | | | | |  |
| R1 | Highly desirable | Format of title | Follow the standard template for a Campbell review title. |  | Pg. 1 |
| R2 | Mandatory | Authors | List names and affiliations of all authors |  | Pg. 1 |
| Abstract | | | | |  |
| R3 | Mandatory | Writing the abstract | Prepare a structured abstract to provide a succinct summary of the review. In the interests of brevity it is highly desirable for authors to provide an abstract of less than 700 words, and it should be no more than 1000 words in length. | Abstracts are a prominent, publicly accessible summary of the review. They should convey key information about the review question and its findings, and be informative to readers. [PRISMA item 2] | Pg. 2 |
| R4 | Mandatory | Abstract, Background | Summarize the rationale and context of the review. |  | Pg. 2 |
| R5 | Mandatory | Abstract, Objectives | State the main objective(s), preferably in a single concise sentence. | The objective(s) should be expressed in terms that relate to the population(s), intervention comparison(s) and, where appropriate, outcomes of interest. | Pg. 2 |
| R6 | Mandatory | Abstract, Search methods | Provide the date of the last search from which records were evaluated and any studies identified were incorporated into the review, and an indication of the databases and other sources searched. | Abstracts should aim to give readers brief but key information about the comprehensiveness of the search and the currency of the information summarized by the review.  The abstract must include the month and year of the set of searches up to which the conclusions of the review are valid. This date should reflect the date of the most recent set of searches from which all records have been screened for relevance and any studies meeting the eligibility criteria have been fully incorporated into the review (studies may be awaiting classification if, for example, the review authors are awaiting translation or clarification from authors or sponsors).  The amount of information regarding the search should be indicative of the process rather than provide specific details. In the interests of brevity certain details regarding the overall process may need to be moved to the full text of the review.  Example: “MEDLINE, PsycINFO, five other databases and three trials registers were searched on [date] together with reference checking, citation searching and contact with study authors to identify additional studies.” | Pg. 2 |
| R7 | Mandatory | Abstract, Selection criteria | Summarize eligibility criteria of the review, including information on study design, population, and comparison. | Any extensions to eligibility criteria to address adverse effects, economic issues, or qualitative research should be mentioned. | Pg. 2 |
| R8 | Mandatory | Abstract, Data collection and analysis | Summarize any noteworthy methods for selecting studies, collecting data, evaluating risk of bias/study quality, and synthesizing findings. For many reviews, it may be sufficient to state “We used standard methodological procedures expected by The Campbell Collaboration.” | This section of the abstract should indicate the rigor of the methods that underpin the results reported subsequently in the abstract. It does not need to replicate detailed description of the methods in the main text of the review.  Details of how many people were involved in the screening process and collection of information about any included studies are not necessary in the abstract. Key statistical methods may be given if not clear from the results that follow.  The abstract should prioritize the disclosure of non-standard approaches. For example, rather than disclosing all domains applied in the assessment of risk of bias/study quality, notable variations on the standard approach should be given, such as non-standard tools that were used. | Pg. 2 |
| R9 | Mandatory | Abstract, Main results: number of studies and participants | Report the number of included studies and participants. | The total number of included studies should be stated. It might be appropriate to provide numbers of studies and participants for specific comparisons and main outcomes if the amount of evidence differs substantially from the total. Numbers of participants *analyzed* should generally be presented in preference to numbers *recruited* (e.g., randomized); more important is to be clear which numbers are being reported. For some types of data there may be preferable alternatives to the number of participants (e.g., person-years of follow-up). | Pg. 2 |
| R10 | Highly desirable | Abstract, Main results: study characteristics | Provide a brief description of key characteristics that will determine the applicability of the body of evidence (e.g., age, severity of condition, setting, study duration). | Summarizing the study characteristics will provide readers of the abstract with important information about the applicability of the included studies. This is particularly important if the included studies reflect a subgroup of those eligible for inclusion in the review, for example, if the review intended to address the effects of interventions across all age groups, but included studies that only recruited adolescents. | Pg. 2 |
| R11 | Mandatory | Abstract, Main results: bias/quality assessment | Provide a comment on the findings of the risk of bias/quality assessments. | The risk of bias/study quality assessments are a key finding and form a fundamental part of the strength of the conclusions drawn in the review. If risks of bias/study quality differ substantially for different comparisons and outcomes, this may need to be mentioned. | Not required for Scoping Review |
| R12 | Mandatory | Abstract, Main results: findings | Report findings for all primary outcomes, irrespective of the strength and direction of the result, and of the availability of data. | Findings should typically include concise information about the quality of the body of evidence for the outcome (such as study limitations, consistency of effect, imprecision, indirectness and publication bias).  Outcomes should not be selected solely on the basis of the findings. If no studies measured the primary outcomes, then a comment should be made to that effect. | Pg. 2 |
| R13 | Highly desirable | Abstract, Main results: adverse effects | Ensure that any findings related to adverse effects are reported. If adverse effects data were sought, but availability of data was limited, this should be reported. | The abstract of the review should aim to reflect a balanced summary of the benefits and harms of the intervention. | Not required for Scoping Review |
| R14 | Mandatory | Abstract, Main results: format of numerical results | Present summaries of statistical analyses in the same way as they are reported in the review and in a standard way, ensuring that readers will understand the direction of benefit and the measurement scale used, and that confidence intervals are included where appropriate. | The standard format for reporting the results of statistical analysis includes an indication of the summary measure, point estimate and confidence interval (e.g., odds ratio 0.75, 95% CI [0.62 to 0.89]). | Not required for Scoping Review |
| R15 | Highly desirable | Abstract, Main results: interpretability of findings | Ensure that key findings are interpretable, or are re-expressed in an interpretable way. For instance, they might be re-expressed in absolute terms (e.g., assumed and corresponding risks, NNTs, group means), and outcomes combined with a standardized scale (e.g., SMD) might be re-expressed in units that are more naturally understood. | Absolute effects provide a useful illustration of the likely impact of intervention, and are usually easier to understand than relative effects. Units expressed on a standardized scale reflect the effect estimate as the number of standard deviations. This is not intuitive to many readers who may be more familiar with specific scales. Any re-expressed findings must have been presented in the same way in the main text of the review (see previous standard). | Not required for Scoping Review |
| R16 | Mandatory | Abstract, Implications for policy, practice, and research | State key conclusions drawn. | Authors’ conclusions may include both implications for practice and implications for research. Care must be taken to avoid interpreting lack of evidence of effect as evidence of lack of effect. *Recommendations* for practice should be avoided | Pg. 2 |
| R17 | Mandatory | Completeness of main review text | Ensure that all findings reported in the abstract, including re-expressions of meta-analysis results, also appear in the main text of the review. |  | Not required for Scoping Review |
| R18 | Mandatory | Consistency of summary versions of the review | Ensure that reporting of objectives, important outcomes, results, caveats and conclusions is consistent across the text, the abstract and the ‘Summary of findings’ table (if included). | Summary versions of the review should be written on the assumption that they are likely to be read in isolation from the rest of the review. | Pg. 2 |
| Background | | | | |  |
| R19 | Mandatory | Background | Provide a concise description of the condition or problem addressed by the review question, definition of the intervention and how it might work, and why it is important to do the review. | Systematic reviews should have a clearly defined and well-reasoned rationale that has been developed in the context of existing knowledge. Outlining the context of the review question is useful to readers and helps to establish key uncertainties that the review intends to address.  [PRISMA item 3] | Pg. 3 |
| R20 | Highly desirable | Background headings | Include the four standard headings when writing the Background. | Four standard headings are recommended (‘Description of the problem or condition’, ‘Description of the intervention’, ‘How the intervention might work’, and ‘Why it is important to do this review’). Different headings should only be used when these standard headings are not appropriate for the content matter of the review. | Pg. 3  Our review does not include an intervention of interest. We followed a P (population); O (outcome) framework. |
| R21 | Mandatory | Background references | Support all key supporting statements with references. | Claims or statements regarding aspects such as prevalence and mechanisms of action should be substantiated and, where available, supported by external evidence. | Pg. 3 |
| R22 | Mandatory | Background text | Do not use plagiarized text. | Unacknowledged copying from the work of other people is not acceptable. There may however be situations in which the same text appears in different reviews, for example when the reviews are prepared by the same team.  Content that is identical to, drawn or copied from standard texts may be acceptable but must be referenced. Ensure any verbatim quotations of more than a few words are shown in quotation marks and clearly acknowledge (i.e., cite) all sources. | Pg. 3 |
| R23 | Mandatory | Main objective | State the main objective, where appropriate in a single concise sentence. | The primary objective of a Campbell review should be to assess the effects of one or more interventions on stakeholder-important outcomes, both intended and unintended. The objective should be expressed in terms that relate to the population(s), intervention, comparison(s) and, where appropriate to specify explicitly, the outcomes of interest. Stakeholders may be professionals, service providers, policy makers, practitioners, or others.  *MECCIR C2* (Define in advance the objectives of the review, including participants, interventions, comparators, and outcomes.)  Where possible, the format should be of the form “To assess the effects of *[intervention or comparison]* for *[topical issue]* for/in *[types of people, issue or problem and setting if specified]*”.  [PRISMA item 4] | Pg. 4 |
| R24 | Mandatory, if applicable | Secondary objectives | *If the review includes secondary objectives*, state explicitly (as secondary objectives) any specific questions being addressed by the review, such as those relating to particular participant groups, intervention comparisons, or outcomes. | The objectives should be expressed in terms that relate to the population(s), intervention comparison(s) and, where appropriate, outcomes of interest.  *MECCIR C4* (Consider in advance whether issues of equity and relevance of evidence to specific populations are important to the review, and plan for appropriate methods to address them if they are. Attention should be paid to the relevance of the review question to populations such as low socioeconomic groups, low or middle-income regions, women, people with disabilities, children, and older people.) | Pg. 4 |
| R25 | Mandatory | Economic evidence | *If economic evidence is being reviewed*, state this explicitly in the Objectives (as secondary objectives). | The primary aim of a Campbell review should be to assess the effects of one or more interventions on stakeholder-important outcomes, both intended and unintended. These outcomes may include economic outcomes, such as the impact of interventions on use of resources and/or costs. If economic evidence is being reviewed as an integrated economics component, this should be stated as a secondary objective. | Not required for Scoping Review |
| R26 | Mandatory, if applicable | Qualitative research evidence | *If qualitative research evidence is being reviewed*, state this explicitly in the Objectives (as secondary objectives). | The primary aim of a Campbell review should be to assess the effects of one or more interventions on stakeholder-important outcomes, both intended and unintended. If qualitative research evidence is being included to ‘complement the review, this should be stated as a secondary objective. | Not required for Scoping Review |
| Methods | | | | |  |
| R27 | Mandatory | Reference protocol | Cite the protocol for the review. | The reader should be made aware that the review is based on a published protocol. This is particularly important if the review has been split into multiple reviews since the protocol was published. The protocol should be cited using the last publication citation for the protocol. Archived versions of protocols can be accessed via the current version of the review in the Campbell Systematic Reviews.  [PRISMA item 5] | Pg. 5 |
| Criteria for inclusion and exclusion of studies in the review | | | | |  |
| R28 | Mandatory | Eligibility criteria for types of study: study designs | State eligible study designs, and provide a justification for the choice. | It is not necessary to explain why randomized trials are eligible (if that is the case), although it may be important to explain the eligibility or non-eligibility of other types of study designs.  *MECCIR C9* (Define in advance the eligibility criteria for study designs in a clear and unambiguous way, with a focus on features of a study's design rather than design labels.)  *MECCIR C11* (Justify the choice of eligible study designs.)  [PRISMA item 6] | Pg. 5 |
| R29 | Mandatory | Eligibility criteria for types of study: study reports | *If studies are excluded on the basis of language of publication*, explain and justify this. | Studies should be included irrespective of their publication status, and electronic availability. If studies are excluded based on their language of publication, explicit justification for this exclusion should be provided.  *MECCIR C12* (Include studies irrespective of their publication status, unless explicitly justified.)  [PRISMA item 6] | Pg. 5 |
| R30 | Mandatory | Eligibility criteria for types of participants | State eligibility criteria for participants, including any criteria around location, setting, status, or definition of condition and demographic factors, and how studies including subsets of relevant participants are handled. | Any notable restrictions on the eligibility criteria of the review should be given and explained (e.g., exclusion of people under or over a certain age, specific settings of intervention).  *MECCIR C5* (Define in advance the eligibility criteria for participants in the studies.)  *MECCIR C6* (Define in advance how studies that include only a subset of relevant participants will be handled.)  [PRISMA item 6] | Pg. 6 |
| R31 | Mandatory | Eligibility criteria for types of interventions | State eligibility criteria for interventions and comparators, including any criteria around delivery, dose, duration, intensity, co-interventions, and characteristics of complex interventions. | *MECCIR C7* (Define in advance the eligible interventions and the interventions against which these can be compared in the included studies.)  [PRISMA item 6] | Our review does not include an intervention of interest. |
| R32 | Mandatory | Role of outcomes | *If measurement of particular outcomes is used as an eligibility criterion*, state and justify this. | Studies should never be excluded from a review solely because no outcomes of interest are *reported*. However, on occasion it will be appropriate to include only studies that *measured* particular outcomes. For example, a review of a multi-component school improvement intervention promoting varied increases in student outcomes might legitimately exclude studies that do not measure student achievement.  *MECCIR C8* (Clarify in advance whether outcomes listed under ’Criteria for inclusion and exclusion of studies in the review' are used as criteria for including studies (rather than as a list of the outcomes of interest within whichever studies are included).)  [PRISMA item 6] | Pg. 7 |
| R33 | Mandatory | Outcomes of interest | State primary and secondary outcomes of interest to the review, and define acceptable ways of measuring them. | Explain how multiple variants of outcome measures (e.g., definitions, assessors, scales, time points) are addressed. The *primary outcomes* should be as few as possible (ideally no more than three).  *MECCIR C14* (Define in advance which outcomes are primary outcomes and which are secondary outcomes.)  Also, *MECCIR C15 – C18*. | Pg. 7 |
| Search methods for identification of studies | | | | |  |
| R34 | Mandatory | Search sources | List all sources searched, including: databases, trials registers, web sites, and grey literature. Database names should include platform/provider name and dates of coverage; web sites should include full name and URL. State whether reference lists were searched and whether individuals or organizations were contacted. | *MECCIR C36* (Document the search process in enough detail to ensure that it can be reported correctly in the review.)  *Also, MECCIR C24 – C31.*  [PRISMA item 7] | Pg. 7 |
| R35 | Mandatory | Latest searches | Provide the date of the last search and the issue/version number (where relevant) for each database whose results were evaluated and incorporated into the review. If a search was re-run prior to publication, the results of which were not incorporated, explain how the results were dealt with and provide the date. | The review should provide the search date from which studies have been retrieved and assessed for inclusion. This is the date up to which the conclusions of the review are valid. It should reflect the date of the most recent set of searches from which all records have been screened for relevance and any studies meeting the eligibility criteria have been fully incorporated into the review (studies may be awaiting classification if, for example, the review authors are awaiting translation or clarification from authors or sponsors).  Since the review is likely to have drawn on searches conducted across multiple databases, it is possible that searches were performed on more than one date. The earliest date of the most recent set of searches should be provided in the review text and as the hard-coded date of the last search. The remaining dates for other databases should be reported in an appendix.  If a ‘catch-up’ search was run subsequent to the review being written up, any relevant studies not yet assessed for inclusion should be listed in the section ‘Studies awaiting assessment’.  *MECCIR C37* (Rerun or update searches for all relevant databases within 12 months before publication of the review or review update, and screen the results for potentially eligible studies.)  *MECCIR C38* (Incorporate fully any studies identified in the rerun or update of the search within 12 months before publication of the review or review update.)  [PRISMA item 7] | Pg. 7 |
| R36 | Mandatory | Search restrictions | Specify and justify any restrictions placed on the time period covered by the search. | *MECCIR C35* (Justify the use of any restrictions in the search strategy on publication date, publication format or language.) | Pg. 7 |
| R37 | Mandatory | Searches for different types of evidence | *If the review has specific eligibility criteria to include additional studies such as studies of adverse effects, economics evidence or qualitative research evidence*, describe search methods for identifying such studies. | Some reviews extend beyond a focus on the effects of interventions and address specific additional types of evidence.  *MECCIR C26* (*If the review has specific eligibility criteria around study design to address adverse effects, economic issues or qualitative research questions*, undertake searches to address them.) | Pg. 7 |
| R38 | Mandatory | Search strategies for bibliographic databases | Present the exact search strategy (or strategies) used for each database in an Appendix, including any limits and filters used, so that it could be replicated. | Search strategies that are available elsewhere (e.g., standard methodological filters, or strategies used to populate a specialized register) may be referenced rather than reproduced. Including numbers of hits for each line in the strategy is optional.  *MECCIR C36* (Document the search process in enough detail to ensure that it can be reported correctly in the review.)  Also, *MECCIR C32 – C35*.  [PRISMA item 8] | Pg. 7-8 |
| R39 | Highly desirable | Search strategies for other sources | Report the search terms used to search any sources other than bibliographic databases (e.g., trials registers, the web, direct contact with primary study authors), and the dates of the searches. | Some of this information might be best placed in an Appendix.  *MECCIR C36* (Document the search process in enough detail to ensure that it can be reported correctly in the review.) | Not required for Scoping Review |
| Data collection and analysis | | | | |  |
| R40 | Mandatory | Inclusion decisions | State how inclusion decisions were made (i.e. from search results to included studies), clarifying how many people were involved and whether they worked independently. | *MECCIR C39* (Highly desirable to use (at least) two people working independently to determine whether each study meets the eligibility criteria, and define in advance the process for resolving disagreements.)  [PRISMA item 9] | Pg. 8 |
| R41 | Mandatory | Data collection process | State how data were extracted from reports of included studies, clarifying how many people were involved (and whether independently), and how disagreements were handled. Describe data collection process for any reports requiring translation. | *MECCIR C43* (Use a data collection form, which has been piloted.)  *MECCIR C45* (Highly desirable to use (at least) two people working independently to extract study characteristics from reports of each study, and define in advance the process for resolving disagreements.)  [PRISMA item 10] | Pg. 8 |
| R42 | Highly desirable | Requests for data | Describe attempts to obtain or clarify data from individuals or organizations. | *MECCIR C48* (Seek key unpublished information that is missing from reports of included studies.)  [PRISMA item 10] | Not required for Scoping Review |
| R43 | Mandatory | Data items | List the types of information that were sought from reports of included studies. | *MECCIR C44* (Collect characteristics of the included studies in sufficient detail to populate final tables.)  [PRISMA item 11] | Pg. 8-9 |
| R44 | Mandatory | Transformations of data | Explain any transformations of reported data prior to presentation in the review, along with any assumptions made. Explain any procedures for extracting numeric data from graphs. | *MECCIR C46* (Collect and utilize the most detailed numerical data that might facilitate similar analyses of included studies. Where 2×2 tables or means and standard deviations are not available, this might include effect estimates (e.g., odds ratios, regression coefficients), confidence intervals, test statistics (e.g., t, F, Z, chi-squared), or p-values, or even data for individual participants.) | Not required for Scoping Review |
| R45 | Highly desirable | Missing outcome data | Explain how missing outcome data were handled. | Describe how assumptions are applied for missing data, e.g., last observation carried forward, or assumptions of particular values such as worst-case or best-case scenarios. | Not required for Scoping Review |
| R46 | Mandatory | Tools to assess risk of bias/study quality in individual studies | State the tool(s) or coding strategies used to assess the primary study quality/risk of bias for included studies, how the tool(s) or coding strategies were implemented, and the criteria used to assign studies, for example, to judgments of low risk, high risk, and unclear risk of bias; low quality or high quality. | *MECCIR C51* (Assess the study quality or risk of bias for each included study. For randomized trials, the Cochrane 'Risk of bias' tool might be used, involving judgments and supports for those judgments across a series of domains of bias  *MECCIR C52 – C60.*  [PRISMA item12] | Pg. 9 |
| R47 Mandatory | | Using effect measures | Explain what effect measures were used in the review | State the effect measures (and their corresponding variances) used by the review authors to describe effect sizes (e.g., risk ratio, mean difference) in any included studies and/or meta-analyses. | Not required for Scoping Review |
| R48 | Mandatory | Quantitative synthesis | Describe any methods for combining results across studies (e.g., meta-analysis, subgroup analysis, meta-regression, sensitivity analysis), including methods for assessing heterogeneity (e.g., I2, tau-squared, statistical test). Reference the software and command/macro/program used for analyses. | *MECCIR C62* (Undertake (or display) a meta-analysis only if participants, interventions, comparisons and outcomes are judged to be sufficiently similar to ensure an answer that is meaningful.)  *MECCIR C63* (Assess the presence and extent of between-study variation when undertaking a meta-analysis.)  [PRISMA items 12, 13, 14 and 16] | Not required for Scoping Review |
| R49 | Mandatory | Addressing risk of bias/study quality | Describe how studies with low quality or high/variable risks of bias are addressed in the synthesis. | *MECCIR C*59 (Address risk of bias/study quality in the synthesis (whether qualitative or quantitative). For example, present analyses stratified according to key risk of bias/quality items, or restricted to studies at low risk of bias/with high quality). | Pg. 9 |
| R50 | Mandatory | Non-standard designs | *If designs other than individually randomized, parallel-group randomized trials are included*, describe any methods used to address clustering, matching or other design features of the included studies. | *MECCIR C70* (Consider the impact on the analysis of clustering, matching or other non-standard design features of the included studies.) | Not required for Scoping Review |
| R51 | Mandatory | Studies with more than two groups | *If multi-arm studies are included*, explain how they are addressed and incorporated into syntheses. | *MECCIR C66* (*If multi-arm studies are included*, analyses multiple intervention groups in an appropriate way that avoids arbitrary omission of relevant groups and double-counting of participants.) | Not required for Scoping Review |
| R52 | Highly desirable | Risk of reporting bias across studies | Describe any methods used for assessing the risk of reporting biases such as publication bias. | [PRISMA item 15] | Not required for Scoping Review |
| R53 | Mandatory | Moderator analyses | *If moderator analysis (subgroup or meta-regression analyses) was performed*, state the potential effect modifiers with rationale for each, stating whether each was defined *a priori* or *post hoc*. | *MECCIR C22* (Pre-define potential effect modifiers (e.g., used in subgroup or meta-regression analyses) at the protocol stage; restrict these in number; and provide rationale for each.)  [PRISMA item 16] | Not required for Scoping Review |
| R54 | Highly desirable | Summary of findings | State any methods for summarizing the findings of the review, including the assessment of the quality of the body of evidence for each outcome. | *MECCIR C75* (If applicable, include a ‘Summary of Findings’ table according to recommendations described in the Cochrane Handbook (version 5 or later). Specifically:  •include results for one population group (with few exceptions);  •indicate the intervention and the comparison intervention;  •include seven or fewer participant-important outcomes;  •describe the outcomes (e.g., scale, scores, follow-up);  •indicate the number of participants and studies for each outcome;  •present at least one baseline risk for each dichotomous outcome (e.g., study population or median/medium risk) and baseline scores for continuous outcomes (if appropriate);  •summarize the intervention effect (if appropriate); and  •include a measure of the quality of the body of evidence)  *MECCIR C76* (Use the five GRADE considerations (study limitations, consistency of effect, imprecision, indirectness and publication bias) to assess the quality of the body of evidence for each outcome, and to draw conclusions about the quality of evidence within the text of the review.)  [PRISMA item 12] | *Not required for Scoping Review* |
| Results | | | | |  |
| Description of studies | | | | |  |
| R55 | Mandatory | Flow of studies | Provide information on the flow of studies from the number(s) of references identified in the search to the number of studies included in the review, ideally using a flow chart. Clarify how multiple references for the same study relate to the individual studies. | *MECCIR C41* (Document the selection process in sufficient detail to complete a PRISMA flow chart and a table of ‘Characteristics of excluded studies’.  *(MECCIR C42* (Collate multiple reports of the same study, so that each study rather than each report is the unit of interest in the review.)  [PRISMA item 17] | Figure 1 |
| R56 | Highly desirable | Lack of included studies | *If a review identifies no eligible studies*, restrict the Results section to a description of the flow of studies and any brief comments about reasons for exclusion of studies. | Under ‘Risk of bias/quality in included studies’ and ‘Effects of interventions’, state “No study met the eligibility criteria’. Any discussion of evidence not meeting the eligibility criteria of the review should be in the Discussion section. | Not required for Scoping Review |
| R57 | Mandatory | Excluded studies | List key excluded studies (i.e., those a reader might reasonably have expected to find) and provide justification for each exclusion. | The table of ‘Characteristics of excluded studies’ is intended as an aid to users rather than a comprehensive list of studies that were identified but not included. List here any studies that a user might reasonably expect to find in the review to explain why it is excluded. | Pg. 9 |
|  | | | | |  |
| R58 | Highly desirable | Studies awaiting classification | List the characteristics of any studies that have been identified as potentially eligible but have not been incorporated into the review. | Users of the review will be interested to learn of any potentially relevant studies that have been conducted which are known to the review team but have not yet been incorporated in to the review. This will help them to assess the stability of the review findings. These should be listed in the table of ‘Characteristics of studies awaiting classification’, along with any details that are known. | Not required for Scoping Review |
| R59 | Highly desirable | Ongoing studies | Provide details of any identified studies that have not been completed. | Users of the review will be interested to learn of any potentially relevant studies that have not been completed. This will help them to assess the stability of the review findings. These should be listed in the table of ‘Characteristics of ongoing studies’, along with any details that are known. | Not required for Scoping Review |
| R60 | Highly desirable | Table of ‘Characteristics of included studies’ | It is highly desirable to present a table of ‘Characteristics of included studies’ using a uniform format across all studies. For large reviews including hundreds of studies, such a table may not be appropriate, in which case the review should include detailed descriptive statistics for the included studies. | MECCIR C44 (Collect characteristics of the included studies in sufficient detail to populate final tables)  [PRISMA item 18] | Appendix B:  Figure S4 – S6 |
| R61 | Mandatory | Included studies | Provide a brief narrative summary of any included studies. This should include the number of participants and a summary of the characteristics of the study populations and settings, interventions, comparators and funding sources. |  | Pg. 10 - 11 |
| R62 | Highly desirable | Table of ‘Characteristics of included studies’: sample sizes | Include the sample size (and effective sample size for cluster assigned studies) for each included study in the table of ‘Characteristics of included studies’. | If sample sizes are available for each intervention group, these should be included. A convenient place is often within the box for Interventions (e.g., inserting “(n=50))” after each listed intervention group. | Not required for Scoping Review |
| R63 | Highly desirable | Table of ‘Characteristics of included studies’: methods | Provide the basic study design or design features (e.g., parallel group randomized trial, cluster-randomized trial, controlled before and after study). | Even if the review is restricted to one study design, these tables should provide a comprehensive summary of each study.  It is important that labels used to describe study designs are clearly defined in the review.  [PRISMA item 18] | Appendix B:  Figure S4 – S6 |
| R64 | Highly desirable | Table of ‘Characteristics of included studies’: participants | Provide sufficient information about the study populations to enable a user of the review to assess the applicability of the review’s findings to their own setting. | Information presented in this table should reflect the baseline demographics of the study sample. [PRISMA item 18] | Appendix B:  Figure S4 – S6 |
| R65 | Highly desirable | Table of ‘Characteristics of included studies’: interventions | Provide sufficient information to enable users of the review to assess the applicability of the intervention to their own setting, and if possible in a way that allows the intervention to be replicated. | For example, for education interventions consider intervention, educational context, frequency and duration of doses; or for complex interventions, specify the core components of the intervention. Lengthy explanations of interventions should be avoided. Citations to sources of detailed descriptions can be included.  [PRISMA item 18] | Not required for Scoping Review |
| R66 | Highly desirable | Table of ‘Characteristics of included studies’: outcomes | Provide clear and consistent information about outcomes measured (or reported), how they were measured and the times at which they were measured. | It should be clear whether main outcomes of interest in the review were measured in the study. | Appendix B:  Figure S4 – S6 |
| R67 | Highly desirable | Table of ‘Characteristics of included studies’: dates | Include the dates when the study was conducted in the table of ‘Characteristics of included studies’. | If dates are not available then this should be stated (e.g., “Study dates not reported”).  [PRISMA item 18] | Appendix B:  Figure S4 – S6 |
| R68 | Highly desirable | Table of ‘Characteristics of included studies’: funding source | Include details of funding sources for the study, where available. | Details of funding sources should be placed in this table. Including an extra row in the table of ‘Characteristics of included studies’ is encouraged. | Not required for Scoping Review |
| R69 | Highly desirable | Table of ‘Characteristics of included studies’: declarations of interest | Include details of any declarations of interest among the primary researchers. | Declarations of interest should be placed in this table. Including an extra row in the table of ‘Characteristics of included studies’ is encouraged. | Not required for Scoping Review |
| R70 | Highly desirable | Choice of intervention groups in multi-arm studies | *If a study is included with more than two intervention arms*, restrict comments on any irrelevant arms to a brief comment in the table of ‘Characteristics of included studies’. | Intervention arms that are not relevant to the review question should not be discussed in detail, although it is useful to clarify (in this table) that such arms were present.  *MECCIR C49* (*If a study is included with more than two intervention arms*, include in the review only intervention and control groups that meet the eligibility criteria.) | Not required for Scoping Review |
| R71 | Mandatory | References to included studies | List all reports of each included study under the relevant Study ID. | [PRISMA item 18] | Appendix B:  Figure S4 – S6 |
| Study quality/risk of bias in included studies | | | | |  |
| R72 | Mandatory | ‘Risk of bias’ and/or study quality table | Present a ‘Risk of bias’ and/or “Study Quality” table for each included study, with judgments about risks of bias, and explicit supports for these judgments. | *MECCIR C51:* Assess the risk of bias/study quality for each included study. If the review is co-registered or is reviewing randomized trials, the Cochrane 'Risk of bias' tool should be used, involving judgments and supports for those judgments across a series of domains of bias, as described the Cochrane Handbook (version 5 or later).If not, then one of the many other risk of bias and/or study quality tools should be utilized and detailed within the protocol prior to implementation.  Also *MECCIR C53 – C60*  [PRISMA item 19] | *Not required for Scoping Review* |
| R73 | Highly desirable | Summary assessments of risk of bias/study quality | Summarize the study quality/risk of bias across domains for each key outcome for each included study, and ensure that these are supported by the information presented in the ‘Risk of bias’ and/or “Study Quality” tables. | *MECCIR C58* (Summarize the risk of bias/study quality for each key outcome for each study.)  [PRISMA item 22] | *Not required for Scoping Review* |
| R74 | Mandatory | Study quality/risk of bias in included studies | Provide a brief narrative summary of the quality/risks of bias among the included studies [PRISMA items 22 and 25] | It may be helpful to identify any studies considered to be at low risk of bias for particular key outcomes or with high quality for specific methodological characteristics. | Not required for Scoping Review |
| Effects of interventions | | | | |  |
| R75 | Mandatory | Use of ‘Data and analysis’ headings | Ensure appropriate use of the hierarchy of Comparisons / Outcomes / Subgroups / Study data in the ‘Data and analysis’ section. | Appropriate use of the hierarchy ensures consistency of structure across reviews. It is confusing for the user if outcomes are listed against the heading ‘Comparison’ and interventions listed against the heading ‘Outcome or subgroup’. | Not required for Scoping Review |
| R76 | Highly desirable | Presenting data | Ensure that simple summary data for each intervention group, as well as estimates of effect size (comparing the intervention groups), are available for each study for each outcome of interest to the review. | Simple summaries such as numbers of events, means and standard deviations should be presented for each treatment group when available. This is achieved primarily by using the ‘Data and analyses’ section of the review, for dichotomous and continuous outcomes. For other outcomes, these should typically be presented in tables of ‘Other data’.  [PRISMA item 20] | Not required for Scoping Review |
| R77 | Mandatory | Number of studies and participants | State how many studies and how many participants contributed data to results for each outcome, along with the proportion of the included studies and recruited participants potentially available for the relevant comparison. | It is unlikely that the same number of studies will contribute data to every outcome of interest. Specific studies may contribute different numbers of participants for different outcomes. Therefore, for each comparison, it is helpful to indicate to readers what proportion of the relevant included studies and recruited participants contribute data to each outcome. Failing to disclose this may be misleading.  [PRISMA item 9] | Not required for Scoping Review |
| R78 | Highly desirable | Source of data | State the source of all data presented in the review, in particular, whether it was obtained from published literature, by correspondence, from a trials register, from a web-based data repository, etc. | Transparency of data source enables validation or verification of data by others including editors or readers of the review. | Not required for Scoping Review |
| R79 | Mandatory | Multiple outcome data | Describe any *post hoc* decisions that might give rise to accusations of selective outcome reporting, for example when there are multiple outcome measures (e.g., different scales), multiple time points or multiple ways of presenting results. | Transparent disclosure of post-hoc decisions will enable readers of the review to assess the credibility of the results of the review for themselves.  *MECCIR C16* (Define in advance details of what are acceptable outcome measures (e.g., diagnostic criteria, scales, composite outcomes).)  *MECCIR C17* (Define in advance how outcome measures will be selected when there are several possible measures (e.g., multiple definitions, assessors, or scales)).  *MECCIR C18* (Define in advance the timing of outcome measurement.) | Not required for Scoping Review |
| R80 | Highly desirable | Ordering of results and ‘Data and analysis’ section | Organize results to follow the order of comparisons and outcomes specified in the protocol, following in particular the distinction between primary and secondary outcomes. | Review authors must avoid selectively reporting analysis results in a way that depends on the findings. The best way to achieve this is to follow a well-structured protocol and present results as outlined in that protocol. However, sometimes a pragmatic decision needs to be made that an alternative arrangement is preferable, particularly with regard to comparisons. This choice should be explicitly justified. | Not required for Scoping Review |
| R81 | Mandatory | Pre-specified outcomes | Report synthesis results for all pre-specified outcomes, irrespective of the strength or direction of the result. Indicate whether data were not available for outcomes of interest, including whether harms were identified. | To avoid selective outcome reporting (in truth or in perception), the review should address all outcomes specified in the protocol.  [PRISMA item 20] | Not required for Scoping Review |
| R82 | Mandatory | Statistical uncertainty | Accompany all effect size estimates with a measure of statistical uncertainty (e.g., a confidence interval with a specified level of confidence such as 90%, 95% or 99%). | Confidence intervals are the preferred method for expressing statistical uncertainty.  [PRISMA item 20] | Not required for Scoping Review |
| R83 | Highly desirable | P values | *If reporting p-values*, provide exact p-values (e.g., p = 0.08 rather than p > 0.05). | Effect estimates with confidence intervals are the preferred method of presenting numeric results. P-values should not be used as an alternative to confidence intervals and should not be used to divide results into ‘significant’ or ‘non-significant’. | Not required for Scoping Review |
| R84 | Mandatory | Tables and Figures | Link to each Table and Figure. |  | 11 Tables; 2 Figures |
| R85 | Highly desirable | Number of Tables and Figures | Restrict the number of Tables and Figures to a small number (six or less) to convey key findings without affecting the readability of the review text. | Tables and Figures may be added to reviews and included in the body of the text. Reviews should try to avoid including a large number of Tables and Figures, unless they are necessary and appropriate to display review findings. Additional or supplementary Tables and Figures can be included as appendices, as appropriate. | 11 tables; 2 figures; |
| R86 | Mandatory | Consistency of results | Ensure that all statistical results presented in the main review text are consistent between the text and the ‘Data and analysis’ tables. | MECCIR C47, C50 | Pg. 10-21 |
| R87 | Mandatory | Different scales | Explain how studies measuring an outcome of interest using different scales (such as alternative rating scales that measure symptoms or behavior) were combined, stating whether positive or negative values reflect benefit or harm. | If data from different scales are combined and presented on a standardized scale (such as a standardized mean difference), it is important to clarify that a positive effect size has the same meaning for every study. The direction of benefit or harm must be stated.  *MECCIR C61* (*If studies are combined with different scales*, ensure that higher scores for continuous outcomes all have the same meaning for any particular outcome; explain the direction of interpretation; and report when directions were reversed. ) | Not required for Scoping Review |
| R88 | Mandatory | Interpretability of results | Ensure that key findings are interpretable, or are re-expressed in an interpretable way. For instance, they might be re-expressed in absolute terms (e.g., assumed and corresponding risks, NNTs, group means), and outcomes combined with a standardized scale (e.g., SMD) might be re-expressed in units that are more naturally understood. If substantively or clinically important effect sizes are well understood, these should be provided to aid interpretation. Relying on Cohen’s (1988) guidelines is not recommended given that effect sizes are generally context specific. | Absolute effects provide a useful illustration of the likely impact of intervention, and are usually easier to understand than relative effects. They may need to be accompanied, however, with information about assumed baseline risks. Confidence intervals should be presented for NNTs and similar summary measures. Re-expressing relative effects as absolute effects often requires the specification of assumed (e.g., untreated) risks, and the source of these should be provided. Results expressed as standardized mean differences reflect the number of standard deviations’ difference between mean responses. This is not intuitive to many readers who may be more familiar with specific scales. Substantively or clinically important effect sizes should ideally be specified in the protocol.  MECCIR C69 (consider statistical heterogeneity in interpretation)  MECCIR C72 (interpret p-values correctly) | Pg. 10-21 |
| R89 | Mandatory | Studies without usable data | Comment on the potential impact of studies that apparently measured outcomes but did not contribute data that allowed the study to be included in syntheses. | There is good evidence of selective outcome reporting among clinical trials. Outcomes that are believed to have been measured but are not reported in a usable format may therefore be systematically different from those that are usable, introducing bias. ‘Usable’ in this sense refers both to incorporation in a meta-analysis and to consideration in non-statistical syntheses of findings. Authors might consider using a table to indicate which studies contribute data to the outcomes of interest in the review.  *MECCIR C40* (Include studies in the review irrespective of whether measured outcome data are reported in a ‘usable’ way. | Not required for Scoping Review |
| R90 | Highly desirable | Missing outcome data | Discuss the implications of missing outcome data from individual participants (due to losses to follow up or exclusions from analysis). | *MECCIR C64* (Consider the implications of missing outcome data from individual participants (due to losses to follow up or exclusions from analysis).) | *Not required for Scoping Review* |
| R91 | Highly desirable | Skewed data | Discuss the possibility and implications of skewed data when analyzing continuous outcomes. | *MECCIR C65* (Consider the possibility and implications of skewed data when analyzing continuous outcomes) | *Not required for Scoping Review* |
| R92 | Highly desirable | Forest plots | Present data from multiple studies in forest plots wherever possible, providing it is reasonable to do so. | Presenting data in forest plots can be useful even if the studies are not combined in a meta-analysis.  [PRISMA item 20] | Not required for Scoping Review |
| R93 | Highly desirable | Multiple subgroup analyses and sensitivity analyses | *If presenting multiple sensitivity analyses or different ways of subgrouping the same studies*, present these in summary form (e.g., a single Table or Figure) and not in multiple forest plots. | [PRISMA item 23]  MECCIR C67, C68, C71 | Not required for Scoping Review |
| R94 | Mandatory | Labels on plots | Label the directions of effect and the intervention groups in forest plots with the interventions being compared. | Directions of effect should be used as consistently as possible within a review. | Not required for Scoping Review |
| R95 | Highly desirable | Study quality/risk of bias across studies | Present results of the assessment of study quality/risk of bias across studies (and across domains) for each key outcome and state whether this leads to concerns about the validity of the review’s findings. | Considerations of study quality/risk of bias across studies are required for assessments of the quality of the body of evidence (e.g., using GRADE).  [PRISMA item 22] | Not required for Scoping Review |
| R96 | Highly desirable | Reporting biases | Present results of any assessment of the potential impact of reporting biases on the review’s findings. | *MECCIR C73* (Consider the potential impact of reporting biases on the results of the review or the meta-analyses it contains.)  [PRISMA item 22] | *Not required for Scoping Review* |
| R97 | Optional | ‘Summary of findings’ table | If the Cochrane risk of bias tool is used, present a ‘Summary of Findings’ table according to recommendations described in the Cochrane Handbook (version 5 or later). Specifically:  include results for one clearly defined population group (with few exceptions); indicate the intervention and the comparison intervention;  include seven or fewer participant-important outcomes;  describe the outcomes (e.g., scale, scores, follow-up);  indicate the number of participants and studies for each outcome;  present at least one baseline risk for each dichotomous outcome (e.g., study population or median/medium risk) and baseline scores for continuous outcomes (if appropriate);  summarize the intervention effect (if appropriate); and include a measure of the quality of the body of evidence for each outcome. | *MECCIR C74* (Include a ‘Summary of Findings’ table according to recommendations described in the Cochrane Handbook (version 5 or later). Specifically:  •include results for one population group (with few exceptions);  •indicate the intervention and the comparison intervention;  •include seven or fewer participant-important outcomes;  •describe the outcomes (e.g., scale, scores, follow-up);  •indicate the number of participants and studies for each outcome;  •present at least one baseline risk for each dichotomous outcome (e.g., study population or median/medium risk) and baseline scores for continuous outcomes (if appropriate);  •summarize the intervention effect (if appropriate); and  •include a measure of the quality of the body of evidence.)  [PRISMA item 24] | *Not required for Scoping Review* |
| R98 | Optional | Assessments of the quality of the body of evidence | Provide justification or rationale for any measures of the quality of the body of evidence for each key outcome. If a ‘Summary of findings’ table is used, use footnotes to explain any downgrading or upgrading. | *MECCIR C75* (Use the five GRADE considerations (study limitations, consistency of effect, imprecision, indirectness and publication bias) to assess the quality of the body of evidence for each outcome, and to draw conclusions about the quality of evidence within the text of the review.)  *MECCIR C76* (Justify and document all assessments of the quality of the body of evidence (for example downgrading or upgrading if using the GRADE tool).) | *Not required for Scoping Review* |
| Discussion | | | | |  |
| R99 | Highly desirable | Discussion headings | Include the standard headings when writing the Discussion. | Six standard headings are recommended (‘Summary of main results’, ‘Overall completeness and applicability of evidence’, ‘Quality of the evidence’, ‘Potential biases in the review process, ‘Agreements and disagreements with other studies or reviews’). | Pg. 21 |
| R100 | Mandatory | Limitations | Discuss limitations of the review (e.g., incomplete identification of studies, reporting bias), and the implications of any study-level or outcome-level risk of bias/quality assessments on the review findings. | Review authors must explicitly state the limitations of their review. These limitations should be addressed in the discussion headings of ‘Quality of the evidence’ and ‘Potential biases in the review process.’ If those two headings are not used, then at minimum, the review should include a ‘Limitations’ heading that addresses all potential review limitations.  *MECCIR C73* (Consider the potential impact of reporting biases on the results of the review or the meta-analyses it contains.)  [PRISMA item 25] | Pg. 23 |
| Authors’ conclusions | | | | |  |
| R101 | Mandatory | Conclusions: implications for practice | Provide a general interpretation of the evidence so that it can inform practice or policy decisions. Avoid making recommendations for practice. | *MECCIR C77-78* (Avoid providing recommendations for practice.) | Pg. 24 |
| R102 | Mandatory | Conclusions: implications for research | *If recommending further research*, structure the implications for research to address the nature of evidence required, including population, intervention comparison, outcome, and type of study. | Researchers and research funders are an important user group of Campbell reviews. Recommendations for future research should offer constructive guidance on addressing the remaining uncertainties identified by the review. This is particularly important for reviews that identify few or no studies.  *MECCIR C79* (Structure the implications for research to address the nature of evidence required, including population intervention comparison, outcome, and type of study). | Pg. 24-25 |
| Acknowledgements | | | | |  |
| R103 | Mandatory | Acknowledgements | Acknowledge the contribution of people not listed as authors of the review, including any assistance from Campbell Coordinating Groups, non-author contributions to searching, data collection, study appraisal or statistical analysis, and the role of any funders. | [PRISMA item 27] | Not applicable |
| Contributions of authors | | | | |  |
| R104 | Mandatory | Contributions of authors | Describe the contributions of each author. |  | PLOSE ONE submission details |
| Declarations of interest | | | | |  |
| R105 | Mandatory | Declarations of interests | Report any present or past affiliations or other involvement in any organization or entity with an interest in the review’s findings that might lead to a real or perceived conflict of interest. | The nature and extent of the affiliation or involvement (whether financial or non-financial) should be described. An additional consideration for authors of systematic reviews is the declaration of involvement in studies that were included in the review. It is important to note that authors who were involved in primary studies must not be involved in the data extraction/coding/critical appraisal of those studies as part of the systematic review process. | PLOSE ONE submission details |
| Differences between protocol and review | | | | |  |
| R106 | Mandatory | Changes from the protocol | Explain and justify any changes from the protocol (including any *post hoc* decisions about eligibility criteria or the addition of subgroup or moderator analyses). | *MECCIR C13* (Justify any changes to eligibility criteria or outcomes studied. In particular, post hoc decisions about inclusion or exclusion of studies should keep faith with the objectives of the review rather than with arbitrary rules.) | PLOS ONE submission details |
| R107 | Highly desirable | Methods not implemented | Document aspects of the protocol that were not implemented (e.g., because no studies, or few studies, were found) in the section ‘Differences between protocol and review’, rather than in the Methods Section. |  | Not applicable |
| Sources of support | | | | |  |
| R108 | Mandatory | Sources of support | List sources of financial and non-financial support for the review and the role of the funder, if any. | [PRISMA item 28] | PLOSE ONE submission details |

References:

Chandler J, Churchill R, Higgins J, Lasserson T, Tovey D. Methodological standards for the conduct of new Cochrane Intervention Reviews. Version 2.2. Cochrane: London, 2012.

Higgins JPT, Thomas J, Chandler J, Cumpston M, Li T, Page MJ, Welch VA (editors). Cochrane Handbook for Systematic Reviews of Interventions version 6.0 (updated July 2019). Cochrane, 2019. Available from [www.training.cochrane.org/handbook](http://www.training.cochrane.org/handbook).

The Campbell Collaboration. Campbell systematic reviews: policies and guidelines. Campbell Policies and Guidelines Series No. 1. DOI: 10.4073/cpg.2016.1. Available at: <https://onlinelibrary.wiley.com/page/journal/18911803/homepage/author-guidelines>
