# Supplementary material for: Food insecurity among African Americans in the United States: A scoping review
Source: PLoS One. 2022 Sep 12;17(9):e0274434. doi: 10.1371/journal.pone.0274434 (PMC9467341; doi:10.1371/journal.pone.0274434)
Supplement: Attachment — Submitted filename: PONE-D-21-20858_reviewer_resp.pdf [file pone.0274434.s006.pdf]

PLOS ONE

# Food Insecurity among African Americans in the United States: An evidence and gap map

--Manuscript Draft--

| | |
|---|---|
| **Manuscript Number:** | PONE-D-21-20858 |
| **Article Type:** | Research Article |
| **Full Title:** | Food Insecurity among African Americans in the United States: An evidence and gap map |
| **Short Title:** | Food Insecurity among African Americans in the United States |
| **Corresponding Author:** | Elizabeth Dennard<br>FDA: US Food and Drug Administration<br>Laurel, MD UNITED STATES |
| **Keywords:** | African Americans; Racial/Ethnic disparities; Food Security; systematic evidence synthesis; Covid-19 |
| **Abstract:** | In 2019, the estimated prevalence of food insecurity for Black non-Hispanic households was higher than the national average due to health disparities exacerbated by forms of racial discrimination. During the COVID-19 pandemic, Black households have experienced higher rates of food insecurity when compared to other populations in the United States. The primary objectives of this review were to identify which risk factors have been investigated for an association with food insecurity, describe how food insecurity is measured across studies that have evaluated this outcome among African Americans, and determine which dimensions of food security (food accessibility, availability, and utilization) are captured by risk factors studied by authors. Food insecurity related studies were identified through a search of PubMed, CINAHL Plus, MEDLINE®, PsycINFO, Health Source: Nursing/Academic Edition, and Web of Science TM (Clarivate), on May 20, 2021. Eligible studies were primary research studies, with a concurrent comparison group, published in English between 1995 and 2021. Ninety-eight relevant studies were included for data charting with 37 unique measurement tools, 115 risk factors, and 93 possible consequences of food insecurity identified. Few studies examined factors linked to racial discrimination, behaviour, or risk factors that mapped to the food availability dimension of food security. Infrequently studied factors, such as lifetime racial discrimination, socioeconomic status (SES), and income insecurity need further investigation while frequently studied factors such as age, education, race/ethnicity, and gender need to be summarized using a systematic review approach so that risk factor impact can be better assessed. Risk factors linked to racial discrimination and food insecurity need to be better understood in order to minimize health disparities among African Americans during the COVID-19 pandemic and beyond. |
| **Order of Authors:** | Elizabeth Dennard |
| | Elizabeth Kristjansson |
| | Nedelina Tchangalova |
| | Sarah Totton |
| | Donna Winham |
| | Annette O'Connor |
| **Additional Information:** | |
| **Question** | **Response** |
| **Financial Disclosure**<br><br>Enter a financial disclosure statement that describes the sources of funding for the work included in this submission. Review | The authors received no specific funding for this work. |

| | |
|---|---|
| the submission guidelines for detailed requirements. View published research articles from *PLOS ONE* for specific examples.<br><br>This statement is required for submission and **will appear in the published article** if the submission is accepted. Please make sure it is accurate.<br><br>**Unfunded studies**<br>Enter: *The author(s) received no specific funding for this work.*<br><br>**Funded studies**<br>Enter a statement with the following details:<br>• Initials of the authors who received each award<br>• Grant numbers awarded to each author<br>• The full name of each funder<br>• URL of each funder website<br>• Did the sponsors or funders play any role in the study design, data collection and analysis, decision to publish, or preparation of the manuscript?<br>• **NO** - Include this sentence at the end of your statement: *The funders had no role in study design, data collection and analysis, decision to publish, or preparation of the manuscript.*<br>• **YES** - Specify the role(s) played.<br><br>* typeset | |
| **Competing Interests**<br><br>Use the instructions below to enter a competing interest statement for this submission. On behalf of all authors, disclose any competing interests that could be perceived to bias this work—acknowledging all financial support and any other relevant financial or non-financial competing interests.<br><br>This statement is required for submission and **will appear in the published article** if the submission is accepted. Please make sure it is accurate and that any funding sources listed in your Funding Information later in the submission form are also | The authors have declared that no competing interests exist. |

| | |
|---|---|
| declared in your Financial Disclosure statement.<br><br>View published research articles from *PLOS ONE* for specific examples.<br><br>**NO authors have competing interests**<br><br>Enter: *The authors have declared that no competing interests exist.*<br><br>**Authors with competing interests**<br><br>Enter competing interest details beginning with this statement:<br><br>*I have read the journal's policy and the authors of this manuscript have the following competing interests: [insert competing interests here]*<br><br>* typeset | |
| **Ethics Statement**<br><br>Enter an ethics statement for this submission. This statement is required if the study involved:<br><br>• Human participants<br>• Human specimens or tissue<br>• Vertebrate animals or cephalopods<br>• Vertebrate embryos or tissues<br>• Field research<br><br>Write "N/A" if the submission does not require an ethics statement.<br><br>General guidance is provided below. Consult the submission guidelines for detailed instructions. **Make sure that all information entered here is included in the Methods section of the manuscript.** | N/A |

| | |
|---|---|
| **Format for specific study types**<br><br>**Human Subject Research (involving human participants and/or tissue)**<br>• Give the name of the institutional review board or ethics committee that approved the study<br>• Include the approval number and/or a statement indicating approval of this research<br>• Indicate the form of consent obtained (written/oral) or the reason that consent was not obtained (e.g. the data were analyzed anonymously)<br><br>**Animal Research (involving vertebrate animals, embryos or tissues)**<br>• Provide the name of the Institutional Animal Care and Use Committee (IACUC) or other relevant ethics board that reviewed the study protocol, and indicate whether they approved this research or granted a formal waiver of ethical approval<br>• Include an approval number if one was obtained<br>• If the study involved *non-human primates*, add *additional details* about animal welfare and steps taken to ameliorate suffering<br>• If anesthesia, euthanasia, or any kind of animal sacrifice is part of the study, include briefly which substances and/or methods were applied<br><br>**Field Research**<br><br>Include the following details if this study involves the collection of plant, animal, or other materials from a natural setting:<br>• Field permit number<br>• Name of the institution or relevant body that granted permission | |
| **Data Availability**<br><br>Authors are required to make all data underlying the findings described fully available, without restriction, and from the time of publication. PLOS allows rare exceptions to address legal and ethical concerns. See the PLOS Data Policy and FAQ for detailed information. | Yes - all data are fully available without restriction |

Powered by Editorial Manager® and ProduXion Manager® from Aries Systems Corporation

| | |
|---|---|
| A Data Availability Statement describing where the data can be found is required at submission. Your answers to this question constitute the Data Availability Statement and **will be published in the article**, if accepted.<br><br>**Important:** Stating 'data available on request from the author' is not sufficient. If your data are only available upon request, select 'No' for the first question and explain your exceptional situation in the text box.<br><br>Do the authors confirm that all data underlying the findings described in their manuscript are fully available without restriction? | |
| **Describe where the data may be found in full sentences. If you are copying our sample text, replace any instances of XXX with the appropriate details.**<br><br>• If the data are **held or will be held in a public repository**, include URLs, accession numbers or DOIs. If this information will only be available after acceptance, indicate this by ticking the box below. For example: *All XXX files are available from the XXX database (accession number(s) XXX, XXX.).*<br>• If the data are all contained **within the manuscript and/or Supporting Information files**, enter the following: *All relevant data are within the manuscript and its Supporting Information files.*<br>• If neither of these applies but you are able to provide **details of access elsewhere**, with or without limitations, please do so. For example:<br><br>*Data cannot be shared publicly because of [XXX]. Data are available from the XXX Institutional Data Access / Ethics Committee (contact via XXX) for researchers who meet the criteria for access to confidential data.*<br><br>*The data underlying the results presented in the study are available from (include the name of the third party* | All relevant data are within the manuscript and its supporting information. |

| | |
|---|---|
| *and contact information or URL).*<br>• This text is appropriate if the data are owned by a third party and authors do not have permission to share the data.<br><br>* typeset | |
| Additional data availability information: | |

Manuscript
Click here to access/download;Manuscript;2021_07_23_Manuscript.docx

# Food insecurity among African Americans in the United States: An evidence and gap map

Elizabeth Dennard[1], Elizabeth Kristjansson[2], Nedelina Tchangalova[3], Sarah Totton[4], Donna Winham[5], and Annette O'Connor[6]

[1]Office of Applied Research and Safety Assessment, Food and Drug Administration, Laurel, Maryland, United States of America

[2]School of Psychology, University of Ottawa, Ottawa, Ontario, Canada

[3]STEM Library, University of Maryland, College Park, Maryland, United States of America

[4]Department of Population Medicine, University of Guelph, Guelph, Ontario, Canada

[5]Food Science and Human Nutrition, Iowa State University, Ames, Iowa, United States of America

[6]College of Veterinary Medicine, Michigan State University, East Lansing, Michigan, United States of America

* Corresponding author

Email: Elizabeth.dennard@fda.hhs.gov (ED)

[¶]These authors contributed equally to this work.

# Abstract

In 2019, the estimated prevalence of food insecurity for Black non-Hispanic households was higher than the national average due to health disparities exacerbated by forms of racial discrimination. During the COVID-19 pandemic, Black households have experienced higher rates of food insecurity when compared to other populations in the United States. The primary objectives of this review were to identify which risk factors have been investigated for an association with food insecurity, describe how food insecurity is measured across studies that have evaluated this outcome among African Americans, and determine which dimensions of food security (food accessibility, availability, and utilization) are captured by risk factors studied by authors. Food insecurity related studies were identified through a search of PubMed, CINAHL Plus, MEDLINE®, PsycINFO, Health Source: Nursing/Academic Edition, and Web of Science™ (Clarivate), on May 20, 2021. Eligible studies were primary research studies, with a concurrent comparison group, published in English between 1995 and 2021. Ninety-eight relevant studies were included for data charting with 37 unique measurement tools, 115 risk factors, and 93 possible consequences of food insecurity identified. Few studies examined factors linked to racial discrimination, behaviour, or risk factors that mapped to the food availability dimension of food security. Infrequently studied factors, such as lifetime racial discrimination, socioeconomic status (SES), and income insecurity need further investigation while frequently studied factors such as age, education, race/ethnicity, and gender need to be summarized using a systematic review approach so that risk factor impact can be better assessed. Risk factors linked to racial discrimination and food insecurity need to be better understood in order to minimize health disparities among African Americans during the COVID-19 pandemic and beyond.

what is the relevance of COVID?

**Keywords:** African Americans; racial/ethnic disparities; food security; systematic evidence synthesis; COVID-19

# Introduction

## Description of the problem

As of 2019, 10.5% of United States (US) households (13.7 million households) experienced food insecurity and 4.1% of these households (5.3 million households) experienced very low food security at some time during the year [1]. In 2019, rates of food insecurity were significantly higher than the national average for households with Black, non-Hispanic, household reference persons (19.1 percent) [1]. The COVID-19 pandemic has caused a public health and economic crisis with repercussions that have led to an increase in the number of people experiencing food insecurity. In 2020, African Americans experienced more negative health outcomes linked to COVID-19, the disease caused by SARS-CoV-2, than other populations due to a combination of factors including racial discrimination, disparities linked to income and health, and inconsistent access to food [2]. Further, in the United States, individual studies have reported that African American households are two to three times as likely to experience consistent food insecurity when compared to the general population [3-5] These prior findings indicate that race is associated with food insecurity. However, many individual- and group-level factors other than race have been investigated for an association with food insecurity. A comprehensive list of studied risk factors and their relationship to food insecurity among African American households is not available. A comprehensive list is needed to understand which relationships exist and which intervention opportunities need to be investigated. According to Ashby and colleagues [6], “accurate measurement of food insecurity is imperative to understand the magnitude of the issue and to identify specific areas of need, in order to effectively tailor policies and interventions for its alleviation.” To understand the implications of current study findings, each citation and corresponding findings must be placed in the context of other studies that assess food insecurity among African American adults in the United States.

covid - racial discrimination?

## Objectives

The first objective of this review was to identify factors that have been investigated for an association with food insecurity among African American adults across the peer-reviewed literature. Knowledge of these factors will identify critical research gaps and highlight areas for future research. The second objective was to describe how food insecurity is measured in studies that have evaluated this outcome among African American populations in the United States. Knowledge of food security metrics will identify how comparable current measures and potential findings are across the literature. The final objective was to map each risk factor identified or considered by researchers to the three primary dimensions of food security (food accessibility, availability, and utilization) to identify potential gaps across each dimension.

# Materials and Methods

## Protocol and registration

The protocol was drafted using the Methodological Expectations of Campbell Collaboration Intervention Reviews: Conducts Standards and the Preferred Reporting Items for Systematic Reviews and Meta-analysis extension for Scoping Review Protocols [7]. The protocol was registered with the Systematic Reviews for Animals and Food (SYREAF) on December 30, 2019 (http://www.syreaf.org/protocol/). The methodology was informed by Munn et al. (2018)'s guidance and Arksey and O'Malley (2005)'s framework [8-9]. Protocol edits included a terminology adjustment from scoping review to evidence and gap map (EGM).

## Eligibility criteria

The eligibility criteria for study inclusion were defined based on the population (P) – adult African Americans, and the outcome (O) – food insecurity. Peer-reviewed articles published in English between 1995-2021 were eligible for inclusion in this paper.

## Eligible study designs

Eligible studies were primary research studies with a concurrent comparison group: observational studies (cross-sectional, cohort, and case control), randomized controlled trials, and primary research studies that evaluated factors between time periods (before and after). Studies that assessed interventions were also included.

## Eligible participants

Relevant participants were African American adults, 18 to 64 years of age, living in the United States. If a study contained a subset of a sample that matched the population of interest, the subset of participants was included. One possible source of ambiguity among identified citations included the definition and use of the term “African American” in the literature. The United States Census Bureau adheres to the 1997 Office of Management Budget (OMB) standards on race and ethnicity, which includes five categories: Asian, Black or African American, Native Hawaiian or Pacific Islander, American Indian or Alaska Native, and White [10]. According to Rastogi and colleagues, “The Black racial category includes people who marked the 'Black or African American' checkbox. It also includes respondents who reported entries such as African American; Sub-Saharan African entries, such as Kenyan and Nigerian; and Afro-Caribbean entries, such as Haitian and Jamaican” [11]. The category for Black and African American people serves as a broad descriptor for study participants who may not share the same ethnicity, culture, or immigration status. Rastogi and colleagues explain further that “these federal standards mandate that race and Hispanic origin (ethnicity) are separate and distinct concepts and that when collecting these data via self-identification, two different questions must be used” [11]. This distinction between race and ethnicity is relevant to this evidence and gap map because the intention was to include study participants who only identify themselves as African American. Immigration status is another key factor that may have

impacted the eligible study population of interest. For this evidence and gap map, citations were excluded if the researcher's study population of interest was comprised only of immigrants or refugees.

## Eligible outcomes

The outcome of interest was food insecurity. Some authors may have used the following terms to describe food insecurity: food availability, food accessibility, food utilization, food supply, food intake, undernourishment, food deprivation, hunger, malnutrition, and use of food assistance programs. These proxy variables of food insecurity were also eligible for inclusion in this study.

## Search sources

The search for relevant studies was conducted in six databases: PubMed (US National Library of Medicine), EBSCO databases (CINAHL Plus, MEDLINE®, PsycINFO, Health Source: Nursing/Academic Edition), and Web of Science™ (Clarivate) on May 20, 2021. Both MEDLINE (EBSCO) and legacy PubMed, the old interface, were searched due to the variations of the database syntax and features. Relevant full-text publications were obtained through available subscriptions through the University of Maryland, University of Guelph, and Iowa State University Libraries. Reference lists of the included primary research articles and retrieved systematic reviews were examined to identify any relevant publications. DistillerSR® (Evidence Partners, Ottawa, Canada) software was used for article screening and data extraction.

## Search strategy

The search strategy was designed by a public health librarian in consultation with other team members. The search strategy was checked for comprehensiveness and errors against the

*PRESS Peer Review of Electronic Search Strategies Guidelines* [12]. Search strategies for each database and corresponding results are shown in Table 1 - Table 3. Results were restricted to publication year 1995-2021, English language, and peer-reviewed publications. The US Department of Agriculture (USDA) began collecting data annually regarding food access, food spending, and sources of food assistance in the United States in 1995 [13]. Therefore, this regulatory activity represents a reasonable starting point for relevant studies to be included in this paper.

*Table 1. Search string for PubMed®, conducted on May 20, 2021. Interface: PubMed® Legacy, Database coverage dates: mid-1960s to present date of search*

| **Search** | **Query** | **Items found: 11/18/19** | **Items found: 5/20/21** |
|---|---|---|---|
| #6 | #5 Filters: Publication date from 1995/01/01 to 2019/11/18; English; Adult: 19+ years; Young Adult: 19-24 years; Adult: 19-44 years; Middle Aged + Aged: 45+ years; Middle Aged: 45-64 years<br>#5 Filters Updated search: Publication date from 2019/11/19 to 2021/05/20 English; Adult: 19+ years; Young Adult: 19-24 years; Adult: 19-44 years; Middle Aged + Aged: 45+ years; Middle Aged: 45-64 years | **738** | **71** |
| #5 | #3 NOT #4 | 1,600 | 1,868 |
| #4 | "Animals"[Mesh] NOT ("Animals"[Mesh] AND "Humans"[Mesh]) | 4,639,963 | 4,831,043 |
| #3 | #1 AND #2 | 1,829 | 2,121 |

| | | | |
|---|---|---|---|
| #2 | ("African Americans"[Title/Abstract] OR "African American"[Title/Abstract] OR Black[Title/Abstract]) OR African Americans[MeSH Terms] | 182,988 | 204,820 |
| #1 | "food supply"[MeSH Terms] OR "access to food"[Title/Abstract] OR "dietary inadequacy"[Title/Abstract] OR "food access"[Title/Abstract] OR "food accessibility"[Title/Abstract] OR "food afford*"[Title/Abstract] OR "food assistance"[Title/Abstract] OR "food availability"[Title/Abstract] OR "food choice"[Title/Abstract] OR "food consumption"[Title/Abstract] OR "food deprivation"[Title/Abstract] OR "food desert"[Title/Abstract] OR "food hardship"[Title/Abstract] OR "food insecurity"[Title/Abstract] OR "food insufficien*"[Title/Abstract] OR "food intake"[Title/Abstract] OR "food poverty"[Title/Abstract] OR "food scarcity"[Title/Abstract] OR "food security"[Title/Abstract] OR "food sufficien*"[Title/Abstract] OR "food supply"[Title/Abstract] OR "food utilization"[Title/Abstract] OR "fruit[Title/Abstract] AND vegetable intake"[Title/Abstract] OR "fruit intake"[Title/Abstract] OR "vegetable | 151,265 | 170,109 |

| | | | |
|---|---|---|---|
| | intake"[Title/Abstract] OR hunger[Title/Abstract] OR malnutrition[Title/Abstract] OR "nutrition security"[Title/Abstract] OR "nutritional status"[Title/Abstract] OR "supermarket access"[Title/Abstract] OR undernourishment[Title/Abstract] | | |

*Table 2. Search string for EBSCO Databases, conducted on May 20, 2021. Interface: EBSCO Databases, Database coverage dates: Various (see table below)*

| **Databases** | **Limiters** | **Items found: 11/18/19** | **Items found: 5/20/21** |
|---|---|---|---|
| CINAHL Plus | 1995-2019, English | 1,091 | 115 |
| MEDLINE | 1995-2019, English, Peer reviewed | 744 | 233 |
| PsycINFO | 1995-2019, English, Academic journals | 498 | 25 |
| Health Source: Nursing/Academic Edition | 1995-2019, English, Peer reviewed | 327 | 36 |
| **TOTAL** | | **2,660** | **409** |

**EBSCO Search strategy:**

( (dietary N5 inadequacy) OR (food N5 (access OR accessibility OR afford* OR assistance OR availability OR choice OR consumption OR deprivation OR desert OR hardship OR insecurity OR insufficien* OR intake OR poverty OR scarcity OR security OR sufficien* OR supply OR utilization)) OR ((fruit OR vegetable) N5 intake) OR hunger OR malnutrition OR "nutrition security" OR "nutritional status" OR (supermarket N5 access) OR undernourishment ) AND ( "African American*" OR Black* ) AND ( adults OR adult OR aged OR elderly )

*Table 3. Search string for Web of Science™ (Clarivate), conducted on May 20, 2021. Interface: Web of Science™ (Core Collection), Database coverage dates: 1900-date of search present*

| **Search** | **Query** | **Items found** |
|---|---|---|

| | | |
|---|---|---|
| #1 | ((TI=(( "access to food" OR "dietary inadequacy" OR "food access" OR "food accessibility" OR "food afford*" OR "food assistance" OR "food availability" OR "food choice" OR "food consumption" OR "food deprivation" OR "food desert" OR "food hardship" OR "food insecurity" OR "food insufficien*" OR "food intake" OR "food poverty" OR "food scarcity" OR "food security" OR "food sufficien*" OR "food supply" OR "food utilization" OR "fruit and vegetable intake" OR "fruit intake" OR "vegetable intake" OR hunger OR malnutrition OR "nutrition security" OR "nutritional status" OR "supermarket access" OR undernourishment )) AND TS=(( "African American*" OR Black* ))) NOT SU=("Veterinary Sciences" OR Agriculture OR Entomology OR Fisheries OR Forestry OR "Plant Sciences" OR Zoology)) | 470 |

**Limiters:**

LANGUAGE: (English)

DOCUMENT TYPES: (Article)

Indexes=SCI-EXPANDED, SSCI, A&HCI, CPCI-S, CPCI-SSH, BKCI-S, BKCI-SSH, ESCI, CCR-EXPANDED, IC Timespan=1995-2019

**Total results:** 398 (72 from updated search on May 20, 2021)

## Selection of sources

Search results were uploaded into EndNote X9 Desktop and duplicate records removed. Title/abstract screening, full-text screening, and data extraction were independently performed

by two authors in DistillerSR®. Both reviewers received training prior to the screening process using piloted forms and discussion until agreement about interpretation was reached. The title/abstract screening form was piloted with 100 records while the full-text screening form was piloted with five records. Conflicts were resolved through discussion until consensus was reached based on detailed justifications provided by each reviewer. The screening forms are included in Appendix A.

# Data charting and analysis

Data charting forms were developed and reviewed to determine study characteristics and data items for extraction. Two reviewers independently captured data items, discussed findings, and updated all forms as changes were made. Data extraction forms are included in Appendix A.

## Data items and extraction

Data extraction captured general study characteristics, study population characteristics (state, region, age distribution, and number of participants), study design, exposures investigated, and relevant measures. Multiple measures of food insecurity were expected. These food insecurity metrics might be used at the individual level to represent the experiences, behaviours, or conditions of an individual or a single household [1]. Alternatively, these metrics might be aggregated to represent a group at the ecological or group level. For example, a study might report the proportion of households in a region that skip meals more than twice in one week or the proportion of households in a neighbourhood with a cut-off listed in the USDA (2018)'s 18-item questionnaire. For this evidence and gap map, all measures of food security described in the literature were extracted.

## Risk of bias and study quality

According to Munn and colleagues (2018) “the purpose of evidence and gap maps (EGMs) is similar to scoping reviews which is to identify and analyse gaps within a knowledge base. Scoping reviews do not produce critically appraised or synthesized results for a given research question” [8]. Therefore, authors did not assess the methodological limitations of risk of bias of the evidence included within this evidence and gap map (EGM).

## Critical appraisal of individual sources of evidence

A critical appraisal of the included studies was not conducted, consistent with Arksey and O’Malley (2005)’s guidance [9].

## Synthesis of results

After data extraction, the factors were mapped to up to three of the four hierarchical dimensions of food security: food availability, food accessibility, and food utilization. According to Ashby and colleagues, "food availability” refers to a reliable and consistent source of enough quality food for an active and healthy lifestyle. "Food accessibility" acknowledges the resources required in order to obtain and put food on the table; this could be economic or physical. "Food utilization" refers to the intake of safe food and the human resources required to transform food into meals. "Food stability", the fourth dimension of food security, recognizes that food insecurity can be transitory, cyclical, or chronic. Ultimately, food stability can be achieved when all three domains (availability, accessibility, and utilization) become sustainable over an extended period of time [6]. For this reason, the dimension of food stability was excluded from the categorization of individual-level and group-level exposure characteristics.

The extracted risk factors were also mapped as being at the individual or group level and whether a risk factor appeared to be a “cause” or “possible consequence” of food insecurity. If

a risk factor identified in the study served as a "possible consequence" of food insecurity, this term was not categorized into the food security dimensions (food availability, accessibility, and utilization) for risk factors. For example, a study participant's mental health status or "depression score" could serve as both a "cause" of food insecurity due to lack of food accessibility or it could serve as a "consequence" of experiencing food insecurity due to lack of food utilization. If the risk factor fell into the "cause" category only, the factor was categorized based on the three food security dimensions described above. Finally, these variables were placed into ten descriptive categories: demographic (individual characteristics such as age and sex), household (marital status and single parent status), economic (household income and family poverty), behavioural (lifestyle habits, actions, and behaviours), nutritional, physical environment (physical, chemical, and biological factors external to a person), social environment (social factors external to a person), physical health (physical and genetic health factors), mental health, and COVID-19 related risk factors. This process was completed by two reviewers and then conflicts were resolved to ensure consistent classification.

# Results

## Selection of citations

The results of the search and eligibility screening process are presented in Figure 1. PRISMA 2020 Flow Diagram for New Systematic Reviews which Included Searches of Databases [14].

## Characteristics of included studies

The characteristics of included studies are described in Table 4 - Table 6. Table 4 provides an overview of food security measures and the authors' exact definition of food security. Table 5 provides an overview of study characteristics including state(s) in which the population

resides, region(s) in which the population resides, study design, and risk factors investigated. Information on study participants' age (including lower age limits, higher age limits, and the central tendency of age), the total number of African American participants and households, and the total number of study participants and households are presented in Table 6.

*Table 4. Identified metrics and definitions of food security (sorted by metrics)*

| Citation | Food Security Metric | Authors' Definition of the Food Security Metric |
|---|---|---|
| Ahluwalia et al., 2013 | WIC receipt | "We estimated reliability by kappa coefficient and validity by sensitivity and specificity using the birth certificate data as the reference for the following: prenatal participation in the Special Supplemental Nutrition Program for Women, Infants, and Children (WIC); Medicaid payment for delivery; and breastfeeding initiation" |
| Baek, 2016 | Current Population Survey Food Security Supplement (CPS-FSS) | "I use the number of vehicles operated in urbanized areas (UA) from the National Transit Database (NTD) and household food insecurity data from the Current Population Survey Food Security Supplement (CPS-FSS) from 2006 to 2009" |
| Baer et al., 2015 | U.S. Household Food Security Survey Module | "To measure food insecurity, participants were administered the appropriate validated USDA-FSS based on age and parental status. The 18-item US Household Food Security Survey Module was answered by patients aged 18-25 years who self-identified as being a parent. The 10-item Adult Food Security Survey Module was completed by patients aged 18-25 years who did not self-identify as being a parent" |
| Balistreri, 2016 | Eighteen-Item Household Food Security Scale | "The household food security scale was developed by the USDA to measure the severity of food insecurity experienced in the household in the previous 12 months. It is measured with an 18-item scale if the household contains children and ten if it does not" |
| Barnidge et al., 2017 | Eighteen-Item Household Food Security Scale | "A two-item validated screener was used to assess household food security. The first item asked, 'over the last 12 months did you worry there would not be enough food and there was no money to buy more?' "Nearly one-fifth (17.5 %) of caregivers reported this was often true while 37.3 % reported this as sometimes true. Household food insecurity was measured using the first two questions from the 18-itemU.S. Household Food Security Survey" |
| Barnidge et al., 2017 | Eighteen-Item Household Food Security Scale | "Participants completed a demographic questionnaire that included age, household income, and participation in food assistance programs. The questionnaire also included the US Department of Agriculture's 18-item Food Security Module to determine household food security status and the food security status of children in the home" |

| | | |
|---|---|---|
| Boone-Heinonen et al., 2015 | Neighborhood Supermarket Density per 10,000 people | “Neighborhood food and physical activity amenities were obtained from Dun and Bradstreet, a commercial dataset of U.S. businesses (Dun & Bradstreet). Fast-food chain restaurants, supermarkets (large grocery stores), commercial physical activity facilities, and public physical activity facilities corresponding to each CARDIA exam period were extracted and classified according to 8-digit Standard Industrial Classification codes (U.S. Department of Labor)” |
| Borders et al., 2015 | U.S. Household Food Security Survey Module | “External stressors were measured using the Home Hardships Scale, the USDA Household Food Security Scale and the Neighborhood Satisfaction Scale” |
| Brewer et al., 2010 | Six-Item Short Form of the Food Security Survey Module | “Food insecurity was assessed using the modified 6-item US Household Food Security Survey Module” |
| Burke et al., 2018 | U.S. Household Food Security Survey Module | “Food security status was our dependent variable and was measured using the US Department of Agriculture (USDA) Household Food Security Survey Module (HFSSM) (Coleman-Jensen et al.2015). The HFSSM uses a 12-month recall period and 18 Likert-type items to assess the frequency in which household members report, because of a lack of resources, disruptions in the quality, quantity and patterns of the household food supply as well as anxiety related to running out of food” |
| Caraballo et al., 2020 | 10-item questionnaire recommended by the US Department of Agriculture Economic Research Service | "Food security in the past 30 days was created based on the 10-item questionnaire as recommended by the US Department of Agriculture Economic Research Service (Table I in the Data Supplement) 13,20 and constructed following the NHIS instructions.21 Answers of ≥3 days were considered affirmative in questions about frequency of occurrence in the past 30 days. A raw score ranging from 0 to 10 was calculated, and participants were categorized as follows: 0 to 2 points: food secure; 3 to 5 points: low food security; and 6 to 10 points: very low food security. We then defined food insecurity as having either low or very low food security, in concordance with previous studies" |
| Chakrabarti et al., 2021 | Food insufficiency | "Pandemic-related food insufficiency, defined as there sometimes or often not being enough food to eat in the last 7 days or food availability becoming worse after March 13, 2020" |
| Chilton and Booth, 2007 | Six-Item Short Form of the Food Security Survey Module | “Each participant, in both the focus groups and the individual interview sessions, also answered a brief questionnaire that included demographic characteristics, food stamp participation, and the US Department of Agriculture Household Food Security Scale—Short Form. Food Insecurity was calculated according to established methods. Terminology of food insecurity categories was recently changed by the US Department of Agriculture” |
| Clay and Ross, 2020 | 2-Item screen to identify families at risk for food insecurity | "The outcome food security was assessed with a validated two-item food security screener (97 percent sensitivity, 83 percent specificity) that was designed to rapidly identify individuals at-risk for food insecurity" |

| | | |
|---|---|---|
| Conlon et al., 2015 | Six-Item Short Form of the Food Security Survey Module | “Household food security was assessed by parents’ self-response to six items from the Short Form of the Household Food Security Scale” |
| Cook et al., 2002 | U.S. Household Food Security Survey Module | “We used a somewhat more stringent method of scoring the Food Security Scale for this analysis than the standard US Department of Agriculture method, which led to conservative estimates of the effects of exposure on this outcome” |
| Cox and Wallace, 2016 | 18 food security questions in the CFSM | “There are 18 food security questions in the CFSM. The food security module is designed to allow administrators to implement two common screens (and a third less common screen) when it appears the food security questions may pose an unnecessary burden on the respondents. All three screens were used in the FFCWS survey” |
| Crabtree and Mushi-Brunt, 2013 | National Health Interview Survey on Disability (NHIS-D) with 10 questions about food access | “The NHIS-D includes questions about participation in several activities and functional limitations. We identified 10 questions about food access-related participation and functional limitations that likely affect being able to access food for nutrition” |
| Dean et al., 2011 | Radimer-Cornell hunger and food insecurity instrument | “Food insecurity was measured by the household-hunger dimension of the food-depletion item from the Radimer-Cornell hunger and food insecurity instrument” |
| Denney et al., 2020 | Eighteen-Item Household Food Security Scale | "Household food insecurity, a binary measure created from the USDA’s 18-item food insecurity scale (Bickel et al., 2000), serves as our outcome measure" |
| Duke et al., 2021 | 2-item Hunger Vital Sign Tool | "Food insecurity was assessed using the 2-item Hunger Vital Sign Tool. The baseline survey included the validated, two-item Hunger Vital Sign (HVS), a food insecurity screening tool based on the U.S. Household Food Security Survey Module" |
| Duke, 2021 | Radimer-Cornell hunger and food insecurity instrument | "Food insecurity was measured with the following question: ‘During the past 30 days, have you had to skip meals because your family did not have enough money to buy food?’ Students were able to respond ‘yes’ or ‘no’ to this question. Based on the Radimer/Cornell Hunger Scale,19 this single item focused on the experience of skipping meals represents child level or severe food insecurity, reflecting insufficiency of household food such that youth are affected by reductions in the quantity of food eaten" |
| Echeverria et al., 2004 | Access to healthy foods scale | “Scales were used to assess six neighborhood domains: aesthetic quality, walking/exercise environment, safety from crime, violence, access to healthy foods, and social cohesion” |

| | | |
|---|---|---|
| Fleming et al., 2021 | NHANES Food Security Module | "NHANES has been using the Food Security Survey Module, similar to the module included in the Current Population Survey, to assess food security since 1999. This module is included in the family questionnaire portion of the NHANES household interview. An adult family member, typically the head of household, answers the family questionnaire on behalf of the entire family and questions refer to all household members. Households with children younger than 18 years of age receive an additional 8 questions for a total of 18 items, compared to households without children" |
| Garrett-Peters and Mills-Koonce, 2013 | Food insufficiency questions (5 items) | "Mothers completed food insufficiency questions (five items) about whether they or other household members went hungry or had to skip or cut meals because they could not afford to buy food in the last 12 months. Scores could range from 0 to 5" |
| Ghosh and Parish, 2015 | Not reported | "These included a measure of difficulty paying for utilities like electricity and or telephone bills; difficulty paying the full amount for rent or mortgage, difficulty meeting essential expenses and not going to a doctor and or a dentist when needed to any time in the last 12 months, and whether they experienced food insecurity any time in the last four months" |
| Gilbert and Ashley, 2020 | 4-item 7-point Likert scale adapted from food access questionnaire from Hendrickson, Smith, and Eikenberry (2006) | "We adapted food access questionnaire items from Hendrickson, Smith, and Eikenberry (2006), who investigated access to fruits and vegetables by low-income residents in urban and rural areas of Minnesota. We created a four-item, seven-point Likert scale in which respondents indicated the extent to which they agreed or disagreed with statements such as "I have access to a variety of foods" and "the fruits and vegetables I can access are fresh." Each item is scored from 1 ("strongly disagree") to 7 ("strongly agree")" |
| Gilbert et al., 2017 | Participation in MAP + TANF + SNAP | "In addition to household income verification and documentation for WIC program eligibility, adjunctive eligibility measures, such as participation in other federal programs (i.e., medical assistance, food stamp (SNAP), and temporary cash assistance (TCA)) were gathered and electronically verified through linkage with respective agency databases prior to documentation and storage in the WOW database" |
| Hammer et al., 2021 | 3-item adaptation from the USDA Food Insecurity Scale | "Food insecurity was assessed based on responses to three food insecurity items derived from the USDA Household Food Security Survey Module.27 The items used asked how often, in the last 12 months, the respondent or people in the respondent's household (1) worried whether your food would run out before you had money to buy more; (2) the food that you bought did not last, and you didn't have enough money to get more; or (3) you couldn't afford to eat balanced meals" |

| | | |
|---|---|---|
| Harrison et al., 2005 | Six-Item Short Form of the Food Security Survey Module | “The food security measure used is an abbreviated six-item scale derived from the 18-item U.S. Household Food Security Instrument employed surveys and administered to CHIS respondents below 200% FPL” |
| Hernandez and Pressler, 2013 | Eighteen-Item Household Food Security Scale | “The measure of food insecurity is based on an 18-item scale developed by the U.S. Department of Agriculture that assesses both the quality and quantity of food over the past 12 months. The scale captures food hardship due to financial constraints such as running out of food, perceptions that food in a household is of inadequate quality or quantity, and reduced food intake by adults or children” |
| Huang et al., 2015 | Survey of Income and Program Participation. The SIPP | “The food insufficiency question in SIPP asked respondents to choose the best of the following statements describing household food experiences in each of 4 reference months:” |
| Kaiser et al., 2007 | Six-Item Short Form of the Food Security Survey Module | “Food insecurity of the women was measured by a 6-item subset of the Food Security Module” |
| Keene et al., 2015 | Survey of Income and Program Participation. The SIPP | “SIPP provides a nationally representative sample of rent-assisted households, to examine the association between housing-assistance type and reports that neighbors count on each other, watch each other’s children, help each other out, can trust each other to intervene in the face of danger or harm, and have access to help from friends to family” |
| Kharmats et al., 2014 | U.S. Adult Food Security Survey Module | “Food security over the past year was measured and scored by using the US Adult Food Security Survey Module (10 items). Participants were asked to tell the interviewer whether statements about food situations presented in the module were “often true, sometimes true, or never true for you/your household in the past 12 months” |
| Kipke et al., 2020 | Not reported | "Food security (last 12 months)" |
| Koh et al., 2020 | Six-Item Short Form of the Food Security Survey Module | "Using the Six-Item Household Food Security Survey Module designed by USDA, the FMTS elicited responses to five questions and statements about respondents’ food procurement experience in the last 12 months" |
| Laraia et al., 2006 | Eighteen-Item Household Food Security Scale | "Food security—main outcome. “The USDA food security modules comprised of 18 questions posed in increasing levels of severity by measuring the dimensions of concern about food quantity and food quality over the last 12 months” |
| Laraia et al., 2009 | Six-Item Short Form of the Food Security Survey Module | “To calculate household food insecurity status, the six-item short form of the USDA Core Food Security Module (CFSM) for families was used (29,30). Questions were asked about the household’s experience over the past 12 months” |

| | | |
|---|---|---|
| Lauren et al., 2021 | 2-Item screen to identify families at risk for food insecurity | "We assessed household risk for food insecurity using a validated two-item screen. (16) Households at risk for food insecurity were defined as those with responses of "Sometimes true" or "Often true" for either or both items. Participants reported answers to each of these questions for the periods before and after the COVID-19 outbreak" |
| Lee et al., 2011 | Six-Item Short Form of the Food Security Survey Module | "Scoring for the modified 6-item HFSSM was completed to reflect the validated survey module. For the identical questions from the original HFSSM, responses of ''often'' or ''sometimes'' on questions ''food didn't last' 'and ''couldn't afford balanced meals'' and ''yes'' on ''ate less'' and ''hungry' 'were coded as affirmative" |
| Leigh and Medal-Herrero, 2015 | WIC receipt | "Our dependent variable, was binary and equaled 1 if the subject or anyone in the household received WIC benefits in the preceding 2 years" |
| Lise et al., 2021 | Eighteen-Item Household Food Security Scale | "Household food security was assessed using the 18-item USDA US-Household Food Security Survey Module (HFSSM), which queries the past 12 months" |
| Lin et al., 2021 | Food Insecurity Experience Scale (FIES) | "We also included the Food and Agriculture Organization's Food Insecurity Experience Scale (FIES), which captures respondent's reporting of any food deprivation (e.g., constraints on one's ability to obtain adequate food) both prior to and during the pandemic" |
| Lombe et al., 2009 | Eighteen-Item Household Food Security Scale | "Household Food Security—the dependent variable—is measured using items taken from the USDA 18-item Core Food Security Module (CFSM), a self-report measure of household food security for the past 12 months" |
| Long et al., 2020 | U.S. Household Food Security Survey Module | "Household food security status was assessed in the NHIS and NHPI-NHIS via the 10-item US Adult Food Security Survey Module (Table 1).54 All respondents were asked the 10-item module, as opposed to the full battery of 18 items that are asked of households with children in the CPS" |
| Makelarski et al., 2015 | 2-item screen derived from the 18-item US Household Food Security Screen | "We measured household food insecurity in the past 12 months by using a validated 2-item screen derived from the 18-item US Household Food Security Screen (affirmative responses to either survey item indicated a positive screen for household food insecurity" |
| Martin et al., 2004 | U.S. Household Food Security Survey Module | "Household food security and hunger were measured using the US Household Food Security Module" |
| Martin et al., 2016 | Eighteen-Item Household Food Security Scale | "Food security was measured using the USDA 18-item Food Security Module" |

| | | |
|---|---|---|
| McCurdy and Metallinos-Katsaras, 2011 | 4 Items from the 18-Item Food Security Core Module (FSCM) | “Household food security status was assessed with caregiver Reponses to 4 items from the 18-item Food Security Core Module (FSCM), commonly used to measure food security status in the United States.24Caregiverswere asked about the following for the previous 12-month time period: (1) not having enough money to buy food for a balanced meal, (2) adults cutting the size of meals or skipping meals, (3) frequency of cutting or skipping meals, and (4) adults not eating for a whole day” |
| McDonough et al., 2019 | 18-item Food Security Core Module (FSCM) | "Using definitions of food security provided by the USDA, we assign each household to a particular state of food security where the various states of food security are food insecure, marginal food secure, and high food secure (i.e., K = 3). We then track how households move through the distribution of food security from one period to the next. Additionally, the food security questions surveyed come from the USDA’s Core Food Security Module" |
| Miller et al., 1996 | Nutrition Screening Initiative (NSI)& 10-item DETERMINE Checklist | “Nutritional risk was measured using the Nutrition Screening Initiative Checklist. Demographic information, economic status, self-rated health, Geriatric Depression Scale score, and body mass index were assessed using established standardized instruments” |
| Miller et al., 2015 | Number of full-service retail food outlets (RFOs) in the neighborhood | “Food stores within one’s census tract should be fairly accessible with or without automobile access or other transportation arrangements. In this article we simply measure food availability, or the presence of full service RFOs that offer a selection of fresh or frozen fruits and vegetables” |
| Mook et al., 2016 | Six-Item Short Form of the Food Security Survey Module | “Data on food security status were collected by using the US Department of Agriculture’s 6-item short-form food security scale (21). Food security status was categorized as food secure (raw score 0–1) or food insecure (raw score 2–6) and analyzed as a dichotomous variable (21)” |
| Moore et al., 2020 | Six-Item Short Form of the Food Security Survey Module | "The food security survey utilized questions from a validated survey, the US Household Food Security Survey Module: Six-Item Short Form developed by the United States Department of Agriculture (USDA).17 The sum of affirmative responses to six questions was used to calculate a raw score that determined the level of food security as food secure (score 0–1 = high security or marginal insecurity), low food security (score 2–4), or very low food security (score 5–6)" |

| | | |
|---|---|---|
| Morales et al., 2020 | Household Pulse Survey (HPS) food insecurity measure | "Instead of including any items from the HFSSM scale, the HPS collected a different measure of food insecurity by asking respondents to choose a statement that best described the food eaten situation in their households over the past 7 days [42]. A recent research report confirmed that the HPS measure is a good indicator of household food insecurity and aligns with the HFSSM measure [43]. We used responses to the HPS question to construct a variable measuring the level of household food insecurity during the COVID-19 pandemic, which ranges from 0 = food secure ("enough of the kinds of food I/ we wanted to eat") to 3 = severely food insecure ("often not enough to eat")" |
| Murimi et al., 2016 | 18 food security questions in the CFSM | "The Spanish translation of the US Household Food Security Survey Module by USDA researchers was used. Standard 12 months Core Food Security Module (CFSM) questions were used with the assumption that data collected would capture events of the past 12 months" |
| Myers and Painter, 2017 | NHANES Food Security Module | "The NHANES food security module includes questions on participants' food situations at home and food assistance benefits (i.e., those associated with SNAP) received within the previous 12 months" |
| Myers et al., 2020 | U.S. Household Food Security Survey Module | "Food security status was measured through the US Household Food Security Survey Module (developed by the US Department of Agriculture), which comprises 18 items that assess the "conditions and behaviors that characterize … difficulty meeting basic food needs" Three or more affirmative responses in this module indicate food insecurity" |
| Nagata et al., 2021 | U.S. Household Food Security Survey Module | "The questions regarding food sufficiency were consistent with the U.S. Household Food Security Survey Module. Food insufficiency generally describes whether households have enough food for their families to eat and is often the most extreme form of food insecurity. In other surveys, such as the Current Population Survey's Food Security Supplement, 86%–89% of people reporting food insufficiency were deemed food insecure" |
| Nam et al., 2014 | Food Insufficiency Indicator (from SEED OK survey) | "The dependent variable in this study is a food insufficiency indicator created with a question in SEED OK's baseline survey: "During the past 12 months, did your family (1) always have enough to eat, (2) sometimes not have enough to eat, or (3) of-ten not have enough to eat? "This item is a slightly modified version of the first screening question in the U.S. Department of Agriculture's Food Security Core-Module Questionnaire" |

| | | |
|---|---|---|
| Okafor et al., 2020 | 2-item Hunger Vital Sign Tool | "From January 2017 through February 2017, we utilized a validated two-item screening tool to assess the prevalence of households at risk for food insecurity and conducted a focus group of pediatricians. The USDA provides several tools (6-, 10-, and 18-item) to screen for food insecurity (USDA ERS: Survey Tools, 2019). In November 2015, the American Academy of Pediatrics (AAP) issued a policy statement (Promoting Food Security for All Children, 2018) that endorses the use of the two-item screening tool, now coined the Hunger Vital Sign" |
| O'Reilly et al., 2020 | 2-Item screen to identify families at risk for food insecurity | "Household food insecurity risk was measured with a 2-item screen [36]: (a) “within the past 12 months, we worried whether our food would run out before we got money to buy more” and (b) “within the past 12 months, the food we bought just didn’t last and we didn’t have money to get more.” Answering affirmatively to either or both questions indicate household food insecurity risk, coded 0 = food secure; 1 = food insecure risk" |
| Orozoco et al., 2020 | U.S. Household Food Security Survey Module | "A validated US Department of Agriculture 18-item Food Security Survey Module was used in the NHANES 2009−2014 surveys to assess household food security status over the prior 12 months. The scale consisted of statements representing coping behaviors or experiences owing to insufficient money to buy food, leading to a score of 0−18" |
| Palmer et al., 2018 | Six-Item Short Form of the Food Security Survey Module | “Food security was measured according to the six-item USDA core food security module” |
| Palmer et al., 2020 | Six-Item Short Form of the Food Security Survey Module | "The 6-item USDA Core Food Security Module (CFSM) estimated food security" |
| Paschal et al., 2020 | U.S. Adult Food Security Survey Module | "The 10-item U.S. Adult Food Security Survey Module40 was used to assess food insecurity. In using the 10-item module, direct inquiry about children’s food insecurity was avoided, which was a sensitive issue and concern for one of the partners. Survey burden was also lowered with this version compared to the 18-item module" |
| Patterson et al., 2020 | U.S. Household Food Security Survey Module | "Both NHIS and NHANES measure food security using the USDA US Food Security Survey Module (α = 0.74–0.93) (Keenan et al., 2001). The survey module asks adults to report their experiences with food security using a scale of 0–10. Levels of food security are designed as “full food security” (0 points), “marginal food security” (1–2 points), “low food security” (3–5 points), and “very low food security” (6–10 points)" |
| Payne-Sturges et al., 2018 | U.S. Household Food Security Survey Module | “Food security status was assessed via the USDA 18-item Household Food Security Survey Module (HFSSM)” |

| | | |
|---|---|---|
| Perry et al., 2020 | Not reported | "Key outcome markers include four subjective and objective markers of financial strain. The first three measure respondents' reported level of housing insecurity, food insecurity, and general financial insecurity attributed to the COVID-19 pandemic. Housing insecurity, food insecurity, and general financial insecurity were measured in wave 2 by asking respondents the extent to which they agreed that COVID-19 has made them worry that they "may not have a place to live," that they "may not have enough money to buy food," and "about their finances, in general" (0 = strongly disagree, 1 = disagree, 2 = agree, and 3 = strongly agree)" |
| Pooler and Gleason, 2014 | WIC receipt | "Dependent variables of interest in this study included the full use of WIC benefits overall and for each of the 14 WIC food categories. To determine whether benefits were used fully, the same method was applied to each of the 14 food categories:" |
| Rank and Hirsch, 2009 | Receipt of food stamps in the past 12 months | "Food stamp use was derived from a series of questions asked by the PSID interviewers as to whether the household had received specific cash or in-kind public assistance programs at some point during the prior year. With regard to food stamps, respondents were asked, "Did you (or anyone else in your family) use government food stamps at any time in [prior year]?" |
| Reeder et al., 2020 | Six-Item Short Form of the Food Security Survey Module | Food security status was measured using the United States Department of Agriculture's U.S. Household Food Security Survey Module: Six-Item Short Form [26]. This survey uses a subset of questions from the standard 18-item Food Security Survey Module. The Six-Item Short Form is a reliable substitute for the 18-item Food Security Survey Module and has a lower respondent burden. |
| Ruprecht et al., 2020 | Not reported | "Food shortage" |
| Harare et al., 2020 | Not reported | "Drawing on the food insecurity literature 12−15 a number of potential community-level sociodemographic variables were reviewed that may differentiate the two-by-two typology" |
| Sharkey and Schoenberg, 2002 | Nutrition Screening Initiative (NSI)& 10-item DETERMINE Checklist | "Nutritional risk was assessed by administering the Nutrition Screening Initiative (NSI)'s 10-item DETERMINE Checklist. The Checklist was developed as a self-report preliminary screen for warning signs of increased risk for poor nutritional health and is used by the ENP in most states for nutrition risk screening" |
| Sharkey and Schoenberg, 2005 | Food Sufficiency Status based on four self-reported risk situations that were related to absence of food and forced scarce-resource decisions | "The level of food sufficiency, during the 6months prior to the in-home assessment, was operationalized from four self-reported risk situations that were related to absence of food and forced scarce-resource decisions and that were previously used in national evaluation of OAANP to characterize the difficulty in meeting basic food needs among homebound meal program participants" |

| | | |
|---|---|---|
| Siddiqi et al., 2021 | Six-Item Short Form of the Food Security Survey Module | "We assessed food security in 2018 and 2020 using the validated Adult Food Security Survey Module Six-Item Short Form.21 We created a 3-level food security measure based on the survey module scores: we categorized people with scores ranging from 0 to 1.0 as being food secure, people with scores ranging from 2.0 to 4.0 as having a low level of food security, and people with scores ranging from 5.0 to 6.0 as having a very low level of food security. We based scores on the number of affirmative responses to the 6 questions included in the survey module" |
| Stallings et al., 2016 | Enrollment in Farmer's Market Nutrition Plan (FMNP) | "In addition to food vouchers and nutrition education, most states' WIC programs offer the Farmers' Market Nutrition Program (FMNP), which provides FMNP coupons to be redeemed at a WIC-approved farmers' market as a means to increase fresh F&V consumption" |
| Stewart et al., 2011 | 2007 AIDS Alabama Needs Assessment Survey | "The interview covered demographics; basic needs such as food, transportation, and housing; and a wide range of other ancillary services. A total of 14 basic and ancillary service needs were included: financial, legal, pharmaceutical, employment, and housing assistance; substance use, mental health, and alcohol treatment; medical services, transportation, dentalcare, case management, food, and childcare" |
| Stockman et al., 2020 | WHO survey tool on COVID stressors | "We assessed nine COVID-19 stressors as outlined by the WHO [20]. Examples include food insecurity, insufficient rent, and caregiver status. We employed the Fear of COVID-19 Scale, a 7-item scale, self-reported measure of an individual's fear of COVID-19" |
| Stuff et al., 2004 | Eighteen-Item Household Food Security Scale | "This interview included the 18-question US Food Security Survey Module and had questions about participation in nutrition assistance programs and income" |
| Tackett et al., 2018 | Eighteen-Item Household Food Security Scale | "National Health and Nutrition Examination Survey—Household Food Security Questionnaire. This 18-item questionnaire characterizes household food security status. Items assessed food access problems, limitations, food sufficiency, or food shortage" |
| Tamar goes et al., 2021 | U.S. Household Food Security Survey Module | "FI was determined with the Household Food Security Module, which assesses a respondent's perceived food sufficiency and adequacy during the past 12 months" |
| Tan et al., 2020 | U.S. Household Food Security Survey Module | "Food insecurity, which were assessed using the Household Food Security Survey (HFSS) module, a validated scale considered the reference measure of food security in the United States (22, 23). This 18-item scale captures uncertainty about food supply, sufficiency of food quantity, and diet quality over the previous 12 months" |
| Tong et al., 2019 | Six-Item Short Form of the Food Security Survey Module | "We assessed food security using the Six-Item Short Form of the US Household Food Security Survey Module (FSSM), a validated measure of food security in the general population and among older adults" |

| | | |
|---|---|---|
| Trego et al., 2019 | U.S. Adult Food Security Survey Module | "Food insecurity, the dependent variable, was measured by the 10-item adult US Food Security Survey Module (FSSM). The FSSM is designed to assess household financial ability to meet food needs within the past 12 months using Likert scale and yes/no questions" |
| Tucker-Seeley et al., 2016 | Health and Retirement Study (HRS) Food Insecurity question | "The items from the HRS were selected across five domains of hardship based on the poverty and life stress literatures as well as factors revealed in previous investigations of the measurement properties of hardship indicators: financial hardship, food hardship, housing/neighborhood hardship, employment instability, and medical need" |
| Vedovato et al., 2016 | Eighteen-Item Household Food Security Scale | "Food security was assessed using the US Department of Agriculture's eighteen-item Household Food Security Scale for households with children under 18 years old. Household food security was determined by the number of food-insecure conditions and reported behaviors reported in the past 12 months" |
| Walker et al., 2020 | U.S. Household Food Security Survey Module | "During each year of the survey, the same validated scale for food insecurity was used. Ten questions were asked of each family using questions and response options from the US Department of Agriculture Food Security Survey Module. A 4-level food security status was calculated based on the number of affirmative responses using scoring from Bickel et al" |
| Wang et al., 2015 | First item of the Household Food Insecurity Access Scale | "To capture the broadest domain of food insecurity—uncertainty about food access— VACS incorporated the first question of the 18-item Household Food Insecurity Access Scale (HFIAS) in the baseline patient survey" |
| Wang et al., 2021 | Six-Item Short Form of the Food Security Survey Module | "Our primary outcome was food security. We scored the 6-item version of the US Department of Agriculture's US Household Food Security Survey Module: Six-Item Short Form as a dichotomous variable: food secure (0 or 1 item answered affirmatively) or food insecure (2–6 items answered affirmatively)" |
| Weaver et al., 2019 | U.S. Household Food Security Survey Module | "The questionnaire used the United States Department of Agriculture (USDA) food security measure to assess food security. Over time, the measure has been modified and expanded and, most recently, includes a 10-item index" |
| Whitbeck et al., 2006 | 3-item adaptation from the USDA Food Insecurity Scale | "We assessed food insecurity with three items adapted from the USDA food insecurity scale that dealt specifically with going without or cutting back food. It should be noted that these are only three items from a 16-item scale, and they do not reflect the nuances of the full USDA measure" |
| Wilson et al., 2006 | U.S. Household Food Security Survey Module | "The 16-item US FSSM was verbally administered to each HD participant. Respondents answered often true, sometimes true, or never true to each item" |
| Wolfe et al., 1996 | Not reported | Not reported |

| | | |
|---|---|---|
| Wood and Harris, 2018 | Not reported | “Food insecurity was a dichotomous measure based on students indicating challenges with “hunger,” an acute form of food insecurity” |
| Yu et al., 2010 | Eighteen-Item Household Food Security Scale | “Child Food Security (the dependent variable) and Household Food Security (independent variable) are measured using items taken from the USDA 18-item Core Food Security Module (CFSM). Nine items from the CFSM use household food security. In the past12 months, households were asked whether: 1) they worried their food would run out before they got money to buy food; 2) the food that they bought just didn't last and they didn't have money to get more; 3) they couldn't afford to eat balanced meals; 4) they relied on only on a few kinds of low-cost food to feed their children because they were running out of money to buy food; 5) they ever cut the size of their meals or skip meals because there wasn't not enough money for food; 6) they ever ate less than they felt they should because there wasn’t enough money to for food; 7) they were ever hungry but didn’t eat because they couldn't afford enough food; 8) they lost weight because they didn't have enough money for food; and 9) they ever not eat for a whole day because there wasn't enough money for food” |

*Table 5. Citation characteristics (sorted by state, region, study design, and risk factors examined)*

| Citation | State(s) | Region | Study Design |
|---|---|---|---|
| Ahluwalia et al., 2013 | Not Reported | Not Reported | Cross-sectional |
| Baek, 2016 | Not Reported | Urban | Cross-sectional |
| Baer et al., 2015 | Massachusetts | Urban | Cross-sectional |
| Balistreri, 2016 | Not Reported | Not Reported | Cohort (longitudinal) |
| Barnidge et al., 2017 | Missouri | Not Reported | Cross-sectional |
| Barnidge et al., 2017 | Missouri | Both (urban and rural) | Concept mapping |
| Boone-Heinonen et al., 2015 | Minnesota, California, Alabama, and Illinois | Not Reported | Cohort (longitudinal) |
| Borders et al., 2015 | Illinois | Urban | Cohort (longitudinal) |
| Brewer et al., 2010 | Georgia | Not Reported | Cross-sectional |
| Burke et al., 2018 | South Carolina | Both (urban and rural) | Cross-sectional |
| Caraballo et al., 2020 | Not Reported | Not Reported | Cross-sectional |
| Chakrabarti et al., 2021 | All 50 US states + DC | Not Reported | Cross-sectional |

| | | | |
|---|---|---|---|
| Chilton and Booth, 2007 | Pennsylvania | Not Reported | Qualitative |
| Clay and Ross, 2020 | Texas | Both | Cross-sectional |
| Conlon et al., 2015 | New York | Urban | Randomized controlled trial |
| Cook et al., 2002 | Minnesota, Maryland, California, Massachusetts, Arkansas, and Washington D.C. | Urban | Cohort (longitudinal) |
| Cox and Wallace, 2016 | Not Reported | Not Reported | Cohort (longitudinal) |
| Crabtree and Mushi-Brunt, 2013 | Not Reported | Both (urban and rural) | Cross-sectional |
| Dean et al., 2011 | Texas | Rural | Cross-sectional |
| Denney et al., 2020 | Not Reported | Not Reported | Cross-sectional |
| Duke et al., 2021 | North Carolina | Urban | Cross-sectional |
| Duke, 2021 | Minnesota | Not Reported | Cross-sectional |
| Echeverria et al., 2004 | New York | Urban | Cross-sectional |
| Fleming et al., 2021 | Not Reported | Not Reported | Cross-sectional |
| Garrett-Peters and Mills-Koonce, 2013 | North Carolina and Pennsylvania | Rural | Cross-sectional |
| Ghosh and Parish, 2015 | Not Reported | Not Reported | Cohort (longitudinal) |
| Gilbert and Ashley, 2020 | Not Reported | Urban | Cross-sectional |
| Gilbert et al., 2017 | Maryland | Not Reported | Cross-sectional |
| Hanmer et al., 2021 | Not Reported | Not Reported | Cross-sectional |
| Harrison et al., 2005 | California | Not Reported | Cross-sectional |
| Hernandez and Pressler, 2013 | Not Reported | Not Reported | Cohort (longitudinal) |
| Huang et al., 2015 | Not Reported | Not Reported | Cohort (longitudinal) |
| Kaiser et al., 2007 | California | Not Reported | Cross-sectional |
| Keene et al., 2015 | Not Reported | Urban | Cross-sectional |
| Kharmats et al., 2014 | Maryland | Urban | Cross-sectional |
| Kipke et al., 2020 | California | Urban | Cross-sectional |
| Koh et al., 2020 | Ohio | Urban | Cross-sectional |
| Laraia et al., 2006 | North Carolina | Not Reported | Cross-sectional |
| Laraia et al., 2009 | North Carolina | Not Reported | Cross-sectional |
| Lauren et al., 2021 | Not Reported | Not Reported | Cross-sectional |

| | | | |
|---|---|---|---|
| Lee et al., 2011 | Georgia | Both (urban and rural) | Cohort (longitudinal) |
| Leigh and Medel-Herrero, 2015 | California | Not Reported | Cross-sectional |
| Liese et al., 2021 | South Carolina | Urban | Cohort (longitudinal) |
| Lin et al., 2021 | Not Reported | Not Reported | Cross-sectional |
| Lombe et al., 2009 | Not Reported | Not Reported | Cross-sectional |
| Long et al., 2020 | Not Reported | Not Reported | Cross-sectional |
| Makelarski et al., 2015 | Illinois | Urban | Cross-sectional |
| Martin et al., 2004 | Connecticut | Urban | Cross-sectional |
| Martin et al., 2016 | Connecticut | Urban | Randomized controlled trial |
| McCurdy and Metallinos-Katsaras, 2011 | Massachusetts | Not Reported | Cohort (longitudinal) |
| McDonough et al., 2019 | Not Reported | Not Reported | Cohort (longitudinal) |
| Miller et al., 1996 | Missouri and Illinois | Urban | Cross-sectional |
| Miller et al., 2015 | Kansas | Urban | Cross-sectional |
| Mook et al., 2016 | California | Urban | Cross-sectional |
| Moore et al., 2020 | Texas | Urban | Cross-sectional |
| Morales et al., 2020 | All 50 US states + DC | Not Reported | Cross-sectional |
| Murimi et al., 2016 | Texas | Both (urban and rural) | Cross-sectional |
| Myers and Painter, 2017 | Not Reported | Not Reported | Cross-sectional |
| Myers et al., 2020 | Not Reported | Not Reported | Cross-sectional |
| Nagata et al., 2021 | Not Reported | Not Reported | Cross-sectional |
| Nam et al., 2014 | Oklahoma | Not Reported | Cross-sectional |
| Okafor et al., 2020 | Connecticut | Not Reported | Cross-sectional |
| O'Reilly et al., 2020 | Not Reported | Urban | Cross-sectional |
| Orozoco et al., 2020 | Not Reported | Not Reported | Cross-sectional |
| Palmer et al., 2018 | Iowa | Both (urban and rural) | Cross-sectional |
| Palmer et al., 2020 | Iowa | Not Reported | Cross-sectional |
| Paschal et al., 2020 | Alabama | Both (urban and rural) | Cross-sectional |
| Patterson et al., 2020 | Not Reported | Not Reported | Cross-sectional |
| Payne-Sturges et al., 2018 | Not Reported | Not Reported | Cross-sectional |

| | | | |
|---|---|---|---|
| Perry et al., 2020 | Indiana | Both (urban and rural) | Cohort (longitudinal) |
| Pooler and Gleason, 2014 | Michigan | Both (urban and rural) | Cross-sectional |
| Rank and Hirschl, 2009 | Not Reported | Not Reported | Cohort (longitudinal) |
| Reeder et al., 2020 | Mississippi | Not Reported | Cross-sectional |
| Ruprecht et al., 2020 | Illinois | Urban | Cross-sectional |
| Sharareh et al., 2020 | Utah | Not Reported | Cross-sectional |
| Sharkey and Schoenberg, 2002 | North Carolina | Not Reported | Cross-sectional |
| Sharkey and Schoenberg, 2005 | North Carolina | Not Reported | Cross-sectional |
| Siddiqi et al., 2021 | Pennsylvania | Urban | Cohort (longitudinal) |
| Stallings et al., 2016 | Georgia | Urban | Randomized controlled trial |
| Stewart et al., 2011 | Alabama | Rural | Cross-sectional |
| Stockman et al., 2020 | Not Reported | Both (urban and rural) | Cross-sectional |
| Stuff et al., 2004 | Louisiana, Mississippi, and Arkansas | Not Reported | Cross-sectional |
| Tackett et al., 2018 | Not Reported | Not Reported | Cross-sectional |
| Tamargo et al., 2021 | Florida | Urban | Cohort (longitudinal) |
| Tan et al., 2020 | Not Reported | Not Reported | Cross-sectional |
| Tong et al., 2019 | California | Not Reported | Cross-sectional |
| Trego et al., 2019 | Not Reported | Not Reported | Cross-sectional |
| Tucker-Seeley et al., 2016 | Not Reported | Not Reported | Cohort (longitudinal) |
| Vedovato et al., 2016 | Maryland | Urban | Cross-sectional |
| Walker et al., 2020 | Not Reported | Not Reported | Cross-sectional |
| Wang et al., 2015 | Not Reported | Not Reported | Cohort (longitudinal) |
| Wang et al., 2021 | California | Urban | Cohort (longitudinal) |
| Weaver et al., 2019 | New Jersey | Not Reported | Cross-sectional |
| Whitbeck et al., 2006 | Kansas, Missouri, Iowa, and Nebraska | Urban | Cross-sectional |
| Wilson et al., 2006 | Louisiana | Not Reported | Cross-sectional |
| Wolfe et al., 1996 | New York | Both (urban and rural) | Qualitative |
| Wood and Harris, 2018 | California | Not Reported | Cross-sectional |

| | | | |
|---|---|---|---|
| Yu et al., 2010 | Not Reported | Not Reported | Cross-sectional |

*Table 6. Spread of study participants' ages (sorted by lower and upper age limits, number of study participants, and number of households)*

| Citation | Lower age limit | Upper age limit | Central tendency | Total African Americans | Total Participants | Total African American households | Total households |
|---|---|---|---|---|---|---|---|
| Ahluwalia et al., 2013 | Not reported | Not reported | Not reported | 2384 | 14478 | 2384 | 14478 |
| Baek, 2016 | Not reported | Not reported | Not reported | Not reported | 28304 | Not reported | 28304 |
| Baer et al., 2015 | 15 | 25 | Mean: 18 | 216 | 400 | 216 | 400 |
| Balistreri, 2016 | Not reported | Not reported | Not reported | Not reported | 263,779 | Not reported | 263,779 |
| Barnidge et al., 2017 | Not reported | Not reported | Median: 31 | 160 | 212 | 160 | 212 |
| Barnidge et al., 2017 | Not reported | Not reported | Median: 37 | 38 | 38 | 38 | 38 |
| Boone-Heinonen et al., 2015 | 18 | 30 | Not reported | 2038 | 4174 | 2038 | 4174 |
| Borders et al., 2015 | Not reported | Not reported | Not reported | 55 | 112 | 55 | 112 |
| Brewer et al., 2010 | Not reported | Not reported | Median: 76 | 220 | 621 | 220 | 621 |
| Burke et al., 2018 | Not reported | Not reported | Mean: 40.8 | 154 | 194 | 154 | 194 |
| Caraballo et al., 2020 | 18 | 64 | Mean: 51.6 | 1781 | 8967 | Not-reported | Not-reported |
| Chakrabarti et al., 2021 | Not-reported | Not-reported | Mean: 51.55 | 86062 | 1088314 | Not-reported | Not-reported |
| Chilton and Booth, 2007 | 25 | 60 | Mean: 45 | 34 | 34 | 34 | 34 |
| Clay and Ross, 2020 | 18 | 64 | Not-reported | 101 | 1002 | 101 | 1002 |
| Conlon et al., 2015 | 22 | 67 | Mean: 37.1 | 54 | 301 | 54 | 301 |
| Cook et al., 2002 | Not reported | Not reported | Not reported | 1872 | 2178 | 1872 | 2178 |

| | | | | | | | |
|---|---|---|---|---|---|---|---|
| Cox and Wallace, 2016 | Not reported | Not reported | Not reported | Not reported | 4898 | Not reported | Not reported |
| Crabtree and Mushi-Brunt, 2013 | 18 | 99 | Mean: 63.92 | 1140 | 4672 | 1140 | 4672 |
| Dean et al., 2011 | Not reported | Not reported | Mean: 64.1 | Not reported | 1059 | Not reported | 1059 |
| Denney et al., 2020 | Not-reported | Not-reported | Mean: 33.49 | 1290 | 8600 | 1290 | 8600 |
| Duke et al., 2021 | 18 | 48 | mean: 20.1 | 320 | 351 | Not-reported | Not-reported |
| Duke, 2021 | 18 | 19 | Mean: 14.8 | Not-reported | 644 | Not-reported | Not-reported |
| Echeverria et al., 2004 | Not reported | Not reported | Mean: 38.4 | Not reported | 48 | Not reported | 48 |
| Fleming et al., 2021 | 13 | 18 | Median: 15 | 1207 | 4777 | Not-reported | Not-reported |
| Garrett-Peters and Mills-Koonce, 2013 | Not reported | Not reported | Not reported | Not reported | Not reported | Not reported | Not reported |
| Ghosh and Parish, 2015 | Not reported | Not reported | Not reported | 2049 | 9919 | 2049 | 9919 |
| Gilbert and Ashley, 2020 | Not-reported | Not-reported | Mean: 47.6 | 365 | 498 | 365 | 498 |
| Gilbert et al., 2017 | Not reported | Not reported | Mean: 26.8 | 8928 | 23065 | 8928 | 23065 |
| Hanmer et al., 2021 | 18 | 75 | Not-reported | 467 | 4142 | Not-reported | Not-reported |
| Harrison et al., 2005 | 18 | Not reported | Not reported | Not reported | 2926000 | Not reported | Not reported |
| Hernandez and Pressler, 2013 | Not reported | Not reported | Mean: 28.8 | 396 | 1650 | 396 | 1650 |
| Huang et al., 2015 | Not reported | Not reported | Mean: 39.94 | 4120 | 18263 | 4120 | 18263 |
| Kaiser et al., 2007 | 18 | 98 | Not reported | 252 | 4037 | 252 | 4037 |
| Keene et al., 2015 | Not reported | Not reported | Not reported | 905 | 905 | 905 | 905 |

| | | | | | | | |
|---|---|---|---|---|---|---|---|
| Kharmats et al., 2014 | 22 | 89 | Mean: 47.6 | 244 | 362 | 244 | 362 |
| Kipke et al., 2020 | 16 | 24 | Mean: 22.3 | 94 | 448 | Not-reported | Not-reported |
| Koh et al., 2020 | 18 | 65 | Not-reported | 148 | 586 | 148 | 586 |
| Laraia et al., 2006 | 16 | 45 | Mean: 27.2 | 201 | 606 | 201 | 606 |
| Laraia et al., 2009 | 18 | 35 | Not reported | 206 | 206 | 206 | 206 |
| Lauren et al., 2021 | 18 | 65 | Not-reported | 73 | 1250 | 73 | 1250 |
| Lee et al., 2011 | Not reported | Not reported | Mean: 74.6 | 238 | 717 | 238 | 717 |
| Leigh and Medel-Herrero, 2015 | Not reported | Not reported | Not reported | Not reported | 40,896 | Not reported | 40,896 |
| Liese et al., 2021 | Not-reported | Not-reported | Mean: 54.1 | 373 | 397 | 373 | 397 |
| Lin et al., 2021 | 18 | 49 | Not-reported | 39 | 554 | 39 | 554 |
| Lombe et al., 2009 | 18 | 80 | Mean: 45.2 | 3104 | 23360 | 3104 | 23360 |
| Long et al., 2020 | 18 | not-reported | Not-reported | 5056 | 38860 | 5056 | 38860 |
| Makelarski et al., 2015 | Not reported | Not reported | Not reported | 87 | 200 | 87 | 200 |
| Martin et al., 2004 | Not reported | Not reported | Not reported | 145 | 330 | 145 | 330 |
| Martin et al., 2016 | Not reported | Not reported | Mean: 51.4 | 164 | 227 | 164 | 227 |
| McCurdy and Metallinos-Katsaras, 2011 | Not reported | Not reported | Not reported | 3049 | 18039 | 3049 | 18039 |
| McDonough et al., 2019 | Not-reported | not-reported | Not-reported | 642 | 6822 | 642 | 6822 |
| Miller et al., 1996 | Not reported | Not reported | Not reported | 416 | 416 | 416 | 416 |
| Miller et al., 2015 | Not reported | Not reported | Not reported | Not reported | 177,688 | Not reported | Not reported |

| | | | | | | | |
|---|---|---|---|---|---|---|---|
| Mook et al., 2016 | Not reported | Not reported | Not reported | 272 | 531 | 272 | 531 |
| Moore et al., 2020 | 18 | not-reported | Not-reported | 90 | 602 | 90 | 602 |
| Morales et al., 2020 | Not-reported | Not-reported | Mean: 48.236 | 8546 | 74413 | 8546 | 74413 |
| Murimi et al., 2016 | Not reported | Not reported | Not reported | 62 | 191 | 62 | 191 |
| Myers and Painter, 2017 | Not reported | Not reported | Mean: 46.21 | 5762 | 32464 | 5762 | 32464 |
| Myers et al., 2020 | 20 | Not-reported | Mean: 46.9 | 5389 | 46145 | 5389 | 46145 |
| Nagata et al., 2021 | Not-reported | Not-reported | mean: 48.46 | 6985 | 63674 | 6985 | 63674 |
| Nam et al., 2014 | Not reported | Not reported | Not reported | 465 | 2652 | 465 | 2652 |
| Okafor et al., 2020 | 1.08 | 94 | Not-reported | 388 | 1299 | 388 | 1299 |
| O'Reilly et al., 2020 | Not-reported | Not-reported | Not-reported | 409 | 450 | 409 | 450 |
| Orozoco et al., 2020 | Not-reported | Not-reported | Not-reported | 417 | 2069 | 417 | 2069 |
| Palmer et al., 2018 | Not reported | Not reported | Mean: 34.7 | 14 | 36 | 14 | 36 |
| Palmer et al., 2020 | 19 | 50 | Mean: 34.7 | 14 | 36 | 14 | 36 |
| Paschal et al., 2020 | 45 | 65 | Not-reported | 102 | 102 | 102 | 102 |
| Patterson et al., 2020 | 18 | 59 | Not-reported | 11211 | 52702 | 11211 | 52702 |
| Payne-Sturges et al., 2018 | Not reported | Not reported | Mean: 20.69 | 20 | 237 | 20 | 237 |
| Perry et al., 2020 | Not-reported | Not-reported | Mean: 45.77 | 89 | 994 | 89 | 994 |
| Pooler and Gleason, 2014 | Not reported | Not reported | Not reported | 40230 | 152794 | 40230 | 152794 |
| Rank and Hirschl, 2009 | 1 | 20 | Not reported | Not reported | Not reported | Not reported | Not reported |

| | | | | | | | |
|---|---|---|---|---|---|---|---|
| Reeder et al., 2020 | 18 | 24 | Mean: 19.77 | 24 | 131 | Not-reported | Not-reported |
| Ruprecht et al., 2020 | 21 | 70 | Not-reported | 64 | 107 | 64 | 107 |
| Sharareh et al., 2020 | Not-reported | Not-reported | Not-reported | Not-reported | Not-reported | Not-reported | Not-reported |
| Sharkey and Schoenberg, 2002 | 60 | 103 | Mean: 79 | 335 | 729 | 335 | 729 |
| Sharkey and Schoenberg, 2005 | 61 | 98 | Median: 79 | 125 | 268 | 125 | 268 |
| Siddiqi et al., 2021 | Not-reported | Not-reported | Mean: 62 | 537 | 598 | 537 | 598 |
| Stallings et al., 2016 | Not reported | Not reported | Not reported | 148 | 149 | 148 | 149 |
| Stewart et al., 2011 | 18 | 76 | Mean: 42.5 | 364 | 476 | 364 | 476 |
| Stockman et al., 2020 | 18 | Not-reported | Median: 33 | 60 | 473 | 60 | 473 |
| Stuff et al., 2004 | Not reported | Not reported | Not reported | 807 | 1662 | 807 | 1662 |
| Tackett et al., 2018 | Not reported | Not reported | Not reported | 40 | 183 | 40 | 183 |
| Tamargo et al., 2021 | Not-reported | Not-reported | Mean: 53.9 | 283 | 394 | 283 | 394 |
| Tan et al., 2020 | Not-reported | Not-reported | Median: 49.6 | 914 | 1324 | 914 | 1324 |
| Tong et al., 2019 | Not reported | Not reported | Median: 58 | 279 | 350 | Not applicable | Not applicable |
| Trego et al., 2019 | 20 | Not reported | Not reported | 2616 | 11220 | Not reported | Not reported |
| Tucker-Seeley et al., 2016 | Not reported | Not reported | Mean: 66.6 | 415 | 3074 | 415 | 3074 |
| Vedovato et al., 2016 | Not reported | Not reported | Not reported | 298 | 298 | 298 | 298 |
| Walker et al., 2020 | 18 | 65 | Not-reported | 36099 | 287836 | 36099 | 287836 |
| Wang et al., 2015 | Not reported | Not reported | Not reported | 4336 | 6709 | 4336 | 6709 |
| Wang et al., 2021 | Not-reported | 79 | Not-reported | 80 | 213 | 80 | 213 |

| | | | | | | | |
|---|---|---|---|---|---|---|---|
| Weaver et al., 2019 | Not reported | Not reported | Mean: 20.9 | 216 | 2055 | 216 | 2055 |
| Whitbeck et al., 2006 | 16 | 19 | Mean: 17.4 | 94 | 428 | Not applicable | Not applicable |
| Wilson et al., 2006 | Not reported | Not reported | Mean: 59.1 | 55 | 98 | 55 | 98 |
| Wolfe et al., 1996 | 60 | 89 | Not reported | 16 | 41 | 16 | 35 |
| Wood and Harris, 2018 | 18 | Not reported | Not reported | 718 | 6103 | 718 | 6103 |
| Yu et al., 2010 | 18 | 79 | Mean: 35.2 | 710 | 3799 | 710 | 3799 |

## Synthesis of results

Data were extracted from ninety-eight citations. Seventy-three studies employed a cross-sectional design, while the remaining studies were as follows: cohort/longitudinal (n = 19 studies), randomized controlled trial (n = 3 studies), qualitative (n = 2 studies), and concept mapping (n = 1 study). Studies were conducted in many states, but many authors did not report the state (n= 35). Twenty-eight studies reported findings from urban, both urban and rural (n = 12 studies), and rural (n = 3 studies) regions while the remaining studies did not report a specific region (n = 55 studies).

For the 115 risk factors identified, demographic characteristics represented the majority of factors described in the literature (n = 53 factors). Behavioural (lifestyle and nutritional habits, n = 5 factors), environmental (physical and social environment, n = 38 factors), health-related characteristics (physical and mental health, n = 15 factors), and COVID-19 related risk factors (n = 4) were less commonly reported. For possible consequences of food insecurity (n = 92 factors), the following terms received the greatest number of hits across the reviewed citations: self-reported health status (n = 16 citation hits), total number of people in household

(n = 14 citation hits), SNAP recipient (n = 14 citation hits), depression or depressive symptoms (n = 12 citation hits), and body mass index (BMI) (n = 8 citation hits).

The results of the risk factor mapping process are presented in Figure 2. Dimensions of Food Insecurity Evidence and Gap Map (EGM). The 115 risk factors were mapped to five broad categories (demographic, behaviour, environment, health-related factors, and COVID-19 related factors) along with ten descriptive subcategories for further risk factor categorization. Each subcategory was further mapped to the three dimensions of food security (food accessibility, availability, and utilization) and each combination available (1. Accessibility and Availability; 2. All Categories; 3. Accessibility; 4. Accessibility and Utilization; 5. Availability (Figure 2). None of the identified risk factors mapped to food utilization exclusively, so this category was not represented in the figure. Demographic factors mapped most frequently to the accessibility category while household and economic factors mapped to the food accessibility and utilization categories. Behavioural factors linked to behaviour and nutrition mapped to all three dimensions of food security, while COVID-19 related factors and health-related factors primarily mapped to food accessibility and utilization. Most of the physical environmental factors mapped to food accessibility and availability, while most social environmental factors mapped to food accessibility exclusively. Ultimately, this EGM provides a visual breakdown of risk factor categorization across each dimension and possible combination of food security in all included studies (Figure 2).

Thirty-seven measures of food security were identified across 98 citations. Most authors implemented the U.S. Household Food Security Survey Module (n = 16), the Six-Item Short Form of the Food Security Survey Module (n = 16), and the Eighteen-Item Household Food Security Scale (n = 13). The remaining studies referenced other measures of food security (Table 4). Adaptations of the USDA Food Security Survey Module included the US Adult Food Security Survey Module, a 2-item screener derived from the 18-Item US Household Food

Security Screen, and a 3-Iitem adaptation from the USDA Food Insecurity Scale [17-19]. Non-USDA metrics included the National Health Interview Survey on Disability, the 2007 AIDS Alabama Needs Assessment Survey, the Survey of Income and Program Participation (SIPP), the Food Insufficiency Indicator (from SEED OK Survey), the Current Population Survey Food Security Supplement (CPS-FSS), the Health and Retirement Study (HRS) Food Insecurity Questionnaire, the Radimer-Cornell Hunger and Food Insecurity Instrument, the Access to Healthy Foods Scale, and the NHANES Food Security Module [21-29]. Remaining metrics include Food Sufficiency Status based on four self-reported risk situations that were related to absence of food and forced scarce-resource decisions, neighbourhood supermarket density per 10,000 people, receipt of food stamps in the past 12 months, the number of full-service retail food outlets (RFOs) in the neighbourhood, and WIC receipt [30-33].

Most of the demographic factors (n = 53 risk factors), including household and economic terms, were mapped to the food access category (n = 52 risk factors) while remaining dimensions of food security, food availability (n = 5 risk factors) and food utilization (n = 26 risk factors), were mapped less frequently (Table 7). Examples of identified demographic risk factors include age, race/ethnicity, gender, number of children in household, socioeconomic status (SES), and family poverty. All behavioural factors (n = 5), including lifestyle habits and terms linked to nutrition, mapped to food access and food utilization (Table 8). Most of the environmental factors (n = 38 factors), including physical and social environment terms, mapped to the food access category (n = 36 factors), while food availability (n = 19 factors) and food utilization (n = 10 factors), were mapped less frequently (Table 9). Examples of identified environmental risk factors include geographic location, living situation, neighbourhood grocery store availability, and neighbourhood safety from crime and violence. All health-related factors (n = 15), including physical and mental health terms, mapped to the food access dimension of food insecurity. Most of these terms also mapped to the food utilization category (n = 13) while

none of them mapped to food availability (Table 10). Examples of identified health-related risk factors include human immunodeficiency virus (HIV) status, arthritis, alcoholism, liver fibrosis, and health insurance status. All COVID-19 related risk factors (n = 4), including impact of COVID-19 on employment, stay-at-home orders, decreased income due to COVID-19, and unemployed prior to pandemic, mapped to the food access and utilization dimension of food security (Table 11).

*Table 7. Demographic risk factors mapped to the dimensions of food insecurity*

| Term | Citation Hits | Sub Category | Accessibility | Availability | Utilization | Level |
|---|---|---|---|---|---|---|
| Race/ethnicity | 65 | Demographic | Accessibility | | | Individual |
| Age | 54 | Demographic | Accessibility | | | Individual |
| Education | 52 | Demographic | Accessibility | | | Individual |
| Gender (social) | 41 | Demographic | Accessibility | | | Individual |
| Household income | 29 | Economic | Accessibility | | Utilization | Group |
| Employed/Unemployed | 28 | Economic | Accessibility | | Utilization | Individual |
| Marital status (partnered status) | 28 | Household | Accessibility | | Utilization | Group |
| Number of children in household | 20 | Household | Accessibility | | Utilization | Group |
| Income | 15 | Economic | Accessibility | | Utilization | Individual |
| Family poverty | 11 | Economic | Accessibility | Availability | Utilization | Group |
| Child's age | 10 | Household | Accessibility | | Utilization | Group |
| Race | 10 | Demographic | Accessibility | | | Individual |
| Single parent (status) | 6 | Household | Accessibility | | Utilization | Group |
| Time (year) | 6 | Demographic | Accessibility | Availability | | Group |
| Mother's age | 5 | Household | Accessibility | | Utilization | Group |
| Child's gender | 4 | Household | Accessibility | | | Individual |
| Female-headed household | 4 | Household | Accessibility | | | Group |
| Home ownership | 4 | Economic | Accessibility | | Utilization | Individual |

| | | | | | | |
|---|---|---|---|---|---|---|
| Documentation status (work permit, citizen, legal permanent resident, etc.) | 3 | Demographic | Accessibility | | | Individual |
| Poverty rate | 3 | Economic | Accessibility | Availability | Utilization | Group |
| Sexual orientation | 3 | Demographic | Accessibility | | | Individual |
| Unemployment rate | 3 | Economic | Accessibility | | | Group |
| Disability | 2 | Demographic | Accessibility | | Utilization | Individual |
| Family monthly poverty level index | 2 | Economic | Accessibility | | | Group |
| History of military service | 2 | Demographic | Accessibility | | | Individual |
| Hours of work | 2 | Economic | Accessibility | | Utilization | Individual |
| Infant/child race/ethnicity | 2 | Household | Accessibility | | | Individual |
| Maternal union transitions | 2 | Economic | Accessibility | | | Individual |
| Pregnant woman (pregnancy status) | 2 | Demographic | Accessibility | | Utilization | Individual |
| Baby's father in household | 1 | Household | Accessibility | | Utilization | Group |
| Baby's grandmother in household | 1 | Household | Accessibility | | Utilization | Group |
| Bank account ownership | 1 | Economic | Accessibility | | | Individual |
| Child in household on NSLP (National School Lunch Program) | 1 | Household | | Availability | | Both |
| Credit card ownership | 1 | Economic | Accessibility | | | Individual |

| | | | | | | |
|---|---|---|---|---|---|---|
| Disabled adults in household | 1 | Household | Accessibility | | Utilization | Group |
| Disabled child in household (not receiving SSI) | 1 | Household | Accessibility | | Utilization | Group |
| Disabled child in household (receiving SSI) | 1 | Household | Accessibility | | Utilization | Group |
| English proficiency | 1 | Demographic | Accessibility | | | Individual |
| Financial capability | 1 | Economic | Accessibility | | Utilization | Both |
| Financial hardship from medical bills | 1 | Economic | Accessibility | | | Both |
| Gender modality (transgender or cisgender) | 1 | Demographic | Accessibility | | | Individual |
| Has dependents | 1 | Household | Accessibility | | Utilization | Individual |
| Have enough money to buy food at the hospital | 1 | Economic | Accessibility | | | Individual |
| History of incarceration | 1 | Household | Accessibility | | Utilization | Both |
| Income insecurity | 1 | Economic | Accessibility | Availability | Utilization | Both |
| Parental drug use | 1 | Household | Accessibility | | Utilization | Group |
| Parental incarceration | 1 | Household | Accessibility | | Utilization | Group |
| Religion | 1 | Demographic | Accessibility | | | Individual |
| Senior in household | 1 | Household | Accessibility | | Utilization | Group |
| Socio-economic status (SES) | 1 | Economic | Accessibility | | | Individual |
| State welfare expenditures | 1 | Economic | Accessibility | | | Group |

| | | | | | | |
|---|---|---|---|---|---|---|
| Unexpected expenses | 1 | Economic | Accessibility | | | Individual |
| Will lose income from your job because of hospital stay | 1 | Economic | Accessibility | | | Individual |

*Table 8. Behavioural risk factors mapped to the dimensions of food insecurity*

| Term | Citation Hits | Sub Category | Accessibility | Availability | Utilization | Level |
|---|---|---|---|---|---|---|
| Drug problem | 3 | Behavioral | Accessibility | | Utilization | Individual |
| "I'm too busy to take the time to prepare healthy foods" | 2 | Nutrition | Accessibility | | Utilization | Individual |
| SNAP receipt in past year | 2 | Nutrition | Accessibility | | Utilization | Individual |
| Time since SNAP distribution | 1 | Nutrition | Accessibility | | Utilization | Individual |
| Taking prescribed medications | 1 | Behavioral | Accessibility | | Utilization | Individual |

*Table 9. Environmental risk factors mapped to the dimensions of food insecurity*

| Term | Citation Hits | Sub Category | Accessibility | Availability | Utilization | Level |
|---|---|---|---|---|---|---|
| Urbanicity | 7 | Physical Environment | Accessibility | Availability | | Group |
| Access to car | 5 | Physical Environment | Accessibility | | | Both |
| Living situation (living alone vs with spouse/family/room-mates) | 4 | Physical Environment | Accessibility | | Utilization | Both |

| | | | | | | |
|---|---|---|---|---|---|---|
| Social support (to borrow money from) | 4 | Social Environment | Accessibility | | | Individual |
| Access to help from family, friends, neighbors | 3 | Social Environment | Accessibility | | Utilization | Individual |
| Geographic location | 2 | Physical Environment | Accessibility | Availability | | Group |
| State | 2 | Physical Environment | Accessibility | Availability | | Group |
| Social capital | 2 | Social Environment | Accessibility | | | Individual |
| Metropolitan residency | 1 | Physical Environment | Accessibility | Availability | | Group |
| Fruit and vegetable selection in neighborhood | 1 | Physical Environment | Accessibility | Availability | | Group |
| Have transportation to get food while at the hospital | 1 | Physical Environment | Accessibility | | | Individual |
| Neighborhood ascetic quality | 1 | Physical Environment | Accessibility | Availability | | Group |
| Neighborhood walking/exercise environment | 1 | Physical Environment | Accessibility | | | Group |
| Neighborhood safety from crime/violence | 1 | Physical Environment | Accessibility | Availability | | Group |
| Neighborhood grocery store availability | 1 | Physical Environment | | Availability | | Group |
| Ambient (environmental temperature) | 1 | Physical Environment | Accessibility | Availability | | Group |
| Birthplace (inside vs outside US) | 1 | Physical Environment | Accessibility | | | Individual |
| Calendar month | 1 | Physical Environment | Accessibility | | | Individual |
| Patterns of food source destinations | 1 | Physical Environment | | Availability | | Group |

| | | | | | | |
|---|---|---|---|---|---|---|
| Home damage | 1 | Physical Environment | Accessibility | Availability | Utilization | Both |
| Relocation | 1 | Physical Environment | Accessibility | Availability | Utilization | Both |
| Disaster assistance | 1 | Physical Environment | Accessibility | Availability | Utilization | Both |
| Spatial access | 1 | Physical Environment | Accessibility | Availability | | Both |
| Transportation mode | 1 | Physical Environment | Accessibility | | | Individual |
| Shopping distance | 1 | Physical Environment | Accessibility | Availability | Utilization | Both |
| Member of social or civic organization | 1 | Social Environment | Accessibility | | | Individual |
| Personal disparity | 1 | Social Environment | Accessibility | | | Individual |
| Number of people in social network | 1 | Social Environment | Accessibility | | Utilization | Individual |
| Church (community characteristic) | 1 | Social Environment | Accessibility | Availability | | Both |
| Neighborhood participation index | 1 | Social Environment | Accessibility | | Utilization | Group |
| Neighborhood social cohesion | 1 | Social Environment | Accessibility | | Utilization | Group |
| Neighborhood problems index | 1 | Social Environment | Accessibility | Availability | | Group |
| Lifetime racial discrimination | 1 | Social Environment | Accessibility | | | Individual |
| Neighborhood congruence | 1 | Social Environment | Accessibility | | | Group |
| Neighborhood SES | 1 | Social Environment | Accessibility | Availability | | Group |
| Neighborhood race/ethnic statuses | 1 | Social Environment | Accessibility | Availability | | Group |
| Sense of community | 1 | Social Environment | Accessibility | | Utilization | Group |
| SNAP policy change | 1 | Social Environment | Accessibility | | | Group |

333

*Table 10. Health-Related risk factors mapped to the dimensions of food insecurity*

| Term | Citation Hits | Sub Category | Accessibility | Availability | Utilization | Level |
|---|---|---|---|---|---|---|
| Health insurance status | 4 | Physical Health | Accessibility | | Utilization | Both |
| Impairment that limits/prevents use of public transportation | 2 | Physical Health | Accessibility | | Utilization | Individual |
| Alcoholism | 2 | Mental Health | Accessibility | | Utilization | Individual |
| Cancer type | 1 | Physical Health | Accessibility | | Utilization | Individual |
| Time since cancer diagnosis | 1 | Physical Health | Accessibility | | Utilization | Individual |
| Difficulty walking | 1 | Physical Health | Accessibility | | | Individual |
| Difficulty sitting | 1 | Physical Health | Accessibility | | | Individual |
| Difficulty standing | 1 | Physical Health | Accessibility | | Utilization | Individual |
| Difficulty lifting/carrying (10lbs) | 1 | Physical Health | Accessibility | | Utilization | Individual |
| Length of time on dialysis | 1 | Physical Health | Accessibility | | Utilization | Individual |
| HIV status | 1 | Physical Health | Accessibility | | Utilization | Individual |
| Arthritis | 1 | Physical Health | Accessibility | | Utilization | Individual |
| Joint pain | 1 | Physical Health | Accessibility | | Utilization | Individual |
| Liver fibrosis | 1 | Physical Health | Accessibility | | Utilization | Individual |
| Mastery score | 1 | Mental Health | Accessibility | | Utilization | Individual |

*Table 11. COVID-19 related risk factors mapped to the dimensions of food insecurity*

| Term | Citation Hits | Sub-category | Accessibility | Availability | Utilization | Level |
|---|---|---|---|---|---|---|
| Impact of COVID-19 on Employment | 1 | COVID-19 | Accessibility | | Utilization | Individual |
| State stay-at-home orders | 1 | COVID-19 | Accessibility | | Utilization | Group |

| | | | | | | |
|---|---|---|---|---|---|---|
| Decreased income (COVID-19) | 1 | COVID-19 | Accessibility | | Utilization | Both |
| Unemployed (prior to pandemic) | 1 | COVID-19 | Accessibility | | Utilization | Individual |

# Discussion

## Summary of the evidence

The findings from this evidence and gap map suggest that a wide range of risk factors have been evaluated for an association with food insecurity among African American adults across the peer-reviewed literature. Demographic and environmental categories represented the greatest number of risk factors evaluated across studies, which suggests that these categories, and relevant terms within each group, have received adequate representation across studies.

COVID-19 related factors (n = 4), behavioural factors (n = 5), and health-related factors (n = 15) comprised the fewest number of risk factors across included studies, which serves as a significant gap compared to demographic and environmental characteristics. It is critical for researchers to consider risk factor representation to fill knowledge gaps by examining behavioural and health-related risk factors among African American adults in future studies. A few examples include sexual orientation [20], English proficiency [32], pregnancy status [34-35], religion [36], lifetime racial discrimination [15], neighbourhood safety from crime and violence [24], neighbourhood grocery store availability [36], impairment that limits use of public transportation [22, 37], HIV status [38], decreased income due to COVID [39], impact of COVID-19 on employment, and stay-at-home orders [40]. Future primary research studies could focus on these under-represented risk factors that may perpetuate food insecurity among African American adults. The inference obtained from a single estimate is limited, therefore authors of future studies should consider the information from the small number of conducted

studies to refine behavioural metrics and improve study design for stronger inference about associations.

Diverse measures have been employed across this body of included studies to measure food insecurity. The use of multiple measures presents issues for understanding the entire body of work. If researchers and clinicians are willing to modify standardized measures of food security, then a justification for this modification must be reported. For example, the 2-item screen derived from the 18-Item US Household Food Security Screen could impact the accuracy of the measurement of food insecurity. In addition, it is important for researchers and clinicians to consider the value or weight of individual questions within modified screeners. Variation in questions and similar themes could lead to distinct differences between metrics of food security. The authors of this evidence and gap map encourage researchers to utilize standardized metrics in addition to any questionnaire modification they desire, so that the body of work has a standard for comparison. Efforts such as the Core Outcome Measures in Effectiveness Trials Initiative (COMET) have been working towards standardizing outcomes as a means of reducing research wastage [41]. The rationale for using standard outcomes is that it facilitates comparison between studies. Of course, inclusion of a standard outcome like the USDA 18-item questionnaire, is not a barrier to adding additional outcomes that researchers are interested in investigating.

Results from this evidence and gap map also suggest that the three hierarchical dimensions of food security (food accessibility, availability, and utilization) are represented by distinct risk factor categories across the peer-reviewed literature and are not equally evaluated. It is critical for researchers to acknowledge that risk factors linked to food accessibility have received adequate risk factor representation across the published literature and that other dimensions of food security, food availability and food utilization, must be explored to better serve African American adults who experience barriers linked to food insecurity.

Another gap includes the absence of synthesized results for risk factors that have received adequate study representation across the peer-reviewed literature. Multiple demographic risk factors including education, age, race/ethnicity, and gender were assessed for an association with food insecurity among all 62 included studies. A systematic review of these risk factors might expose which demographic factors are associated with the highest risk of food insecurity among members of the population of interest.

Another characteristic was the frequent use of cross-sectional study designs (n = 73) compared to cohort or longitudinal study designs (n = 19) and randomized controlled trials (n = 3). As noted by multiple authors of the included studies, the use of the cross-sectional design limits the assertion of a causal relationship between exposure variables and outcomes of interest [22]. However, there is an opportunity to consider the implementation of other designs such as cohort study designs. The value that could be obtained from studying groups that do not experience food insecurity and then become food insecure would eliminate many of the limitations of trying to understand the cause and effect presented across the peer-reviewed literature.

## Limitations

The focus of this EGM was on peer-reviewed literature, and it is unclear if inclusion of grey literature would have impacted the findings.

## Conclusions

The findings from this evidence and gap map suggest that metrics of food security and risk factors associated with food insecurity among African American adults have received variable levels of representation across the literature. It is critical for the research community to consider the wide range of food security metrics that exist and how the creation of new metrics or modification of standardized metrics could impact the ability to synthesize the

findings in this critical area. It is also crucial that researchers consider risk factor areas that have already been extensively studied and that are eligible for systematic reviews (education, age, race/ethnicity, and gender) as they consider the next steps in this area. Resources for research are always limited but replication is critical. However, it behoves researches to ensure that evaluating the same risk factor again will add as much value as adding a new factor if costs are involved in data collection. For example, behavioural risk factors and risk factors mapped to the food availability dimension of food security require further investigation to better assess barriers that impact African American populations in the United States. Other underrepresented risk factors to consider for future research include factors linked to health disparities among African American adults: lifetime racial discrimination, neighbourhood grocery store availability, neighbourhood safety from violence, and income insecurity. It is crucial for authors to consider the impact of these factors and how they relate to forms of systemic racism and the COVID-19 pandemic in the United States. Currently, there is a potential to conduct systematic reviews on these topics and summarize the associations found across multiple populations.

1995-2019 literature and potential covid linkage is weak

# References


1. Coleman-Jensen A, Rabbitt MP, Gregory CA, and Singh A. Household food security in the United States in 2019. *United States Department of Agriculture*, Economic Research Service. 2020. https://www.ers.usda.gov/webdocs/publications/99282/err-275.pdf?v=6912.2.
2. Hake M, Dewey A, Engelhard E, Strayer M, Harper TJ, Summerfelt T, Malone-Smolla C, and Maebry T. The impact of the Coronavirus on food insecurity in 2020. *Feeding America*. 2020; https://www.feedingamerica.org/sites/default/files/2020-10/Brief_Local%20Impact_10.2020_0.pdf
3. Kamdar N, Rozmus CL, Grimes DE, and Meininger JC. Ethnic/Racial Comparisons in strategies parents use to cope with food insecurity: a systematic review of published

research. *Journal of Immigrant and Minority Health*. 2018; (21), 175-188. https://doi.org/10.1007/s10903-018-0720-y.

4. Laraia BA, Borja JB, and Bentley ME. Grandmothers, fathers and depressive symptoms are associated with food insecurity among low- income first-time African American mothers in North Carolina. *The American Dietetic Association*. 2009; 109(6). https://doi.org/10.1016/j.jada.2009.03.005.
5. Vedovato GM, Surkan PJ, Jones-Smith J, Anderson-Steeves E, Han E, Trude ACB, Kharmats AY, and Gittelsohn J. Food insecurity, overweight and obesity among low-income African-American families in Baltimore city: Associations with food-related perceptions. *Public Health Nutrition*. 2015; 19(8). https://doi.org/10.1017/S1368980015002888.
6. Ashby S, Kleve S, McKechnie R, and Palermo C. Measurement of the dimensions of food insecurity in developed countries: a systematic review. *Public Health Nutrition*. 2016; 19(16), 2887-2896. https://doi.org/10.1017/S1368980016001166.
7. Tricco AC, Lillie E., Zarin W, O'Brien KK, Colquhoun H, Levac D, Moher D, Peters MD, Horsley T, Weeks L, Hempel S. PRISMA extension for scoping reviews (PRISMA-ScR): Checklist and explanation. Annals of Internal Medicine. 2018; 169(7), 467-473. doi:10.7326/M18-0850.
8. Munn Z, Peters MD, Stern C, Tufanaru C, McArthur A, and Aromataris E. Systematic review or scoping review? Guidance for authors when choosing between a systematic or scoping review approach. *BMC Medical Research Methodology*. 2018; 18(143). https://doi.org/10.1186/s12874-018-0611-x.
9. Arksey H and O'Malley L. Scoping studies: Towards a methodological framework. *International Journal of Social Research Methodology*. 2005; 8(1), 19–32. https://doi.org/10.1080/1364557032000119616.

10. United States Census Bureau. Race, about this topic. *United States Census Bureau*. 2018. https://www.census.gov/topics/population/race/about.html.

11. Rastogi S, Johnson TD, Hoeffel EM, and Drewery Jr. MP. The Black population: 2010, 2010 Census Briefs. The United States Department of Commerce. *The United States Census Bureau*. 2011; 1-20. https://www.census.gov/prod/cen2010/briefs/c2010br-06.pdf.

12. McGowan J, Sampson M, Salzwedel DM, Cogo E, Foerster V, and Lefebvre C. PRESS peer review of electronic search strategies: 2015 guideline statement. *Journal of Clinical Epidemiology*. 2016; 75, 40–46. https://doi.org/10.1016/j.jclinepi.2016.01.021.

13. United States Department of Agriculture (USDA). Food security in the United States., Survey Tools. *Economic Research Service*. 2019. https://www.ers.usda.gov/topics/food-nutrition-assistance/food-security-in-the-us/survey-tools/.

14. Page MJ, McKenzie JE, Bossuyt PM, Boutron I, Hoffmann TC, Mulrow CD, et al. The PRISMA 2020 statement: an updated guideline for reporting systematic reviews. BMJ 2021;372:n71. doi: 10.1136/bmj.n71.

15. Barnidge E, LaBarge G, Krupsky K, and Arthur A. Screening for Food Insecurity in Paediatric Clinical Settings: Opportunities and Barriers. *The Journal of Community Health*. 2017; 42(1), 51-57. doi: 10.1007/s10900-016-0229-z.

16. Burke MP, Jones SJ, Frongillo EA, Maryah SF, Blake CE, and Freedman DA. Severity of household food insecurity and lifetime racial discrimination among African-American households in South Carolina. *Ethnicity and Health*. 2018; 23(3), 276-292. https://doi.org/10.1080/13557858.2016.1263286.

17. Tong M, Tieu L, Lee CT, Ponath C, Guzman D, and Kushel M. Factors associated with food insecurity among older homeless adults: results from the HOPE HOME study. *Journal of Public Health*. 2019; 41(2), 240-249. https://doi.org/10.1093/pubmed/fdy063.

18. Kharmats AY, Jones-Smith JC, Sang CY, Budd N, Flamm L, Cuccia A, Mui Y, Trude A, and Gittelsohn J. Relation between the Supplemental Nutritional Assistance Program cycle and dietary quality in low-income African Americans in Baltimore, Maryland. *American Journal of Clinical Nutrition*. 2014; 99(5), 1006-1014. https://doi.org/10.3945/ajcn.113.075994.

19. Makelarski JA, Thorngren D, and Lindau ST. Feed First, Ask Questions Later: Alleviating and Understanding Caregiver Food Insecurity in an Urban Children's Hospital. *American Journal of Public Health. 2015;* 105(8), e98-e104. doi:10.2105/AJPH.2015.302719.

20. Whitbeck LB, Chen X, and Johnson KD. Food insecurity among homeless and runaway adolescents. Public Health Nutrition. 2006; 9(1), 47-52. https://doi.org/10.1079/PHN2005764.

21. Baek D. The Effect of Public Transportation Accessibility on Food Insecurity. *The Eastern Economic Journal*. 2016; 42(1), 104-134. https://doi.org/10.1057/eej.2014.62.

22. Crabtree JL and Mushi-Brunt C. Public Transportation to Obtain Food: An Overlooked Instrumental Activity of Daily Living. *Occupation, Participation, and Health*. 2013; 33(4), 209-217. https://doi.org/10.3928/15394492-20130912-05.

23. Dean WR, Sharkey JR, and Johnson CM. Food insecurity is associated with social capital, perceived personal disparity, and partnership status among older and senior adults in a largely rural area of central Texas. *The Journal of Nutrition in Gerontology and Geriatrics*. 2011; 30(2), 169-186. https://doi.org/10.1080/21551197.2011.567955

24. Echeverria SE, Diez-Roux AV, and Link BG. Reliability of self-reported neighbourhood characteristics. Journal of Urban Health: Bulletin of the New York Academy of Medicine. 2004; 81(4), 682-701. https://doi.org/10.1093/jurban/jth151.

25. Keene DE and Geronimus AT. Community-based support among African American public housing residents. *Journal of Urban Health*. 2011 88(1), 41-53. doi:10.1007/s11524-010-9511-z.

26. Myers AM and Painter MA. Food insecurity in the United States of America: an examination of race/ethnicity and nativity. Food Security. 2017; 9(6), 1419-1432. https://doi.org/10.1007/s12571-017-0733-8.

27. Nam Y, Huang J, Heflin C, and Sherraden M. Racial and Ethnic Disparities in Food Insufficiency: Evidence from a State-wide Probability Sample. Journal of the Society for Social Work and Research. 2015; 6(2), 201-228. doi: 10.1086/681574.

28. Stewart KE, Phillips MM, Walker JF, Harvey SA, and Porter A. Social services utilization and need among a community sample of persons living with HIV in the rural south. AIDS Care. 2011; 23(3), 340-347. https://doi.org/10.1080/09540121.2010.507743.

29. Tucker-Seeley RD, Marshall G, and Yang F. Hardship among older adults in the HRS: Exploring measurement differences across socio-demographic characteristics. Race and Social Problems. 2016; 8(3), 222-230. https://doi.org/10.1007/s12552-016-9180-y.

30. Boone-Heinonen J, Green-Howard A, Meyer K, Lewis C.E., Kiefe C.I., Laroche H.H., Gunderson E.P., and Gordon-Larsen P. Marriage and parenthood in relation to obesogenic neighbourhood trajectories: The CARDIA study. *Health and Place*. 2015; 34, 229-240. https://doi.org/10.1016/j.healthplace.2015.05.005.

31. Miller M, Middendorf G, and Wood SD. Food Availability in the Heartland: Exploring the Effects of Neighbourhood Racial and Income Composition. Rural Sociology. 2015; 80(3), 340-361. https://doi.org/10.1111/ruso.12063.

32. Pooler J and Gleason SF. Comparison of WIC Benefit Redemptions in Michigan Indicates Higher Utilization Among Arab American Families. Journal of Nutrition Education and Behaviour. 2014; S45-52. 10.1016/j.jneb.2014.02.019

33. Sharkey JR and Schoenberg NE. Prospective study of black-white differences in food insufficiency among homebound elders. Journal of Aging and Health. 2005; 17(4), 507-527. https://doi.org/10.1177/0898264305279009.

34. Harrison GG, Manolo-LeClair G, Ramirez A, Chia YJ, Kurata J, McGarvey N, and Sharp M. More than 2.9 million Californians now food insecure--one in three low-income, an increase in just two years. *Policy Brief UCLA Centre for Health Policy Research*. 2005; Pb2005-6, 1-11. https://escholarship.org/uc/item/678449g9.

35. Kaiser L, Baumrind N, and Dumbauld S. Who is food-insecure in California? Findings from the California Women's Health Survey, 2004. Public Health Nutrition. 2007; 10(6), 574-581. https://doi.org/10.1017/S1368980007382542.

36. Wolfe WS, Olson CM, Kendall A, and Frongillo EA. Understanding food insecurity in the elderly: A conceptual framework. Journal of Nutrition Education. 1996; 28(2), 92-100. https://doi.org/10.1016/S0022-3182(96)70034-1.

37. Wood JL and Harris F. Experiences With "Acute" Food Insecurity Among College Students. Educational Researcher. 2018; 47(2). https://doi.org/10.3102/0013189X17752928.

38. Wang EA, McGinnis KA, Goulet J, Bryant K, Gibert C, Leaf DA, Mattocks K, Fiellin LE, Vogenthaler N, Justice AC, and Fiellin DA. Food insecurity and health: data from

the Veterans Aging Cohort Study. *Public Health Reports*. 2015; 130(3), 261-268. https://doi.org/10.1177/003335491513000313.

39. Lin TK, Law R, Beaman J, and Foster DG. The impact of the COVID-19 pandemic on economic security and pregnancy intentions among people at risk of pregnancy. *Contraception*. 2021; 103 (6), 380-385. https://doi.org/10.1016/j.contraception.2021.02.001
40. Lauren BN, Silver ER, Faye AS, Rogers AM, Woo-Baidal JA, Ozanne EM, and Hur C. Predictors of households at risk for food insecurity in the United States during the COVID-19 pandemic. *Public Health Nutrition*. 2021; 1-8. https://doi.org/10.1017/S1368980021000355
41. COMET Initiative. Core outcome measures in effectiveness trials. *COMET Initiative*. 2020. http://www.comet-initiative.org/.
42. Ghosh S and Parish SL. Deprivation among U.S. children with disabilities who receive supplemental security income. Journal of Disability Policy Studies. 2015; 26(3), 173-183. https://doi.org/10.1177/1044207314539011.
43. Lombe M, Yu M, and Nebbitt VE. Assessing effects of food stamp program participation on food security in female-headed households: do informal supports matter? Journal of Policy Practice. 2009; 8(4), 301-316. https://doi.org/10.1080/15588740903176278.

# Supporting Information

**Appendix A: Screening Forms**

**Title and abstract screening:** Title/Abstract screening was performed using the following questions, with response options “yes”, “no”, or “unclear”:

1. Is the title/abstract available in English?

    a. Yes, include and proceed to Q2 (If there is no abstract but the title is in English, answer “Yes”)
    b. No, exclude; (specify language) _______________
2. Is the primary research study describing food (in)security metrics among African American adults in the United States?
    a. Yes, include and proceed to full-text screening.
    b. No, exclude.
    c. Unclear, proceed to full-text screening.
3. Comments ___________________

**Full-text screening:** Full-text screening was performed using the following questions:

1. Is the full text available in English?
    a. Yes, include and proceed to next question.
    b. No, exclude; (specify language) ___________
2. Does the full-text article describe a primary research study?
    a. Yes, include and proceed to next question.
    b. No, exclude.
3. Does the full-text article include the population of interest (African American adults in the United States)?
    a. Yes, include/proceed to next question.
    b. No, exclude.
4. Does the full text evaluate food (in)security or potential metrics of food insecurity such as availability (NOT just availability only in the home but outside the home), supply, intake, deprivation, utilization, or use of Food Assistance Programs?
    a. Yes, include and proceed to next question.
    b. No, exclude.

5. Does the study design have a comparison group?
    a. Yes, include and proceed to next question.
    b. No, exclude.
6. Does the study assess individual- or group-level factors associated with food insecurity?
    a. Yes, proceed to data extraction.
    b. No, exclude.
7. Comments ________________

**Data Extraction Strategy**

**Study Characteristics**

1. Reference ID #
2. What is the Study ID (If the article describes only 1 study, this is the same as the Reference ID#)?
3. Indicate the state in which the population resides
4. Indicate the region(s) (urban or rural) in which the population resides
5. Enter the spread of the age of the participants
    - Range
    - Standard Deviation
    - Standard Error
    - 95% Confidence Interval
    - Standard Deviation/Standard Error not specified
    - Not Reported
    - IQR
6. Enter the central tendency of the age of the participants
    - Mean

        - Median
        - Not Reported

7. What is the number of participants of the population of interest in the study? (Give the number of African American participants)?
8. What is the total number of participants in the study (including the ones who are not African American)?
9. What is the total number of African American households sampled in the study?
10. What is the total number of households in the study (including the ones that are not African American)?
11. What was the study design?
12. Additional Comments

**Food Security Outcomes**

1. Reference ID #
2. What is the Study ID (If the article describes only 1 study, this is the same as the Reference ID#)?
3. What is the metric of food insecurity reported?
4. What was the authors' exact definition of the food insecurity metrics?
5. What exposures/risk factors were examined?
6. Which dimensions of food security (accessibility, availability, and utilization) are represented by each risk factor identified?
7. Additional Comments

Figure 1 Click here to access/download;Figure;PRISMA 2020_Figure 1.tiff

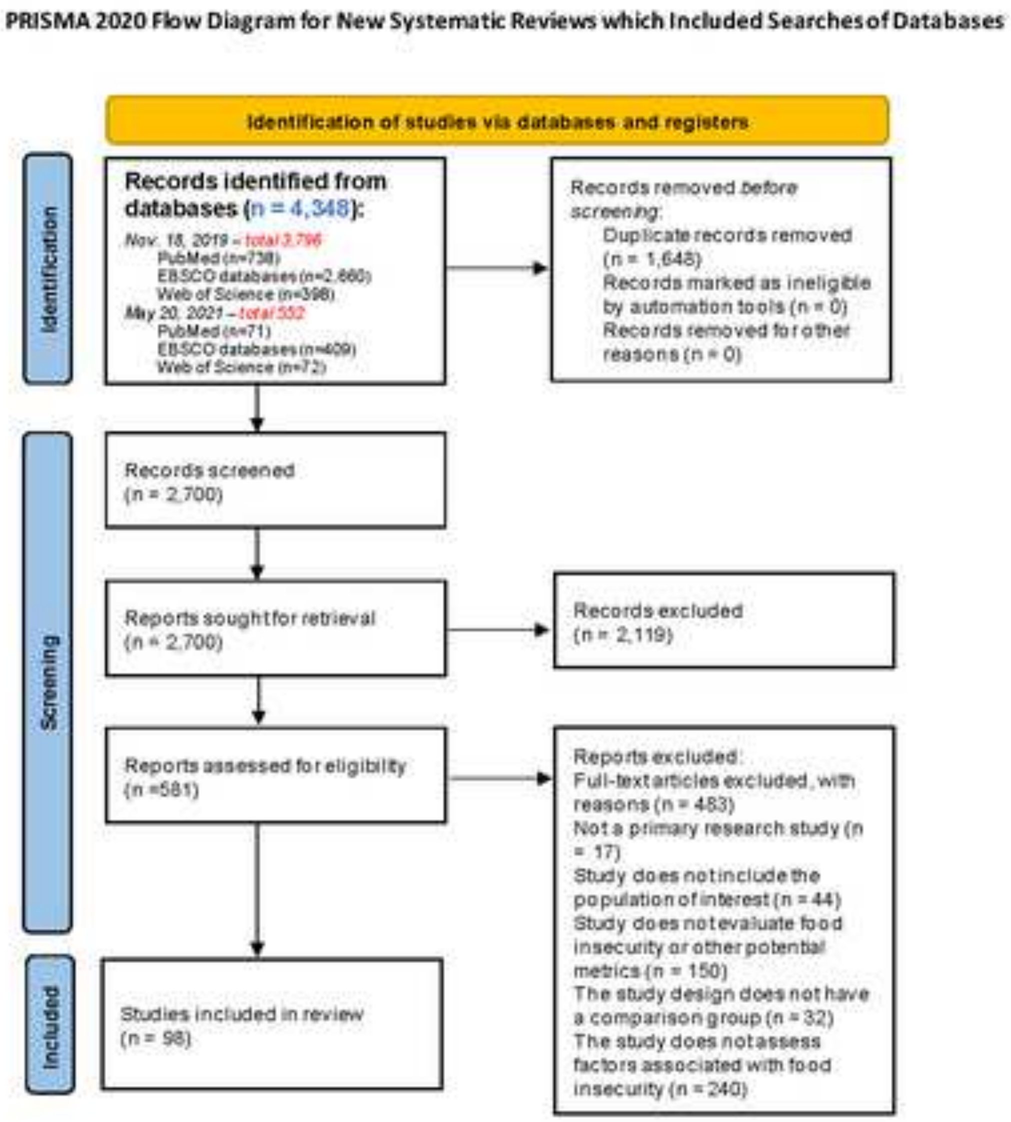

Figure 2

Click here to access/download;Figure;Final_EGM_2.tiff

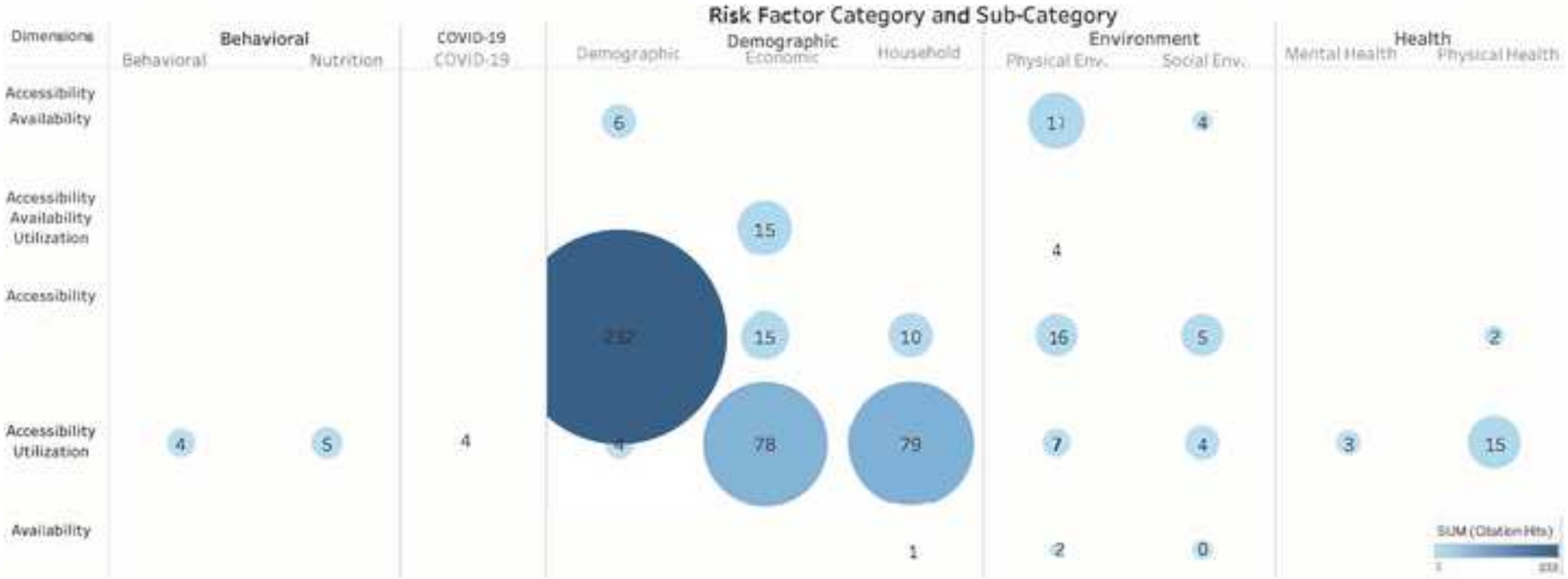

Click here to access/download
**Supporting Information**
2021_06_25_Campbell MECCIR Reporting Standards.docx

PRISMA ScR Checklist

Click here to access/download
**Supporting Information**
2021_06_25_PRISMA Checklist.pdf
